# Extent of Structural Asymmetry in Homodimeric Proteins: Prevalence and Relevance

**DOI:** 10.1371/journal.pone.0036688

**Published:** 2012-05-22

**Authors:** Lakshmipuram Seshadri Swapna, Kuchi Srikeerthana, Narayanaswamy Srinivasan

**Affiliations:** Molecular Biophysics Unit, Indian Institute of Science, Bangalore, India; University of Cambridge, United Kingdom

## Abstract

Most homodimeric proteins have symmetric structure. Although symmetry is known to confer structural and functional advantage, asymmetric organization is also observed. Using a non-redundant dataset of 223 high-resolution crystal structures of biologically relevant homodimers, we address questions on the prevalence and significance of asymmetry. We used two measures to quantify global and interface asymmetry, and assess the correlation of several molecular and structural parameters with asymmetry. We have identified rare cases (11/223) of biologically relevant homodimers with pronounced global asymmetry. Asymmetry serves as a means to bring about 2∶1 binding between the homodimer and another molecule; it also enables cellular signalling arising from asymmetric macromolecular ligands such as DNA. Analysis of these cases reveals two possible mechanisms by which possible infinite array formation is prevented. In case of homodimers associating via non-topologically equivalent surfaces in their tertiary structures, ligand-dependent mechanisms are used. For stable dimers binding via large surfaces, ligand-dependent structural change regulates polymerisation/depolymerisation; for unstable dimers binding via smaller surfaces that are not evolutionarily well conserved, dimerisation occurs only in the presence of the ligand. In case of homodimers associating via interaction surfaces with parts of the surfaces topologically equivalent in the tertiary structures, steric hindrance serves as the preventive mechanism of infinite array. We also find that homodimers exhibiting grossly symmetric organization rarely exhibit either perfect local symmetry or high local asymmetry. Binding of small ligands at the interface does not cause any significant variation in interface asymmetry. However, identification of biologically relevant interface asymmetry in grossly symmetric homodimers is confounded by the presence of similar small magnitude changes caused due to artefacts of crystallisation. Our study provides new insights regarding accommodation of asymmetry in homodimers.

## Introduction

Symmetry is a prevailing feature in the global organisation of protein structures [1]. It is manifest in different levels: internal symmetry in tertiary structure (eg. folds of β-trefoil, TIM barrel, ferredoxin) [2], symmetric organisation in homomeric complexes (eg. HIV protease, vascular endothelial growth factor), pseudo-symmetric organisation of proteins containing subunits with similar tertiary structures (eg. haemoglobin), large-scale symmetric arrangement of repeating units (e.g., viral capsids) and symmetric arrangement of large number of subunits to form structural proteins (eg. actin filament).

In their excellent and comprehensive review on the role of symmetry in proteins, Goodsell and Olson list the various advantages of symmetry over asymmetry [1]. Symmetric organization provides co-operativity and multivalent binding. It also provides the ability to prevent infinite array formation, which is known to lead to disease conditions such as prion diseases and Alzheimer's [3]–[5]. Symmetric forms of homo-oligomers (homomers) are also postulated to provide highly stable complex structures for assembled protomers [6]. A separate study by Shakhnovich and coworkers also points to the universal phenomenon of statistically significant increased self-attraction between random surfaces [7], [8]. In contrast, Andre and co-workers attribute the overwhelming prevalence of symmetric oligomers to the availability of larger populations of low-energy symmetric complexes in the set of primordial complexes [9].

Homo-oligomers, which predominantly exhibit symmetric organisation [10], form an important component of the cellular system as they populate protein interaction networks and are found to occur much more often than by chance [11]. They also form about 50–70% of the available structural dataset [12], [13]. The 3DComplex database provides a symmetry-based classification system of all the available crystal structures solved [10]. A manually curated version of this database, PiQSi, provides an excellent complement containing information on biologically relevant complexes [14]. Large-scale studies on the conservation of homomeric interactions indicate that structural symmetry is well conserved in most homomers [13]. Further proof for the importance of symmetry is provided by the following large-scale analyses: internal symmetry is used as an alternative to homo-oligomerization [15], most of the ancient quaternary structures appear to be symmetrical than the more recently evolved quaternary structures [13]. Consequently, duplication of homomeric interactions coupled with the ability of paralogues to attract different partners has been postulated to lead to evolution of protein complexes [16].

Although several studies on the importance of symmetry in homooligomers have been undertaken, as listed above, the role of asymmetry in homooligomers is not well studied. Asymmetric organization, although rare, has been observed in certain protein assemblies, in order to perform specialized functions [1]. Of the homomeric complexes, homodimers predominate the bandwagon [1], [10]. Considering the wealth of structural data available for homodimers and their functional diversity [17], we study the prevalence and biological relevance of asymmetry in homodimeric proteins. In our study, we refer to “symmetry” and “asymmetry” in its mathematical sense rather than in the traditional sense used by structural biologists. In the traditional sense, any molecule can be categorised as grossly symmetric or grossly asymmetric based on its molecular symmetry. For example, the two subunits of homodimeric triose phosphate isomerase (TIM) molecule exhibit molecular symmetry in their organization. However, they exhibit a certain amount of asymmetry when compared at the level of individual atoms, rendering the molecule asymmetric in the mathematical sense. Obviously homomeric assembly within the asymmetric unit of a protein crystal lattice would indicate asymmetry according to mathematical definition of symmetry while symmetry characterized by crystallographic axes indicates perfect symmetry. Although the traditional definition based on molecular symmetry is extremely useful in describing the structural organization of biological molecules, the quantitative estimate of even minute asymmetry captured by the mathematical definition could provide some functional insights. Therefore, we have used the mathematical definition of symmetry in our study.

In this study we quantify the extent of global and interface (local) asymmetry in biologically relevant homodimeric proteins of known 3-D structure and ascertain functional implications of asymmetry. We also investigate how the possible infinite array of molecular assembly is avoided in the cases of homodimers with pronounced asymmetry.

## Results and Discussion

### Measures to quantify the extent of global asymmetry and local asymmetry at the interface of homodimeric complexes

**Figure 1:**
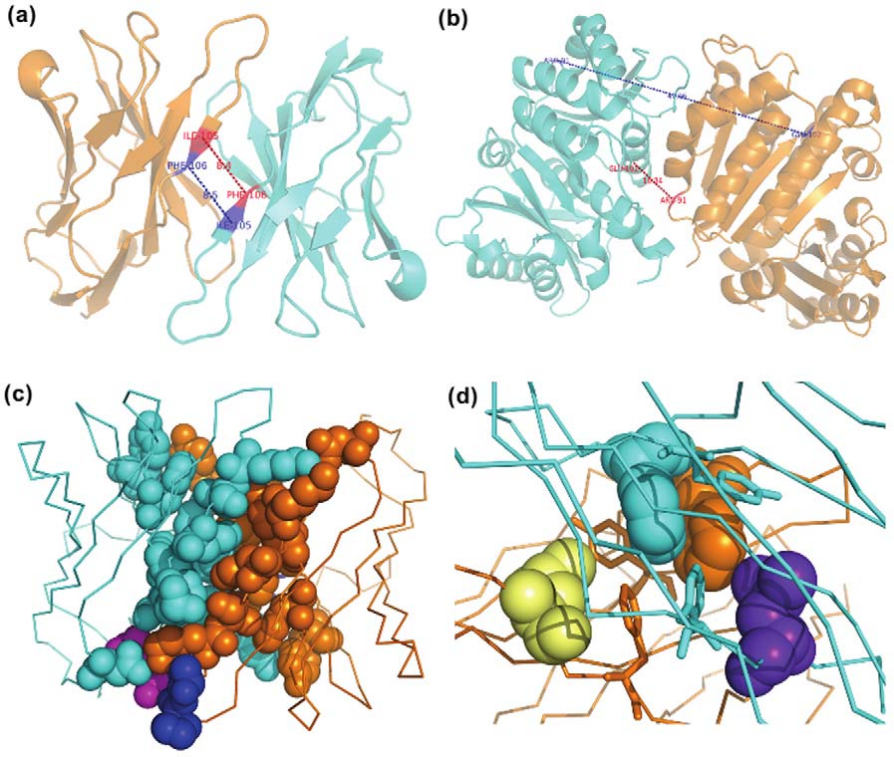
Global and interface asymmetry measures. Parameters used in the calculation of global and interface asymmetry scores. Figures ‘a’ and ‘b’ denote examples demonstrating the global asymmetry scores calculated for symmetric and asymmetric dimers, respectively. a). The figure depicts the two distances between Cα atoms for the pair of residues Ile-105 and Phe-106 in the dimeric *variable domain of T cell receptor delta chain* (PDB code: 1tvd). Chains A and B are colored as orange and cyan cartoons, respectively. The distance between Cα atoms of Ile-105(A)<->Phe-106(B) is shown in red and the corresponding distance between Cα atoms of Ile-105(B)<->Phe-106(A) is shown in blue. b). The figure depicts the two distances between Cα atoms for the pair of residues Arg-91 and Glu-102 in the dimeric *cell division protein FtsZ* (PDB code: 1rlu). Chains A and B are colored as orange and cyan cartoons, respectively. The distance between Cα atoms of Arg-91(A)<->Glu-102(B) is shown in red and the corresponding distance between Cα atoms of Arg-91(B)<->Glu-102(A) is shown in blue. Figures ‘c’ and ‘d’ highlight the information used for the calculation of interface asymmetry scores 1 and 2, respectively, for the case of the dimeric *variable domain of T cell receptor delta chain* (PDB code: 1tvd). Chains A and B are colored as orange and cyan ribbons, respectively. Interacting residues are depicted as spheres. c). Interface asymmetry score 1 is calculated by considering the fraction of unique interacting residues in the two chains. The unique interacting residues of chain A and chain B are shown in blue and magenta, respectively. d). Interface asymmetry score 2 is calculated by considering the fraction of unique interactions for a common interacting residue. The common interacting residue Phe-44 is shown as spheres. The set of interacting partner residues which are common in both chains are shown as sticks. The unique interacting residue present in Chain B is depicted as purple spheres. Its non-interacting counterpart in chain A is depicted as pale yellow spheres, to provide a picture of the difference in distance. All the figures of structures provided in this study have been generated using PyMoL [77].

The numerical measure (Figure 1a & 1b) used in this work provides a means to determine the extent of asymmetry observed in complexes. This measure of global asymmetry (GloA_Sc) proposed by Andre et al [9] can range from 0 to any number, with high values corresponding to high asymmetry. Visual inspection reveals that a score of 3 or lower can be considered as grossly symmetric complexes. Mapping of GloA_Sc with the crystallography-based symmetry classification measure provided by 3DComplex on the dataset of redundant homodimers reveals that a GloA_Sc of 5 or higher indicates complexes with pronounced asymmetry. A visual picture of the extent of global asymmetry corresponding to various scores can be gauged from examples shown in Figure S1.

### Homodimers are predominantly symmetric

The calculation of global asymmetry score for the non-redundant dataset of biologically relevant homodimers shows that an overwhelming number of homodimers have low global asymmetry (Table 1). Around 76% of the homo-dimers have GloA_Sc≤0.4 and almost ∼90% have Glo_Sc≤1. Visual inspection indicates that a score of ≤0.4 is an indicator of a highly symmetric homodimer. These results are in concurrence with previous reports on the prevalence of symmetry in homo-oligomers [1], [10], reflected by the presence of only 3% of biologically relevant asymmetric complexes in the current structural databases [12]. Around 5% of the homodimers have limited asymmetric organization (GloA_Sc between 1–3) and another 5% show gross profound asymmetry (GloA_Sc>5). In particular, eleven cases of very high global asymmetry (GloA_Sc≥7) have been listed in the present study as biologically relevant from the non-redundant dataset (Tables 2 & 3).

**Table 1:** Details of the distribution of global asymmetry scores for a non-redundant set of homodimers.

Global asymmetry score	Number of entries	Relative Frequency (%)	Entries with Cá-RMSD<0.5 between protomers (%)	Entries where 10% of residues contribute to top 25% of asymmetry (%)
0–0.2^#^	93	41.51	8.60	2.15
0.2–0.4^#^	78	34.82	3.84	7.69
0.4–0.6^#^	22	9.82	18.18	22.72
0.6–0.8^#^	9	4.01	0	22.22
0.8–1.0^#^	0	0	-	-
1.0–3.0^#^	11	4.91	0	0
3.0–5.0	6	2.67	0	0
5.0-10.0@	2	0.89	0	0
10.0-15.0@	1	0.44	0	0
15.0-20.0@	1	0.44	0	0
20.0-25.0@	1	0.44	0	0

Note: The entries with ‘#’ in the superscript exhibit gross global symmetry and the entries with ‘@’ in the superscript exhibit distinct global asymmetry.

**Table 2:** Details of functionally relevant homodimers exhibiting global asymmetry.

PDB code	Molecule	GloA_Sc	IntA_Sc1	IntA_Sc2	Interface area (Å^2^)	Stability assessment using PISA	Interface array formation	Biological relevance
2c2l	*Carboxy terminus of Hsp70-interacting protein* (CHIP)+C-terminal peptide of Hsp70	8.29	0.11	0.20	3830	CHIP homodimer is stable on its own	No – the dimer is symmetric in one part of the interface	Asymmetry at the interface coupled with the modified orientation of one the domains abolishes one of the two equivalent binding sites for ubiquitin conjugating enzyme. This provides a mechanism to achieve 2∶1 binding of a dimeric chaperone with a single ubiquitin conjugating system [28].
1knz	*NSP3 homodimer*+RNA fragment	8.57	0.34	0.70	7610	NSP3 homodimer is stable on its own	No – same surfaces are interacting	Asymmetric dimerization enables the creation of a single highly basic RNA binding tunnel, to bring about 2∶1 binding with 3′ end of rotaviral mRNA [21].
1hwt	*Heme activator protein+*2 diff molecules of DNA	9.08	0.42	0.34	2220	Heme activator protein dimer is stable on its own	No – Interface region is partially overlapping	DNA-induced asymmetric dimerization occurs due to the presence of direct-repeats of DNA half sites. The dimerization enables its function as a transcriptional activator for genes involved in oxidative phosphorylation and repair [44].
1f3m	*PAK1 autoregulatory domain*+PAK1 kinase domain	9.63	0.2	0.59	3680	Autoregulatory domain dimer is stable on its own	No – the dimer is symmetric at the interface and the rest of the molecule adopts an asymmetric orientation	Asymmetric dimer exists in autoinhibited conformation. A symmetric dimer would cause a few hydrophobic residues to be exposed, providing some support for asymmetry [29].
1rlu	*Cell division protein ftsZ+*GSP	10.33	0.84	1	3260	FtsZ dimer is stable on its own	No – same faces are interacting.	FtsZ polymerizes in a GTP-dependent manner to form the Z-ring, whose contraction is critical in cell division. The two chains assemble laterally [31].
1lq1	*Stage 0 sporulation protein A+*2 diff molecules of DNA	11.79	1	##	861.7	Dimer of sporulation protein A is not stable on its own	Probably not feasible since the dimer is not stable independently. Dimerization is probably dependent on presence of ligand.	Asymmetric dimerization is required for binding to direct DNA repeats. The Spo0A protein regulates around 500 genes in the sporulation process [53].
1mvo	*PhoP response regulato*	12.16	1	##	1013.7	The dimer is not stable.	No – might form closed group after some subunits	Presence of asymmetric interface correlates with experimental findings from DNA footprinting studies that there is cooperative binding of the dimers at PhoP-activated protomers. However this may not be the true interface [52].
1a6y	*Orphan nuclear receptor NR1D1+*2 diff molecules of DNA	15.49	1	##	346.8	Nuclear receptor dimer is not stable.	Probably not feasible since dimerization is ligand dependent.	It binds DNA containing direct repeats and functions as a transcriptional repressor. Dimerization is required to enable the molecule to bind with its corepressors [35].
1kb2	*Vitamin D3 receptor+*2 diff molecules of DNA	17.47	1	##	372.5	Vitamin D3 receptor dimer is not stable.	Probably not feasible since dimerization is ligand dependent.	A ligand-activated transcription factor that plays a central role in calcium homeostasis [39].
1jff	*Tubulin alpha and beta subunits*	23.09	1	##	3602	Dimer is stable	Prevented by ligand-mediated structural change.	Asymmetry-enabled filament structure essential for its function as cytoskeletal element [34].
1adv	*Adenovirus single-stranded DNA-binding protein*	23.42	1	##	2830	Dimer is stable.	Probably forms infinite array.	Dimeric protein is required for cooperativity of DNA binding [54].

Note: The dimeric molecule under consideration is highlighted using italics. For cases of interface asymmetry score 1 = 1, no interface asymmetry score 2 can be calculated. These are indicated as ##.

**Table 3:** Evolutionary aspects of functionally relevant homodimers exhibiting global asymmetry.

PDB code	Molecule	Number of homologs used in the sequence alignment	Is the interface(s) conserved?	Is any other surface patch well conserved?	Homologs with 3D structure from the same SCOP functional domain	Homologs with 3D structure from the same SCOP family
2c2l	*Carboxy terminus of Hsp70-interacting protein* (CHIP)+C-terminal peptide of Hsp70	22	Interface is more symmetric than rest of structure (2 parts – symmetric+asymmetric). Symmetric part is conserved. Asymmetric region is not conserved – very slight conservation.	Extended region from symmetric part is well conserved (probably complete interaction region with Hsp70)	Protein solved in complex with another protein by same group – only the interacting portion. Same results.	17 domains are present in the same superfamily. The asymmetric region is absent in almost all other cases.
1knz	*NSP3 homodimer*+RNA fragment	17*	Interface is reasonably well conserved.	Some residues on the edges are conserved – do not form a patch	None	None
1hwt	*Heme activator protein+*2 diff molecules of DNA	8	Interacting region is well conserved.	Region interacting with DNA is very well conserved.	3 entries of the same molecule – they are identical in structure to this molecule.	This family comprises of 6 domains. Some homo-oligomers are asymmetric and some symmetric. Some do not form homo-oligomers.
1f3m	*PAK1 autoregulatory domain*+PAK kinase domain	82	The interacting region is symmetric and reasonably conserved.	A small exposed patch is conserved.	None	None
1rlu	*Cell division protein ftsZ+*GSP	203	The two interacting surfaces overlap partially. One interface is poorly conserved, the other better conserved.	Extended region (region interacting with GSP) is well conserved. Another region on other surface fairly conserved.	Four structures – asymmetric binding.	Tubulin subunits – see another entry below
1lq1	*Stage 0 sporulation protein A+*2 diff molecules of DNA	64	Interacting surface is small and is moderately conserved on both sides. The interface regions are extended regions of the DNA-binding surface.	Region interacting with DNA is very well conserved.	One more structure – not bound to DNA	None
1mvo	*PhoP response regulator*	249	One interface is not conserved. Other interface is well conserved.	Another well conserved patch is present.	A structure from *E. coli* uses more conserved symmetric interface – but PISA says not stable!	25 domains in all – some have symmetric oligomers, some have asymmetric oligomers
1a6y	*Reverba orphan nuclear receptor+*2 diff molecules of DNA	248	Interacting surface is small and is moderately conserved on both sides. The interface regions are extended regions of the DNA-binding surface.	Region interacting with DNA is very well conserved.	None	12 domains in the family. Symmetric/asymmetric orientation is influenced by the direction of the repeats in the binding DNA. Predominantly, direct repeats cause asymmetric homodimerization and palindromic repeats cause symmetric homodimerization.
1kb2	*Vitamin D3 receptor+*2 diff molecules of DNA	248	Interacting surface is small and is moderately conserved on both sides. The interface regions are extended regions of the DNA-binding surface.	Region interacting with DNA is very well conserved.	None	Homolog of 1a6y. See entry above.
1jff	*Tubulin alpha and beta subunits*	49	The two patches involved in interaction are the most conserved surface patches.	No other conserved region.	Three structures – asymmetric binding	FtsZ is a homolog. See above.
1adv	*Adenovirus single-stranded DNA-binding protein*	14	Interface region is moderately conserved interspersed with unconserved parts.	Another patch containing a more conserved region present. It is not clear if this corresponds to the DNA binding site.	One more entry of the same protein with a different interface present.	None.

Note: Unless indicated by * all homologous sequences have been gathered from Uniref50 database. If very few homologues are identified then homologues identified from Uniref90 database (indicated by *) are used in the analysis. In a few PDB entries, several molecules are present. The dimeric molecule under consideration is highlighted using italics.

### Molecular aspects of asymmetry

**Figure 2:**
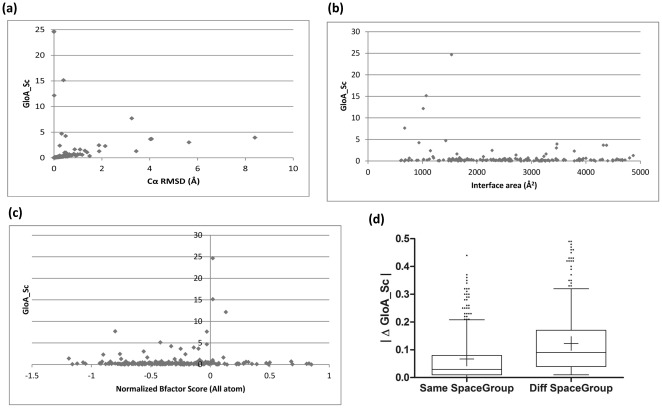
Molecular aspects of asymmetry. This figure shows the correlation of mathematical asymmetry captured by GloA_Sc with a) Cα-RMSD b) interface area c) normalized B-factors and d) crystal packing. Figures a,b,and c are scatter plots in which the molecular parameter being studied is shown along the X-axis and GloA_Sc along the Y-axis. In b), a subset of the overall graph cotnaining the majority of data is shown for clarity (with the maximum interface area being ∼25000 Å^2^). Figure d) is a box-plot representation of the absolute difference in GloA_Sc for the pairs of homodimers in each dataset. The horizontal bars present the 5 percentile, 25 percentile, 50 percentile, 75 percentile and 95 percentile values of each distribution and the mean value as ‘+’. Outliers are represented as dots.

Several structural and molecular parameters have been studied for globally asymmetric complexes in comparison with symmetric complexes and summarized in Table 1 and Figure 2.

#### Contributor to asymmetry: Subunit orientation versus Subunit conformational difference

Asymmetry in a homodimer can arise either due to conformational differences between the two subunits or differences in relative spatial orientation between the subunits or both. The contribution of conformational differences has been captured by considering the Cα-RMSD obtained after superposing the subunits using DALI [18]. Two protomers superimposed with a Cα-RMSD≤0.5 Å are considered to be conformationally similar in this analysis. From Table 1, we note that cases of conformationally similar protomeric subunits contributing to global asymmetry is highest (18.18%) for 0.4–0.6 bin. It should be noted that for majority number of dimers with GloA_Sc>1 the two protomers have substantial (>0.5 Å) RMSD. Therefore high structural difference between the two subunits in the dimer is a common scenario for examples with high structural asymmetry. Further, a scatter plot of Cα-RMSD versus GloA_Sc (Figure 2a) indicates that as Cα-RMSD increases, the GloA_Sc also increases. However, there also exist a few cases of conformationally similar subunits orienting very differently to result in a remarkably high GloA_Sc (>5). Overall, these results indicate that, in general, both conformational differences between subunits and difference in orientation between subunits contribute to global asymmetry.

#### Locally-contributed asymmetry versus Globally-contributed asymmetry

The question of whether global asymmetry arises due to asymmetry from a small set of residues or due to asymmetry spread over the entire molecule is analysed. This information is captured by considering homodimers where 10% of the residues contribute to top 25% of GloA_Sc (Table 1). It is observed that the highest prevalence is in the range 0.4–0.8 GloA_Sc (22.22%). However, there are no globally asymmetric complexes (GloA_Sc>3) where 10% of the residues contributes to top 25% of global asymmetry score, indicating that the global asymmetry is spread over the entire molecule. Even for cases of limited global asymmetry (GloA_Sc between 1–3), there are no cases of small number of residues contributing majorly to global asymmetry.

#### Interface area versus global asymmetry

A scatter plot of interface area versus GloA_Sc indicates that globally asymmetric homodimers usually tend to have smaller interface areas (<1800 Å^2^) (Figure 2b). On the contrary, symmetric homodimers can be formed using interfaces of different sizes (ranging up to 25000 Å^2^), although the majority have values <5000 Å^2^. However, it should be noted that the number of cases of globally asymmetric complexes are very small and, therefore, this result should be considered as a preliminary indication.

#### B-factors at interface region versus global asymmetry

A scatter plot of normalized B-factor at the interface versus GloA_Sc indicates that globally asymmetric homodimers usually tend to have moderately flexible interfaces (Figure 2c). Interestingly, the few examples with most pronounced asymmetry (GloA_Sc>5) correspond to low normalized B-factor. However, this result should also be considered as a preliminary indication as the number of globally asymmetric complexes is small. Symmetric homodimers can be formed using interfaces with different levels of flexibilities (Figure 2c).

#### Crystal packing versus global asymmetry

Further, the effect of crystal packing on global asymmetry was analysed. Two datasets were generated for this analysis (see Dataset S6 and Dataset S7). A box plot of the difference in GloA_Sc between the members of a pair is shown in Figure 2d for both the datasets. We see that there is a statistically significant difference between the distributions for the homodimers solved in same crystallographic space group compared to those solved in different crystallographic space groups (Mann-Whitney test; P-value<0.0001), indicating that crystal packing has an influence on global asymmetry. However, the mean and median values for the absolute difference in GloA_Sc for the ‘Same space group’ dataset (mean = 0.06, median = 0.03) and ‘Different space group’ dataset (mean = 0.12, median = 0.09) are negligible. In fact, the absolute difference is less than 0.2 for 95% of the cases in the ‘Same space group dataset’ and less than 0.3 for 95% of the cases in the ‘Different space group dataset’ (Figure 2d).

#### Residue composition versus asymmetry

To analyse if any of the 20 amino acid types have unusually high propensity to occur at an asymmetric interface, the propensities of the 20 amino acids to occur at the interface of symmetric homodimers vis-à-vis asymmetric homodimers were calculated (Table S1). The dataset of symmetric homodimers considered for this analysis consisted of all entries with GloA_Sc≤1 and the dataset of asymmetric homodimers consisted of all entries with GloA_Sc≥3. In order to perceive the signal on residue differences better the examples with GloA_Sc between 1 and 3 were not considered in this analysis. Results indicate that Phe, Tyr and Leu have higher propensity to occur in both symmetric as well as asymmetric interfaces (Table S1). Ile and Met show higher preference for symmetric interfaces whereas Gln shows higher preference for asymmetric interface (Table S1). However, it should be considered as a preliminary indication as the number of examples is small for the set of asymmetric homodimers (Table S1). Interestingly, the analysis partially agrees with the finding by Pednekar and Durani et. al that Gln, Asp, and Ala are symmetry breakers and Trp and His are symmetry makers [19]. However, other symmetry makers and breakers identified in their study are not picked up in this analysis.

### Homodimers with global asymmetry: Rare yet relevant

**Figure 3:**
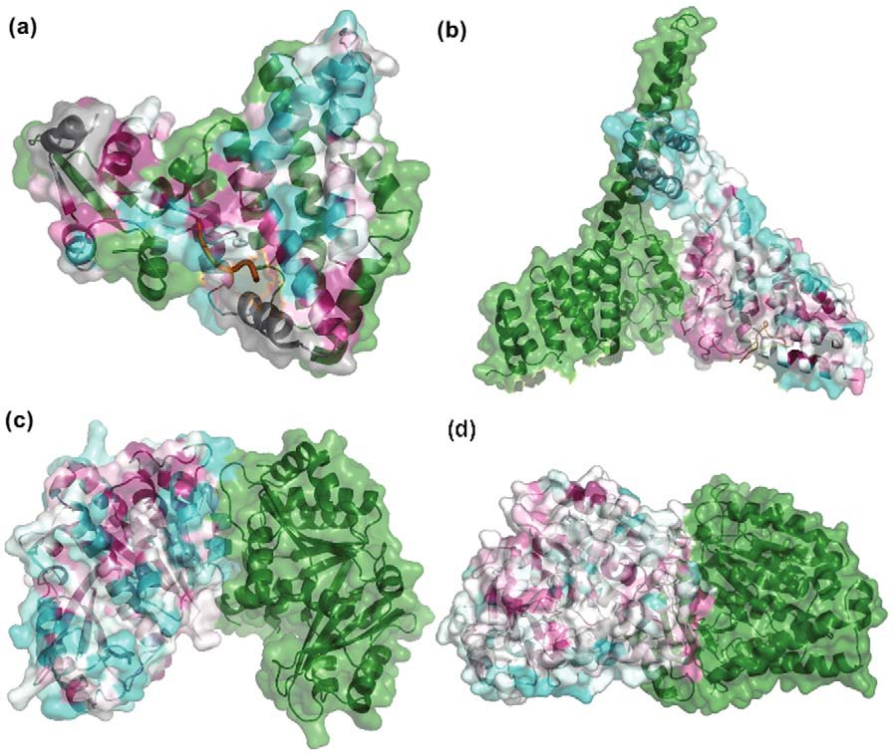
Homodimers exhibiting intrinsic global asymmetry. This figure shows examples of intrinsically asymmetric homodimers. a). NSP3 homodimer (GloA_Sc – 8.57; PDB - 1knz) b). Carboxy terminus of Hsp70-interacting protein (CHIP) (GloA_Sc – 8.29; PDB - 2c2l) c). Cell division protein FtsZ (GloA_Sc – 10.33; PDB – 1rlu) d). Tubulin α and β subunits (GloA_Sc – 23.09; PDB – 1jff). One of the chains of the dimer is shown as a green colored cartoon whereas the other chain provides a color-based representation of the conservation of every residue position, calculated using ConSurf *(refer Methods)*. In the chain colored based on ConSurf scores, highly conserved residues are colored magenta whereas poorly conserved residues are colored cyan and moderately conserved residues are shown in white. Any other ligand(s) if bound to the dimer is depicted in orange.

**Figure 4:**
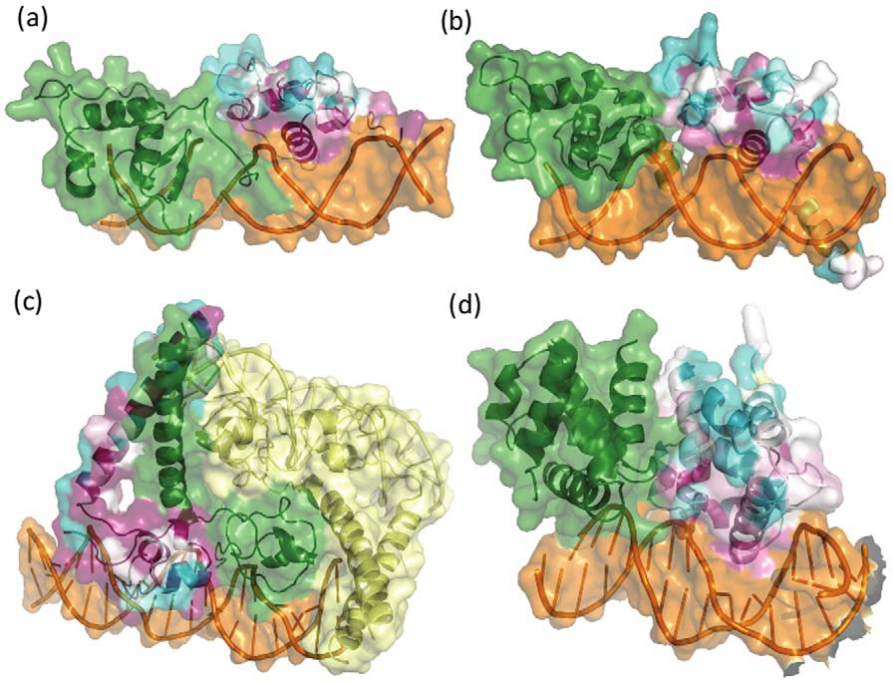
Homodimers exhibiting ligand-dependent global asymmetry. This figure shows examples of ligand-dependent asymmetric homodimers. a). Orphan nuclear receptor NR1D1 (GloA_Sc – 15.49; PDB – 1a6y) b). Vitamin D3 receptor (GloA_Sc – 17.47; PDB – 1kb2) c). Heme activator protein (GloA_Sc – 9.08; PDB – 1hwt) d). Stage 0 sporulation protein A (GloA_Sc – 11.79; PDB – 1lq1). One of the chains of the dimer is shown as a green colored cartoon whereas the other chain provides a color-based representation of the conservation of every residue position, calculated using ConSurf *(refer Methods)*. In the chain colored based on ConSurf scores, highly conserved residues are colored magenta whereas poorly conserved residues are colored cyan and moderately conserved residues are shown in white. Any other ligand(s) bound to the dimer is depicted as orange spheres. Other chains closely interacting in the asymmetric unit are colored yellow.

We studied a set of 11 globally asymmetric homodimers with known biological relevance gathered from literature and PiQSi. Details of the cases studied are listed in Tables 2 & 3. A picture of the asymmetric complex of the examples studied is shown in Figures 3, 4, S2.

The study reveals that asymmetry has been utilised by nature to perform several functions. A few complexes exhibit intrinsic asymmetry (Figure 3) whereas others exhibit ligand-dependent asymmetry (Figure 4). A few examples are discussed in depth.

#### Intrinsic asymmetry - A mechanism for 2∶1 binding

The non-structural protein 3 (NSP3) homodimer (Figure 3a) from rotavirus is essential for circularization of mRNA [20], which is a crucial process in viral translation. The N-terminal domain of this molecule has been shown to exist as a dimer in physiological conditions. Crystal structures solved by Deo et. al indicate that the asymmetric homodimerisation enables the generation of a single highly basic RNA-binding site [21]. The structure also validates experimental studies which reported the stoichiometry of NSP3∶RNA to be 2∶1 and also the necessity of dimerisation for strong RNA binding [22].

The C-terminal of Hsp70 interacting protein (CHIP) is a dimeric E3 ubiquitin ligase [23], [24] as well as a co-chaperone regulator [25]. It consists of an N-terminal tetratricopeptide repeat (TPR) domain and a C-terminal U-box domain connected via a helical region. The dimeric form is essential for function [26]. The dimer interface is constructed from two regions: a symmetric component contributed by the binding of U-box domain and an asymmetric component arising from the binding of helical hairpins (Figure 3b). The breaking of symmetry at the helical hairpins and differential placement of the C-termini in both protomers leads to variation in the location of the TPR domains with respect to their corresponding interacting partners i.e. U-box domains. This feature plays an important role in regulating the binding of ubiquitin conjugating enzyme Ubc13 with CHIP protein since their interaction occurs through the U-box domain. Since one of the sites is occupied by the TPR domain in one protomer, only one site is available for the ubiquitin conjugating enzyme Ubc13 to bind, leading to condition of half-of-sites binding [27]. In this manner, asymmetry provides an elegant means for coupling a single ubiquitin conjugation system to a dimeric chaperone (2∶1 binding) [28]. This system also illustrates how a small extent of interface asymmetry is translated into functional asymmetry at a global level (Figure S2a). p21 activated kinase 1 (PAK1) is another example of this type (Figure S2a), wherein the asymmetric dimer represents the auto-inhibited conformation of the molecule [29].

The bacterial protein FtsZ is essential for cell division [30]. Asymmetric association of two FtsZ protomers has been observed crystallographically by Leung et al. [31] (Figure 3c) and supported by mutation studies [32]. The large area buried upon complexation appears to lead to the formation of a stable dimer in solution. Fitting of this dimeric structure in the electron micrograph of spiral filaments of *Methanoccous janaschii* ftsZ provides a model that postulates a mechanism for Z-ring contraction [31]. Tubulins are the eukaryotic homologues of FtsZ. Unlike its bacterial counterpart, two non-identical (40% sequence identity) but structurally similar subunits of FtsZ, designated α-tubulin and β-tubulin, form the building blocks of microtubules [33]. α- and β- tubulin subunits associate in a head-to-tail orientation to form a longitudinal filament [34] (Figure 3d). Lateral associations of these longitudinal filaments leads to formation of a sheet-like structure which circularizes to form the hollow microtubules.

#### Ligand-dependent asymmetry - Asymmetry of the ligand directs asymmetric dimerisation of the protein

RevErb is a transcriptional repressor present in several species [35]. It belongs to the nuclear receptor superfamily, which consists of a large array of different transcription factors that bind to specific DNA sequences. The DNA-binding domains (DBD) of these receptors recognize specific DNA half-sites to carry out their function. Dimerisation of the DNA binding domains occurs only in the presence of the cognate DNA element [36]. The asymmetry in the dimer, solved by Zhao et al. (Figure 4a) is dictated by the head-to-tail arrangement of the cognate DNA repeats to which the receptor binds [35].

The vitamin D receptor (VDR) is a ligand-activated transcription factor, important in maintaining calcium homeostasis apart from regulating diverse biological functions [37], [38]. Similar to RevErb transcriptional repressor, VDR also dimerises only in the presence of its cognate DNA element. Asymmetric orientation (Figure 4b) has been shown by Shaffer and Gewirth to be induced by the head-to-tail arrangement of the direct repeats of the cognate DNA sequence [39].

Analysis of oligomeric structures of 10 other members of the nuclear receptor family reveals the following trends:

- Two of the members (PDB: 1dsz, 2nll) are stable as asymmetric dimers. The asymmetric orientation is due to the presence of direct DNA repeats of the cognate response element.
- Three of the members (PDB: 1hcq, 2han, 1r0n) are stable as symmetric/grossly symmetric dimers. The corresponding DNA response elements are either palindromic or pseudopalindromic; therefore, the receptors bind as symmetric dimers.
- Interestingly, an androgen receptor (PDB: 1r4i) from *Rattus norvegicus* binds as a symmetric dimer to direct DNA repeats.
- Two members (PDB: 1cit, 1lo1) bind the cognate DNA response element as monomers.

Heme activator protein 1 (HAP1) is a fungal transcription factor consisting of a Zn_2_Cys_6_ binuclear cluster domain. It regulates genes involved in oxidative phosphorylation and repair [40]–[42]. It adopts an asymmetric dimerisation interface (Figure 4c) to bind to two half-sites of its cognate DNA element arranged as direct repeats [43] [44]. The importance of the direction of the DNA repeats is shown by *in vitro* mutations that demonstrate that conversion of the direct repeat to palindromic inverted repeat results in drastic reduction of HAP1 binding [45].

Five other homologous proteins containing Zn_2_Cys_6_ domain and solved 3D structures are available. Two of them, PPR1 [46] and PUT3 [47], also show asymmetric DNA binding even though the DNA repeats are arranged in a symmetric fashion. Another homologue, GAL4, follows the expected arrangement of a symmetric homodimer binding to a palindromic repeat [48].

In essence, the analysis of these homodimers and the structures of the homologues indicate that the nature of dimer formed (symmetric/asymmetric) depends on the symmetry of the cognate DNA element. In most of the cases where the cognate DNA element is palindromic, the DBD dimers are symmetric. If the cognate DNA element is arranged as a direct DNA repeat, the DBD dimers are usually asymmetric tandem dimers.

### Prevention of infinite array formation

Asymmetry can, in principle, increase the chances of formation of infinite arrays or aggregation, which is known to cause disease states [49]. A structural and biochemical analysis of the examples studied reveals possible mechanisms for the prevention of infinite arrays.

#### Category 1: Overlapping interfaces

Asymmetric dimers can consist of interacting surfaces which are partially overlapping. Such an arrangement leads to usage of steric hindrance as a mechanism to prevent infinite array formation. The asymmetric dimers of CHIP, NSP3, FtsZ, heme activator protein, and PAK1 kinase seem to employ this mechanism. Partial asymmetry is probably favoured over complete asymmetry, characterized by exposed interacting patches, in these cases. The latter has high chances of formation of infinite arrays since all the dimers are characterized by large interface area, typically in the range of 3000–4000 Å^2^ and are stable even in the absence of any ligand (Table 2).

#### Category 2: Ligand-dependent structural change regulating polymerisation/depolymerisation

Tubulin and actin proteins associate in a head-to-fail fashion to generate polymeric filaments that are functionally relevant. However, the process is tightly regulated by the coupling of NTP/NDP bound states of the protein with several cellular factors. The phosphorylation state of the ligand (NTP/NDP) determines the structure of the dimeric building block of the protein facilitating polymerisation/depolymerisation, respectively. For example, α-tubulin is constitutively bound to GTP whereas β-tubulin can cycle between GTP and GDP. GTP binding to β-tubulin causes the αβ-heterodimer to adopt a straight head-to-tail assembled structure whereas the GDP-bound form of β-tubulin causes a bend in the dimer, which breaks the lateral associations leading to depolymerisation [50]. The intrinsic GTPase activity of tubulin ensures that depolymerisation occurs periodically contributing to the dynamic alteration of the microtubule structure. Several other external factors can also modulate the polymerisation/depolymerisation process [33]. The examples of tubulin and actin [51] illustrate that nature has used the mechanism of intrinsically and extrinsically regulated polymerisation/depolymerisation events to prevent infinite array formation for highly asymmetric homodimers exhibiting large asymmetry.

#### Category 3: Weaker interfaces coupled with ligand-induced dimerisation

Homodimers in this category contain interacting surfaces which are distinctly non-overlapping leading to exposure of binding patches. Although this characteristic is similar to tubulins and actins, polymerisation is not a requirement of function, even though it may help in cooperativity. In such cases, infinite array formation is a theoretical possibility which would be undesirable. However, it may not be physiologically relevant since most of the examples are characterized by smaller interacting surfaces (mostly <1000 Å^2^) with an exception of one of the cases burying an interface area of ∼3000 Å^2^. All dsDNA binding asymmetric dimers fall in this category – PhoP response regulator [52] (Figure S2b), orphan nuclear receptor, vitamin D3 receptor, and stage 0 sporulation protein [53] (Figure 4d). They are known to dimerise only in the presence of DNA. Several studies of cooperativity of DNA activation based on dimerisation of the protein are also demonstrated. These complexes appear to posses functional yet weak interfaces. Several factors appear to contribute to the weak interaction strength. Smaller interface area, poor conservation of one of the interacting surfaces and ligand-dependent asymmetric dimerisation appear to negate the formation of unwanted infinite arrays for such cases (Tables 2 & 3).

Another example is the case of a single stranded DNA binding protein from adenovirus (Figure S2c). Different crystal structures trap the C-terminal tails in different conformations (PDB: 1adv, 1adu). One of the structures (PDB: 1adv) indicates the formation of an infinite array caused by the interlocking of the C-terminal tail of one of the molecules with the base of another molecule [54]. The tail is essential for cooperative DNA binding, confirmed by deletion mutants [55], although it is not necessary for DNA binding. Dynamic light scattering experiments show that the C-terminal tail is flexible and can adopt several conformations. Therefore, flexibility of the tail controls the formation of infinite array in this case.

### Interface asymmetry in homodimers: Case of needle in a hay stack

Two measures for quantifying the extent of local asymmetry at the interface of a homodimer have been devised, based on the differences in the interacting residues and interactions between the two chains. Both scores are normalized with respect to number of residues and range from 0–1 with 0 indicating perfect symmetry and 1 indicating complete asymmetry. For cases where there are no common interacting residues between chain A and chain B (i.e. interface asymmetry score 1 equals 1), interface asymmetry score 2 cannot be calculated.

**Figure 5:**
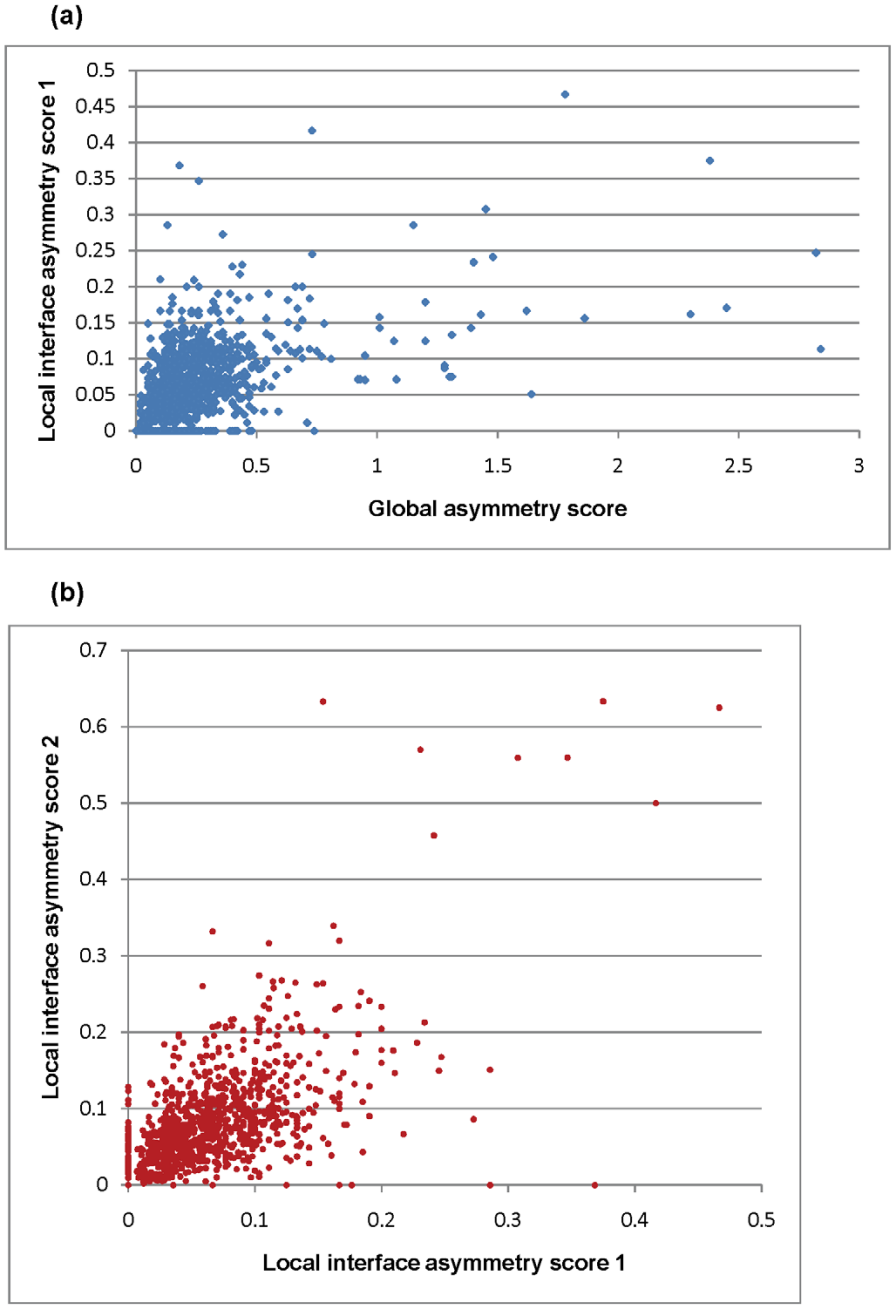
Interface asymmetry scores. This figure indicates the extent of interface asymmetry as computed using two scores for the set of symmetric homodimers. a). The correlation between global asymmetry score and interface asymmetry score 1 is depicted as a scatter plot. b). The correlation between local asymmetry score 1 and interface asymmetry score 2 is depicted as a scatter plot.

A study of the extent of asymmetry at the interface of 1149 symmetric homodimers was computed using interface asymmetry scores 1 and 2. To ensure that only symmetric homodimers were used, only cases with a global asymmetry score less than or equal to 3 was used. Statistics indicate that interface asymmetry is very rare in symmetric homodimers, with the number of unique interacting residues in any one of the protomers very rarely being greater than 20% (Figure 5). It is also seen that when global asymmetry scores are >1, the extent of asymmetry at the interface is slightly higher (Figure 5). The magnitude of asymmetry at the interface is usually very small and comparable to the changes caused due to variation in crystallisation conditions [56]. Therefore, ascertaining the biological relevance of interface asymmetry is like searching for a needle in a hay stack.

However, several cases of interface asymmetry implicated as relevant for performing the specific biological functions are known in literature. Some examples include half-of-site reactivity in case of caspase-9 caused by differential orientation of specific side chains [57], bending of tropomyosin molecules to enable binding with F-actin [58], and blood clot formation in fibrin [59]. Brown [60] studied >100 crystallographic complexes of symmetric homodimers exhibiting local asymmetry. He postulates the existence of sequence-dependent breaks in symmetry at homodimeric interfaces. A recent article delves deeply into the study of sequence-induced asymmetry leading to junction bends in the case of tropomyosin and other α-helical coiled coils [56]. A study by Pedneker and Durani further associates aromaticity with the ability to cause local asymmetry [19]. This analysis identifies aromatic amino acids (Tyr, Trp, Phe, His, Arg) as symmetry makers and aliphatic-polar and aliphatic-non polar groups as symmetry breakers.

**Figure 6:**
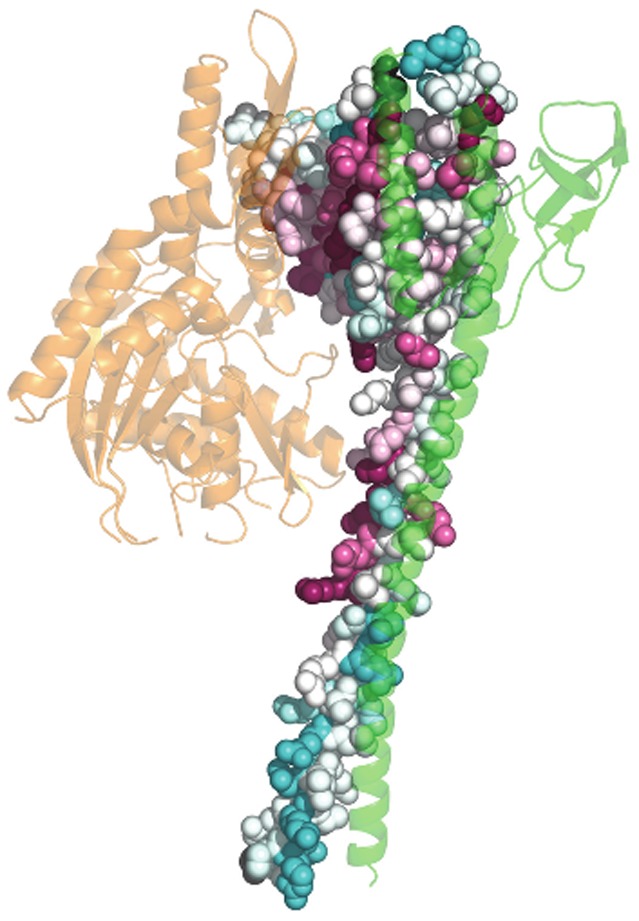
Case of local asymmetry in a symmetric homodimer. The figure shows the structure of the 2∶1 complex of GrpE with DnaK (PDB: 1dkg). One of the chains of the dimeric GrpE is shown as green colored cartoon whereas the other chain provides a color-based representation of the conservation of every residue position, calculated using ConSurf *(refer Methods)*. In the chain colored based on ConSurf scores, highly conserved residues are colored magenta whereas poorly conserved residues are colored cyan and moderately conserved residues are shown in white. DnaK is shown as orange cartoon.

An example of local asymmetry at the interface of a symmetric homodimer is shown in Figure 6 (GloA_Sc – 3.02; IntA_Sc1 – 0.25; IntA_Sc2 – 0.28), depicting a 2∶1 complex of GrpE with the ATPase domain of DnaK from *Escherichia coli* [61]. GrpE is a nucleotide exchange factor and DnaK a molecular chaperone of the Hsp70 family. Although the protomers of the dimeric GrpE show almost similar tertiary structures, one of the protomers has a kink in the interacting helical region. This kink causes the dimeric GrpE to bend to one side, which increases its interface area with the DnaK. The bend also enables the Phe-86 residue of GrpE to properly position Arg-183 of GrpE to form a hydrogen bond with Glu-28 of DnaK [61]. The local asymmetry in the structure provides an explanation for the biochemically observed 2∶1 binding between GrpE and DnaK [61]. Dimerisation has been proposed to be a necessity to stabilise the long helix of GrpE [61].

To ascertain whether interface asymmetry occurs due to structural differences at the interface between protomers or due to differential orientation between very similar protomers, interface asymmetry score 1 was correlated with the RMSD between the interacting residues of both the protomers for a given interface. We observe that the few cases with some extent of interface asymmetry (score 1≥0.2) occur in equivalent proportions due to both the reasons (Figure S3).

**Figure 7:**
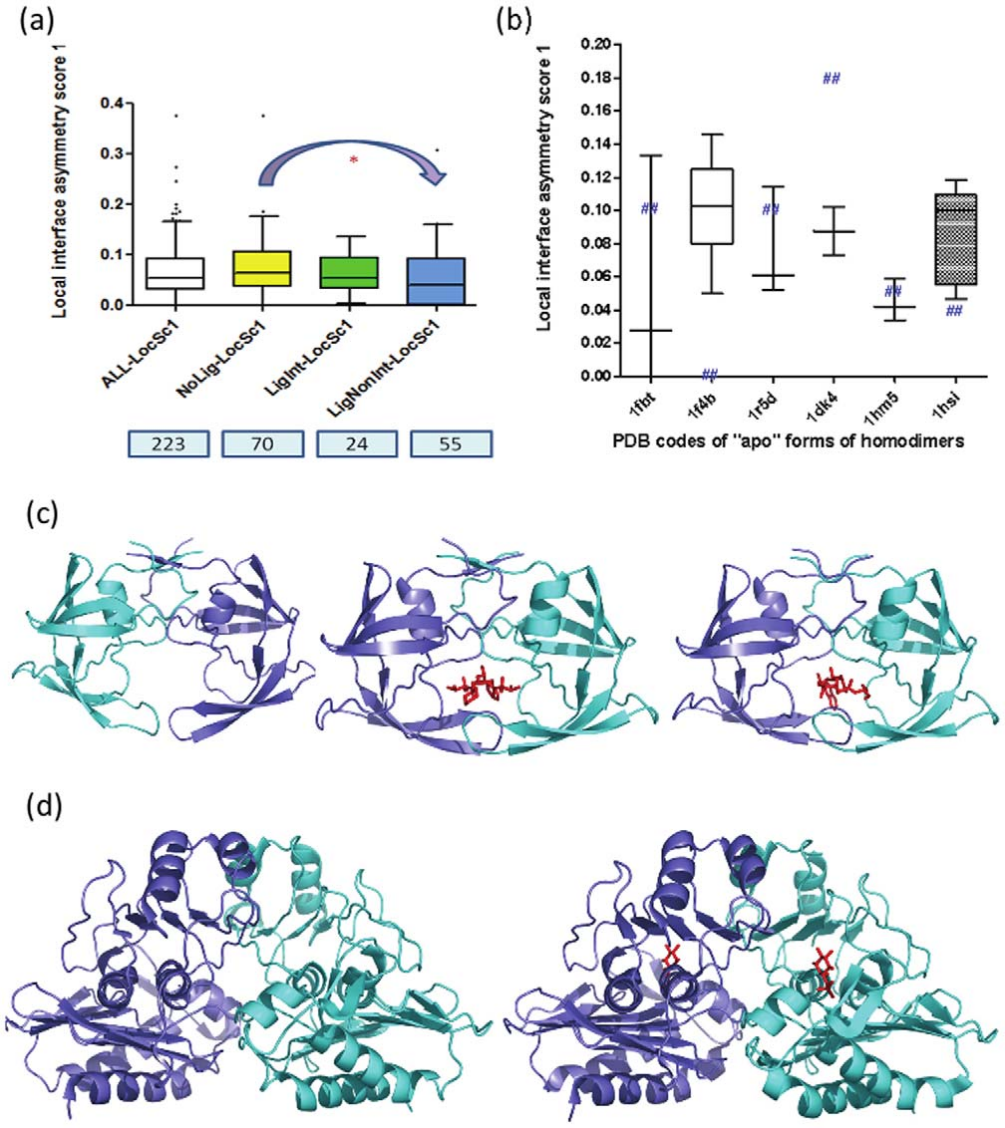
Ligand binding at the interface vs. interface asymmetry. This figure depicts the effect of ligand-binding on local asymmetry at the interface. a). The extent of local asymmetry score 1 (Y-axis) is plotted for various non-redundant datasets of homodimers (ALL - all kinds of symmetric homodimers, NoLig – Symmetric homodimers which are not bound to any biologically relevant ligands, LigInt – Symmetric homodimers which are bound to one/more ligands involved in ≥30% interaction with the dimer interface, LigNonInt - Symmetric homodimers which are bound to one/more ligands not involved in interaction with the dimer interface). The number of entries in every dataset is indicated in boxes below each dataset on the X-axis. b). The local interface asymmetry score 1 is plotted for 6 cases of ligand bound at the interface (holo) – ligand unbound (apo) pairs of symmetric homodimers. The scores for multiple different ligand-bound forms (holo) are indicated in the box plot whereas the score for the single “apo” member is indicated as ‘##’ in that box plot. The PDB codes of the “apo” forms are indicated on the X-axis. The case containing an asymmetric ligand at the interface is shown as a shaded box. c). This figure illustrates the structure of the HIV protease homodimer in the unliganded, and liganded (2∶1 complex) forms (for both symmetric and asymmetric ligands). The PDB codes for the shown structures are 1hsi, 1hii, and 1jld, respectively. d). This figure illustrates the structure of the inositol monophosphatase homodimer in the unliganded and liganded (2∶2 complex) forms. The PDB codes for the shown structures are 1dk4 and 1g0h, respectively.

Although the role of amino acid sequence in causing interface asymmetry has been studied, it is yet unknown whether ligand binding causes any variation. We studied this aspect using a dataset of homodimers containing biologically relevant ligands at the interface, collated from the MOAD database [62], [63]. The analysis indicates that small ligand binding does not cause any significant increase in interface asymmetry (Figure 7a). In fact, the asymmetry at the interface seems to be slightly reduced. Analysis of six specific cases of homodimers crystallised in their ligand-bound and free forms indicates that there is no systematic variation in interface asymmetry upon ligand binding (Figure 7b). This small dataset consists of varied types of ligands – symmetric single ligand bound to a dimer (Figure 7c), asymmetric single ligand bound to a dimer (Figure 7c), and small and large ligands bound in 2∶2 stoichiometry with the dimer (Figure 7d).

### Conclusion

Global asymmetry of homodimeric proteins has been utilised by nature to perform certain specialised functions, especially: the linking of a dimeric system with a monomeric system (half-of-sites reactivity) and the transmission of signals emanating from asymmetric DNA repeats. Study of the structural organization of homologues with known 3D structure reveals that there is no clear conservation of asymmetry. The function of the homologous protein appears to dictate the pattern of structural organization. For example, in the case of DNA-binding homodimers, the polarity of DNA repeats is a major factor in determining whether the homodimers assemble in a symmetric/asymmetric fashion. Interface asymmetry, wherever clearly shown to be of functional value, seems to exhibit sequence-dependency; with aromatic residues serving as symmetric makers and aliphatic residues serving as symmetry breaks [19]. However, binding of small ligands does not appear to have any influence on the extent of interface asymmetry.

The problem of infinite array formation, which is one of the reasons leading to the paucity of asymmetric homo-oligomers, appears to be addressed by nature in several ways. The usage of overlapping interfaces to cause steric hindrance and the usage of ligand-dependent structural changes or ligand-induced dimerisation are some of nature's ploys to prevent infinite array formation.

## Materials and Methods

### Dataset of biologically relevant homodimers

The dataset of biologically relevant homodimers was taken from PiQSi in 2009 [14], since it is manually curated (n = 3251). Only entities containing more than one chain in the asymmetric unit were considered to avoid cases of perfect crystallographic symmetry. The set was further pruned to include only those homodimers where both chains were 100% identical in terms of amino acid sequences, to avoid any bias arising due to the presence of extra residues. Finally, only those complexes which had a resolution equal to or better than 2.8 Å was considered for the analysis. Two versions of this dataset, redundant (n = 1149, see Dataset S1) and non-redundant (n = 223, see Dataset S2), were used for analysis. The non-redundant version was generated at 25% sequence identity using BLASTCLUST (http://www.csc.fi/english/research/sciences/bioscience/programs/blast/blastclust). Although stringent, the 25% sequence identity cut-off was chosen to ensure that no clear homologues (usually sequence identity >30%) are present in the non-redundant dataset. The redundant version contains both duplicate structures and structures of close homologues.

### Dataset of biologically relevant asymmetric homodimers

Entries of homodimers in PiQSi are broadly categorized as symmetric or non-symmetric (termed ‘asymmetric’ in our analysis). The classification is based on a procedure involving the rotation of both subunits (by 360/N angles – where ‘N’ is the number of subunits in the complex) about a set of 600 axes passing through the centre of mass of the structure [10]. If the average Euclidian distance after all rotations is >7 Å for all axes, then the structure is considered to be non-symmetric. From the redundant dataset of homodimers generated, entries with a global asymmetry score ≥7 (n = 23) were considered as a starting set of asymmetric homodimers. This set was also augmented by entries culled manually from literature (n = 6). Thorough literature analysis of these complexes (23+6) yielded a selection of 11 homodimers with pronounced asymmetry with clear functional relevance elucidated from experiments. For these 11 cases, homologues of known 3D structure, identified as members belonging to the same SCOP [64] family, were obtained for further analysis.

### Dataset of ‘small-ligand bound’ and ‘ligand unbound’ symmetric homodimers

To analyse whether ‘small-ligand’ binding causes any systematic variation resulting in interface asymmetry, the following test and control non-redundant (at 25% sequence identity) datasets of homodimers were generated. For all sets, only entries with 2 chains in asymmetric unit and resolution ≤2.8 Å were considered.

#### Ligand-Unbound dataset (NoLig)

The dataset of homodimers not bound to any biologically relevant ligand was culled from RCSB [65] (http://www.rcsb.org) by using the appropriate search terms in their Advanced Search page. This forms the main Control dataset (n = 70, see Dataset S3).

#### Ligand-bound-at-interface dataset (LigInt)

The initial dataset of homodimers bound to biologically relevant small ligands was taken from the MOAD database [62], [63] (http://www.bindingmoad.org). It was further pruned by identifying only those entries in which at least 30% of the ligand interacting surface was involved in binding with residues lining the dimeric interface. This forms the Test dataset (n = 24, see Dataset S4).

#### Ligand-notbound-at-interface dataset (LigNonInt)

The initial dataset of homodimers bound to biologically relevant small ligands was taken from the MOAD database [62], [63] (http://www.bindingmoad.org). It was further pruned by identifying only those entries where the ligand was not involved in interaction with any of the residues lining the dimeric interface. This forms the subsidiary Control dataset to distinguish the variation in interface asymmetry, if any, caused due to ligand-binding at the interface vs. away from the interface (n = 55, see Dataset S5).

#### Overall dataset (ALL)

The pruned dataset of non-redundant entries taken from PiQSi (n = 223, see Dataset S2).

### Datasets of identical homodimer pairs solved in ‘same’ and ‘different’ crystallographic space groups

#### ‘Same space group’ dataset

From the dataset of 1149 homodimeric complexes considered for analysis, pairs of homodimers solved in the same crystallographic space group were extracted. Further, two pairs were randomly selected for each PDB code to ensure that there is no bias due to over-representation of some PDBs. The final dataset consists of 743 homodimeric pairs (n = 743, see Dataset S6).

#### ‘Different space group’ dataset

From the dataset of 1149 homodimeric complexes considered for analysis, pairs of homodimers solved in different crystallographic space group were extracted. Further, three pairs were randomly selected for each PDB code to ensure that there is no bias due to over-representation of some PDBs. The final dataset consists of 516 homodimeric pairs (n = 516, see Dataset S7).

### Method for calculation of extent of global asymmetry in a homodimer

A measure of global asymmetry of a homodimeric complex designed by Andre et. al [9] based on Cα-Cα distances (Figure 1a & 1b) was used. Consider a homodimeric complex containing 100% identical chains A and B in terms of amino acid sequence. For a given residue in chain A, its Cα distance with all other residues in chain B has been calculated. A reciprocal calculation was done with the same residue from chain B with all other residues in chain A. The measure of absolute differences between the two distances has been calculated and normalized by the number of distance calculations performed. These steps are repeated for all the residues in both the chains to arrive at the asymmetry score using the following formula:
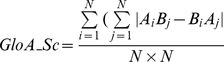
where i, j are residue numbers ranging from 1 to N and N is the total number of residues in a chain. A and B represent the two chains in the homodimer. The minimum value that can be obtained is 0, indicating perfect symmetry (mathematical symmetry). There is no limit to the maximum value that can be obtained, since it can vary based on the size and extent of asymmetry of the complex.

### Proposition of a simple method for calculation of extent of local asymmetry at the interface of a homodimer

A measure of local asymmetry at the interface of homodimeric complexes has been designed based on the extent of unique interacting residues and interactions present between the two chains. The set of interacting residues in a complex is determined using a distance cutoff calculation which considers two residues from chain A and chain B to be interacting if at least one pair of atoms from the two residues characterized by a distance (between them) less than the sum of the van der Waals radii of the corresponding atoms +0.5 Å [66]. The van der Waals radii were taken from the literature [67].

Given a set of interacting residues between chain A and chain B, two local interface asymmetry scores are calculated:

#### Interface asymmetry score 1

This score quantifies the extent of asymmetry at the interface on the basis of the fraction of unique interacting residues in both chains (Figure 1c). The formula used for the calculation is
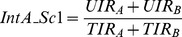
where

UIR_A_ – number of unique interacting residues in chain A

UIR_B_ – number of unique interacting residues in chain B

TIR_A_ – total number of interacting residues in chain A

TIR_B_ – total number of interacting residues in chain B

This score can range from 0–1 with 0 indicating perfect interface symmetry and 1 indicating complete interface asymmetry, ie. the situation wherein none of the interface residues between the two chains are common. The latter situation would be observed in the case of a globally asymmetric complex which uses different surfaces for the interaction.

#### Interface asymmetry score 2

For each common interacting residue determined in interface asymmetry score 1, this score quantifies the extent of asymmetry on the basis of the fraction of unique interactions in each chain (Figure 1d). The final score is summed over all the common interacting residues. The formula used for the calculation is
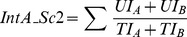
where

UI_A_ – number of unique interactions for a common interacting residue in chain A

UI_B_ – number of unique interactions for a common interacting residue in chain B

TI_A_ – total number of interactions for a common interacting residue in chain A

TI_B_ – total number of interactions for a common interacting residue in chain B

The calculation of the interface asymmetry score 2 is similar to the calculation of interface asymmetry score 1. The difference arises only in the data used for the calculation. In case of score 1, ‘interacting residues’ are considered whereas in score 2, ‘interactions of common interacting residues’ are considered. This score can range from 0–1 with 0 indicating perfect symmetry for the common interacting residue and 1 indicating complete asymmetry for the common interacting residue. In the special case that interface asymmetry score 1 is 1, interface asymmetry score 2 cannot be calculated since there are no common interacting residues.

### Calculation of structural attributes of homodimeric complexes

#### Interface area

The interface area of a homodimer (AB) has been calculated using solvent accessible surface area computed using NACCESS program [68]. A probe radius of 1.4 Å has been used. The interface area for the homodimer AB is given by
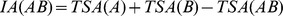
where

IA = Interface area

TSA = Total surface area

#### Stability of the complex

The stability of a complex has been evaluated using PISA [69], which uses thermodynamic principles to evaluate the probability of the crystallised complex being stable.

#### Conservation of residues at the interface patch

Conservation of residues at the interacting surface has been analysed using ConSurf [70], [71]. This method uses a multiple sequence alignment of homologous proteins and calculates the conservation of residues at each site using an empirical Bayesian method weighted using the phylogenetic distance between sequences. The set of homologues were identified using PSI-BLAST [72] against UniRef50 and UniRef90 databases [73]. Only sequences having E-value better than 10^−5^ along with sequence identity ≥30% and query coverage ≥70% have been considered as clear homologues. The multiple sequence alignment of these sequences has been generated using ClustalW [74] for submission to the ConSurf server [75].

#### Flexibility at the interface

To ascertain the flexibility/rigidity of the interface residues in a homodimer, the normalized all-atom B-factor for every interface residue was calculated [76]. The average value was taken as an indicator of the extent of flexibility at the interface.

## Supporting Information

Figure S1 **A panel of homodimers with increasing global asymmetry.** This figure shows the structure of several homodimers and their associated global asymmetry scores, in ascending order. The two chains are colored orange and cyan. The N-terminal region of each chain is colored dark blue to provide a visual picture of the extent of asymmetry in the dimer. a). Bovine pancreatic ribonuclease A (GloA_Sc – 2.84) b). High potential iron protein structure (GloA_Sc – 4.25) c). Probable ATP-dependent RNA helicase (GloA_Sc – 4.70) d). Epidermal growth factor-like domain from human factor IX (GloA_Sc – 7.63) e). Alkaline phosphatase synthesis transcriptional regulatory protein PhoP (GloA_Sc – 12.16) f). Adenovirus single-stranded DNA-binding protein (GloA_Sc – 23.42)
(TIF)

Figure S2 **Other cases of global asymmetry considered in our study.** This figure shows the structures of other globally asymmetric homodimers considered in the study. a). PAK1 autoregulatory domain complexed with kinase domain (GloA_Sc – 9.63; PDB – 1f3m) b). PhoP response regulator (GloA_Sc – 12.16; PDB – 1mvo) c). Adenovirus single-stranded DNA binding protein (GloA_Sc – 23.42; PDB – 1adv). One of the chains of the dimer is shown as a green colored cartoon whereas the other chain provides a color-based representation of the conservation of every residue position, calculated using ConSurf *(refer Methods)*. In the chain colored based on ConSurf scores, highly conserved residues are colored magenta whereas poorly conserved residues are colored cyan and moderately conserved residues are shown in white.
(TIF)

Figure S3 **Local RMSD at interface vs. interface asymmetry score 1.** This figure explores the correlation of ‘structural changes between the interface residues of the two protomers in the homodimer’ with the corresponding ‘interface asymmetry score 1’ for one of the protomers in the dataset of 1139 homodimers. a). A scatter plot between “Interface asymmetry score 1” on the X-axis and “Cα-RMSD between interacting residues of protomers” on the Y-axis is shown. b). A scatter plot between “Interface asymmetry score 1” on the X-axis and “Sidechain-RMSD between interacting residues of protomers” on the Y-axis is shown.
(TIFF)

Table S1 **suPropensity for 20 amino acid types to occur at the interfaces of symmetric and asymmetric homodimers.** The table provides information about the number of data points used for propensity calculation along with the propensity values.
(DOC)

Dataset S1 **List of PDB codes corresponding to redundant dataset of homodimers.** The list of PDB codes corresponding to the redundant dataset of homodimers used in this study is listed.
(DOC)

Dataset S2 **List of PDB codes corresponding to non-redundant dataset of homodimers.** The list of PDB codes corresponding to the non-redundant dataset of homodimers used in this study is listed.
(DOC)

Dataset S3 **List of PDB codes corresponding to non-redundant dataset of homodimers complexed without any ligands.** The list of PDB codes corresponding to the non-redundant dataset of homodimers not complexed with any ligands used in this study is listed.
(DOC)

Dataset S4 **List of PDB codes corresponding to non-redundant dataset of homodimers complexed with ligands bound at the interface.** The list of PDB codes corresponding to the non-redundant dataset of homodimers complexed with ligands interacting with the dimer interface used in this study is listed.
(DOC)

Dataset S5 **List of PDB codes corresponding to non-redundant dataset of homodimers complexed with ligands bound away from the interface.** The list of PDB codes corresponding to the non-redundant dataset of homodimers complexed with ligands bound at regions away from the dimer interface used in this study is listed.
(DOC)

Dataset S6 **List of PDB codes corresponding to the pairs of identical homodimers solved in the same crystallographic space group.** List of PDB codes corresponding to the pairs of identical homodimeric proteins solved in the same crystallographic space group is listed along with details of the space group and GloA_Sc.
(DOC)

Dataset S7 **List of PDB codes corresponding to the pairs of identical homodimers solved in different crystallographic space groups.** List of PDB codes corresponding to the pairs of identical homodimeric proteins solved in different crystallographic space group is listed along with details of the space groups and GloA_Sc.
(DOC)
