# Supplementary figures and images for: Extent of Structural Asymmetry in Homodimeric Proteins: Prevalence and Relevance
Source: PLoS One. 2012 May 22;7(5):e36688. doi: 10.1371/journal.pone.0036688 (PMC3358323; doi:10.1371/journal.pone.0036688)

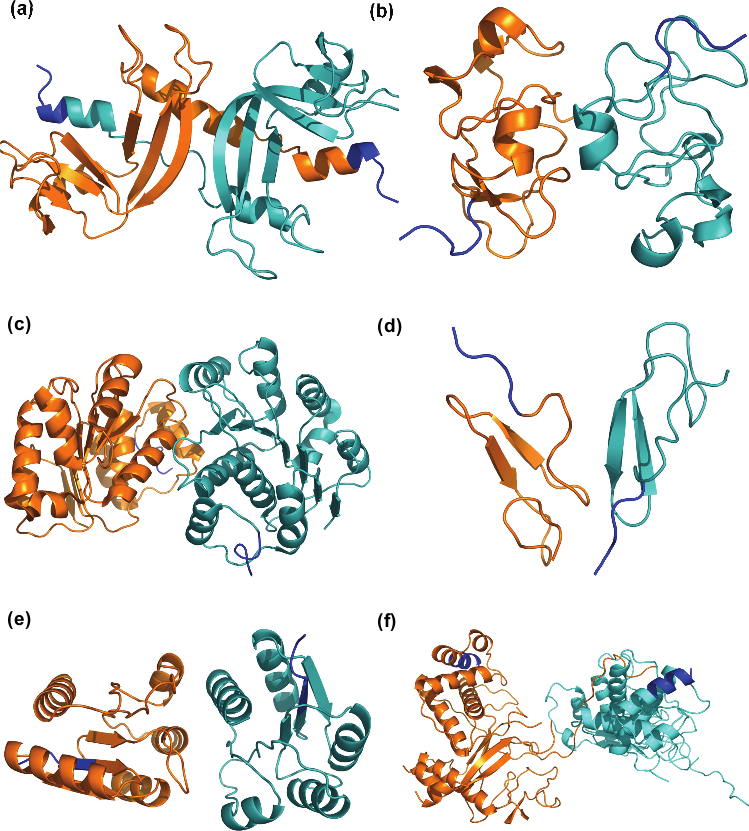

Supplement: Figure S1 — A panel of homodimers with increasing global asymmetry. This figure shows the structure of several homodimers and their associated global asymmetry scores, in ascending order. The two chains are colored orange and cyan. The N-terminal region of each chain is colored dark blue to provide a visual picture of the extent of asymmetry in the dimer. a). Bovine pancreatic ribonuclease A (GloA_Sc – 2.84) b). High potential iron protein structure (GloA_Sc – 4.25) c). Probable ATP-dependent RNA helicase (GloA_Sc – 4.70) d). Epidermal growth factor-like domain from human factor IX (GloA_Sc – 7.63) e). Alkaline phosphatase synthesis transcriptional regulatory protein PhoP (GloA_Sc – 12.16) f). Adenovirus single-stranded DNA-binding protein (GloA_Sc – 23.42) (TIF) [file pone.0036688.s001.tif]

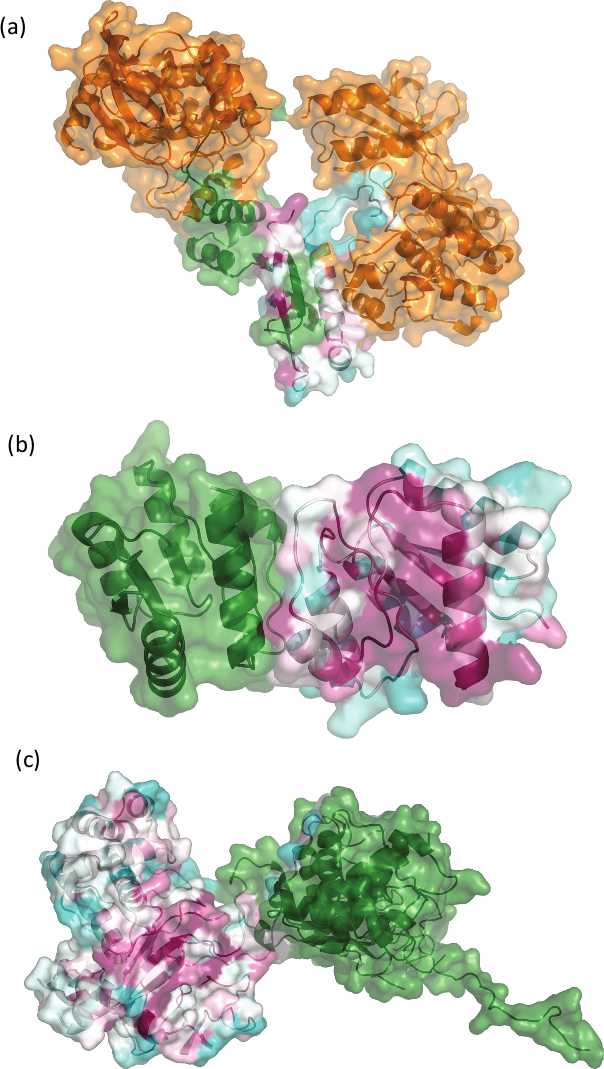

Supplement: Figure S2 — Other cases of global asymmetry considered in our study. This figure shows the structures of other globally asymmetric homodimers considered in the study. a). PAK1 autoregulatory domain complexed with kinase domain (GloA_Sc – 9.63; PDB – 1f3m) b). PhoP response regulator (GloA_Sc – 12.16; PDB – 1mvo) c). Adenovirus single-stranded DNA binding protein (GloA_Sc – 23.42; PDB – 1adv). One of the chains of the dimer is shown as a green colored cartoon whereas the other chain provides a color-based representation of the conservation of every residue position, calculated using ConSurf (refer Methods). In the chain colored based on ConSurf scores, highly conserved residues are colored magenta whereas poorly conserved residues are colored cyan and moderately conserved residues are shown in white. (TIF) [file pone.0036688.s002.tif]

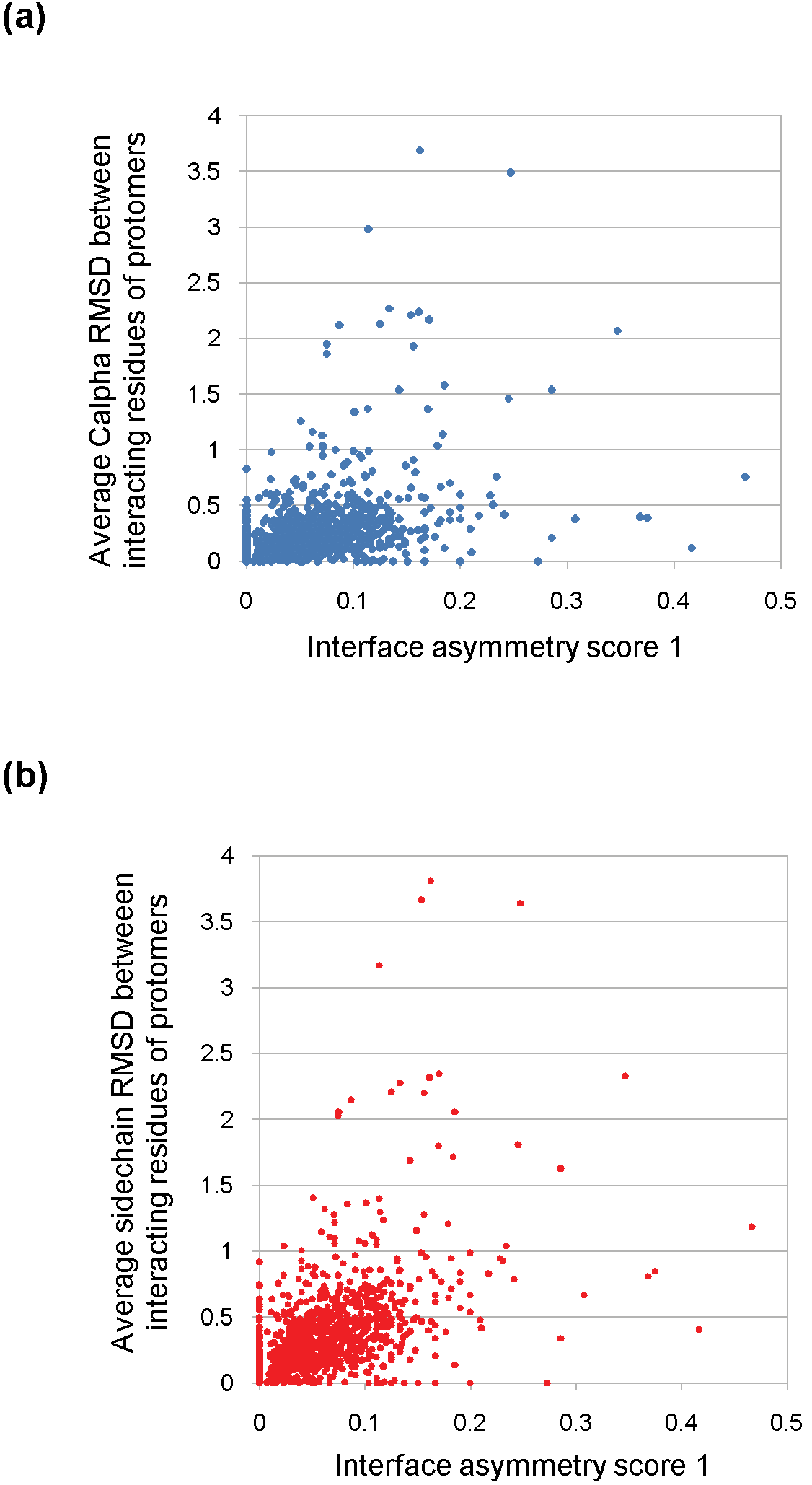

Supplement: Figure S3 — Local RMSD at interface vs. interface asymmetry score 1. This figure explores the correlation of ‘structural changes between the interface residues of the two protomers in the homodimer’ with the corresponding ‘interface asymmetry score 1’ for one of the protomers in the dataset of 1139 homodimers. a). A scatter plot between “Interface asymmetry score 1” on the X-axis and “Cα-RMSD between interacting residues of protomers” on the Y-axis is shown. b). A scatter plot between “Interface asymmetry score 1” on the X-axis and “Sidechain-RMSD between interacting residues of protomers” on the Y-axis is shown. (TIFF) [file pone.0036688.s003.tiff]
