# Supplementary material for: Extent of Structural Asymmetry in Homodimeric Proteins: Prevalence and Relevance
Source: PLoS One. 2012 May 22;7(5):e36688. doi: 10.1371/journal.pone.0036688 (PMC3358323; doi:10.1371/journal.pone.0036688)
Supplement: Table S1 — suPropensity for 20 amino acid types to occur at the interfaces of symmetric and asymmetric homodimers. The table provides information about the number of data points used for propensity calculation along with the propensity values. (DOC) [file pone.0036688.s004.doc]

**Table S1: Propensity for amino acids to occur at the interfaces of symmetric and asymmetric homodimers.**

| **Amino acid** | **Number of interface residues for homodimers with GloA_Sc≤1** | **Number of interface residues for homodimers with GloA_Sc≥3** | **Number of surface residues (≥10% RSA) for all homodimers** | **Propensity to form interface residues for homodimers with GloA_Sc≤1** | | | **Propensity to form interface residues for homodimers with GloA_Sc≥3** | |  |
| --- | --- | --- | --- | --- | --- | --- | --- | --- | --- |
| A | 861 | 26 | 24366 | |  | 0.98 | | 1.38 | |
| C | 175 | 1 | 3265 | |  | 1.48 | | 0.39 | |
| D | 664 | 30 | 28913 | |  | 0.63 | | 1.34 | |
| E | 870 | 13 | 35235 | |  | 0.68 | | 0.47 | |
| F | 835 | 20 | 10031 | |  | **2.31** | | **2.58** | |
| G | 768 | 16 | 31535 | |  | 0.67 | | 0.65 | |
| H | 421 | 3 | 8608 | |  | 1.35 | | 0.45 | |
| I | 775 | 11 | 12534 | |  | **1.71** | | 1.13 | |
| K | 653 | 21 | 37093 | |  | 0.48 | | 0.73 | |
| L | 1249 | 36 | 21278 | |  | **1.63** | | **2.19** | |
| M | 430 | 3 | 5559 | |  | **2.14** | | 0.70 | |
| N | 594 | 3 | 20595 | |  | 0.80 | | 0.18 | |
| P | 674 | 12 | 21427 | |  | 0.87 | | 0.72 | |
| Q | 581 | 27 | 19044 | |  | 0.84 | | **1.84** | |
| R | 910 | 18 | 23739 | |  | 1.06 | | 0.98 | |
| S | 706 | 17 | 23392 | |  | 0.83 | | 0.94 | |
| T | 719 | 8 | 22655 | |  | 0.88 | | 0.46 | |
| V | 837 | 7 | 15721 | |  | 1.47 | | 0.57 | |
| W | 272 | 4 | 4469 | |  | **1.69** | | 1.16 | |
| Y | 691 | 17 | 10673 | |  | **1.79** | | **2.06** | |

Values with > 1.00 have high increased preference to occur in a particular category. The bold underlined values are greater than 1.50 indicating high propensity values. The underlined values have a propensity value between 1.25 and 1.50.
