# Supplementary material for: Extent of Structural Asymmetry in Homodimeric Proteins: Prevalence and Relevance
Source: PLoS One. 2012 May 22;7(5):e36688. doi: 10.1371/journal.pone.0036688 (PMC3358323; doi:10.1371/journal.pone.0036688)
Supplement: Dataset S1 — List of PDB codes corresponding to redundant dataset of homodimers. The list of PDB codes corresponding to the redundant dataset of homodimers used in this study is listed. (DOC) [file pone.0036688.s005.doc]

**Dataset S1: List of PDB codes corresponding to r**edundant dataset of homodimers

| 117e,11bg,11gs,14gs,16gs,18gs,19gs,1a0f,1a2w,1a4o_1,1a4o_2,1a4u,1a64,1a71,1a78,1a8j,1a9m,1aaq,1aar,1aat,1ac6,1ad3,1adb,1adc,1adj_1,1adj_2,1ady_1,1ady_2,1ag1,1ags,1ahe,1ahf,1ahx,1ahy,1aia,1aib,1aic,1aid,1aiq,1aj5_1,1aj5_2,1ajr,1ajv,1ajx,1aka,1alv,1alw,1an5,1aoj,1aoz,1arg,1arh,1ari,1asl,1asm,1asn,1aso,1asp,1asq,1avb,1awb,1axa,1axe,1axg_1,1axg_2,1axw,1azv,1b02,1b0w_1,1b0w_2,1b0w_3,1b14,1b15,1b48,1b49,1b57,1b5o,1b5p,1b6d,1b6j,1b6k,1b6l,1b6m,1b6p,1b6s_2,1b78,1b8c,1b8z,1bbh,1bdl,1bdq,1bdr,1biu_2,1bjf,1bjm,1bjw,1bk5,1bkg_1,1bkg_2,1bko_1,1bko_2,1bmd,1bq1,1bqa,1bqd,1brm_2,1bsf,1bsr,1btm,1bto_1,1bto_2,1bv7,1bv9,1bwa,1bwb,1bww,1bye_1,1bye_2,1byf,1byk,1c1j_1,1c1j_2,1c50,1c6o,1c6x,1c6y,1c6z,1c70,1c72_1,1c72_2,1c7z,1c80,1cb4,1cbj,1cdc,1cdd,1cdo,1chm,1chw,1ci7,1cku,1cob,1coz,1cqs,1d1g,1d4a_1,1d4a_2,1d4h,1d4i,1d4j,1d4s,1d4y,1d5n_1,1d5n_2,1d6s,1daa,1dap,1dbn,1dbq,1dcl,1dd3_1,1ddu,1deh,1dif,1dk4,1dmp,1dna,1dor,1dos,1dpg,1dqn,1dqp,1dqr,1dty,1dug,1dvi,1dxo_1,1dxo_2,1dzt,1e2q,1e4n,1e56_1,1e7n,1e8i,1ebl,1ebw,1eby,1ebz,1ec0,1ec1,1ec2,1ec3,1ec5_1,1ec5_2,1ecz,1edh,1edm,1eeq,1eix_1,1ek3,1ekf,1ekm_1,1ekm_2,1ekp,1ekv,1elq,1elu,1em6,1en4_1,1en5_1,1en5_2,1eog,1eoh_1,1eoh_2,1eoh_3,1eoh_4,1eq9,1eqg,1eqh,1eqt,1ewz_1,1exv,1eyz,1ez1,1f1c,1f1g_1,1f1g_2,1f1g_3,1f28_1,1f28_2,1f3a,1f3b,1f4d,1f4o,1f89,1fbt,1fc4,1fe4,1fjh,1fof,1fro_1,1fro_2,1ftl,1fwm,1fx9,1fxf,1fxr,1fyu,1g0h,1g0i,1g0z,1g1a_1,1g1a_2,1g2k,1g2w,1g35,1g51,1g8l,1g8r,1g98,1gam,1gan,1gck,1gd9,1gde,1gdh,1gfl,1gg5_1,1gg5_2,1gk4_1,1gl3,1glp,1glq,1gmg_1,1gmg_2,1gnm,1gnn,1gno,1gnw,1gpe,1gq1,1gq9,1gqa,1gqi,1gqj,1gqk,1gsd_1,1gsd_2,1gse,1gsf_1,1gsf_2,1gsu,1gsy,1gtv_1,1gtv_2,1gu7,1guf,1guh_1,1guh_2,1gv3,1h0k,1h0x,1h18,1h1y,1h49,1h65_1,1h66_1,1h66_2,1h69_1,1h69_2,1h6j,1h7x_2,1h8x,1h8y,1h8z,1h91,1h9r,1ha4,1hbi,1hbv,1hdx,1hdy,1hdz,1hih,1hii,1hj5,1hld,1hm5,1hn9,1hos,1hox,1hpo,1hps,1hpv,1hpx,1hsg,1hsh_1,1hsh_2,1hsi,1hso,1hsz,1ht0,1ht5,1ht8,1ht9,1htb,1htf,1htg,1hti,1huj,1huk,1hvh,1hvi,1hvj,1hvk,1hvl,1hvr,1hvs,1hvy_1,1hvy_2,1hw1,1hwr,1hxb,1hxw,1hzj,1hzp,1i00,1i07,1i08_1,1i08_2,1i0h,1i1c,1i45,1iax,1ib6_1,1ib6_2,1ida,1idb,1ie3_1,1ie3_2,1iig,1iih,1ima,1imb,1ime,1ipe,1ipf,1iqx,1iqy,1iri_1,1iri_2,1isa,1isb,1isc,1iu7,1iug,1ivl,1ivu,1ivv,1ivw,1ivx,1iz9,1izh,1izi,1izy,1j2e,1j32,1j49,1j4a_1,1jb2,1jb4,1jb5,1jcz,1jeh,1jg0,1jhz,1jiq_1,1jiq_2,1jld,1jlh_1,1jlh_2,1jlw,1jm0_1,1jm0_2,1jm0_3,1jmb_1,1jmb_2,1jqv,1jqx,1jrb,1jrc,1jtq,1jtu,1ju9,1jue,1juo,1jut,1jwn_1,1jwn_2,1jxh,1jxi,1jxn_1,1jys,1jzk_1,1jzk_2,1jzm,1k3y,1k41,1k66,1k8c_2,1k94,1k9k,1kbn,1kbo_1,1kbo_2,1kce,1keu,1kew,1kji,1kl2,1knq,1ko5,1kof,1kp0,1ks2,1ksi,1kso,1kta,1kv5,1kzi,1kzj_1,1kzj_2,1l0w,1l5b,1l5v,1l5w,1l6i,1l8s,1lbk,1lbv,1lbw,1lbx,1lby,1lbz,1ldy_1,1ldy_2,1lgv,1lhz,1lil,1lkz,1llf,1lpf,1lq9,1lt1_1,1lt1_2,1lt1_3,1lt1_4,1lwh,1lwj,1lyn,1lzo_1,1lzo_2,1m0s,1m0u,1m38,1m5b_2,1m5e_2,1m5f_2,1m5j,1m5m,1m6h,1m6j,1m6w,1m7o,1m7p,1m9m,1m9q,1m9r,1ma0,1mb4,1mc5,1md3,1md4,1mer,1mes,1met,1meu,1mft,1mg0_1,1mg0_2,1mg5,1mgo,1mgv,1mi3_1,1mi3_2,1mk4,1mka,1mkb,1ml6,1mm7_1,1mm7_2,1mmm,1mo9,1moe,1mok_1,1mok_2,1mp0,1mqd_1,1mqd_2,1mqd_3,1mqd_4,1mqg_1,1mr8,1mrw,1mrx,1ms7_1,1ms7_2,1msb,1msm,1msn,1mtb,1mtc,1mui,1mvo,1mxu_1,1mxu_2,1mxv_1,1mxv_2,1mxw_1,1mxw_2,1mxx_1,1mxx_2,1mxy_1,1mxy_2,1mxz_1,1mxz_2,1my0_1,1my0_2,1my1_1,1my1_2,1my2_1,1my2_2,1my3_1,1my3_2,1my4_1,1my4_2,1my6,1mzo,1n0t_2,1n18_1,1n18_2,1n18_3,1n18_4,1n18_5,1n19,1n1a,1n1d_1,1n1d_2,1n2t,1n31,1n3o,1n3p,1n3q,1n5q,1n5s,1n5t,1n5v,1n8k,1n8t,1n92,1nce,1ney,1nkt,1nnq,1npa,1npv,1npw,1nr5_1,1nr5_2,1nrv,1nu6,1nvb_1,1nvb_2,1nvf_1,1nvf_2,1nvt,1nwc,1nwh,1nwi_1,1nwi_2,1nwn,1nxf,1nzc_1,1nzc_2,1o0y,1o4t,1o8c_1,1o9b,1oan,1oas,1odw,1odx,1ofn,1ogx,1ohp_1,1ohp_2,1ohr,1ohs_1,1ohs_2,1oi6,1oke,1oki,1okt,1on1,1on2,1op8_1,1op8_2,1op8_3,1ovd,1oxo,1ozt_1,1p0c,1p0f,1p1q_1,1p1q_2,1p1r_1,1p1r_2,1p1u,1p1v_2,1p1w,1p3w,1p9e,1pa0,1pa3,1pcz,1pdw_1,1pdw_2,1pe0,1pkw,1pl1,1pl2,1pm7,1pn9,1pp2,1pqu_1,1prg,1pro,1psq,1psr,1ptz,1pu0_1,1pu0_2,1pu0_3,1pu0_4,1pu0_5,1px6,1q2x,1q4g,1q4j,1q5t,1q6h,1q6i,1q8o,1q8p,1q8q,1q8s,1q8v,1q98,1qac,1qbr,1qbs,1qbt,1qbu,1qhm,1qi9,1qin,1qjg_1,1qjg_2,1qjg_3,1qks,1qm5,1qmj,1qo8,1qor,1qp8,1qq2,1qr2,1qs4_2,1qti,1qv6,1qv7,1qxh,1qy9_1,1qy9_2,1qyc,1r0e,1r1d,1r2r_2,1r38_1,1r38_2,1r4f,1r5c,1r5d,1r5k_2,1r7a,1r7h,1r8w,1r9d,1r9e,1r9m_2,1rei,1rfu_1,1rfu_2,1rfu_3,1rfu_4,1rwq,1s1a,1s2p,1s44,1sbg,1sda_1,1sda_2,1sei,1sfn,1sfy_1,1sfy_2,1sfy_3,1sfy_4,1sg0,1siv,1siz,1sla,1slb_1,1slb_2,1slc_1,1sm9_1,1sm9_2,1so6,1som,1sos_1,1sos_2,1sos_3,1sos_4,1sos_5,1spd,1su2,1su5,1sux,1sw0,1sw3,1sw7,1sxn,1sxs,1sxz,1syi,1syn,1sz3,1t47,1t4b,1t4d_1,1t4d_2,1t6n,1t7k,1t8p,1tar,1tas,1tbp,1tcw,1tcx,1td2,1tdu,1teh_1,1teh_2,1tim,1tip,1tlb_1,1tlb_2,1tlb_3,1tlc,1tlg,1tls,1tmk,1tog,1tok,1tpd_1,1tpd_2,1tpu,1tpv,1tpw,1tq9,1trd_1,1trd_2,1tsd,1tvd,1tw3,1txt_1,1txt_2,1u0m,1u3q_1,1u3t,1u3u,1u3v,1u3w,1u4j,1u6r,1ucf,1uer_1,1uer_2,1ues_1,1ues_2,1ui7,1ui8,1ukg,1una,1uxl_5,1uxm_1,1uxm_2,1uxm_3,1uxm_4,1uxm_5,1uxm_6,1uys_1,1uyt_1,1uyv_1,1uzz_1,1uzz_2,1v02_1,1v02_2,1v08,1v1o,1v59,1v5x,1vc1,1vew_1,1vew_2,1vga_1,1vga_2,1vhb,1vhd,1vhg,1vij,1vik,1vj2,1w2x_1,1w2z_1,1w2z_2,1w4n,1w5v,1w5w,1w5y,1wbk,1wbm,1wgj,1wmn,1wmo,1wmp,1wmy,1wmz_1,1wmz_2,1woa_1,1woa_2,1wob_1,1wob_2,1wze,1wzi,1x1z,1x28,1x29,1x2a,1x77,1xi2,1xkj,1xl2,1xl5,1xpk_2,1xpl_1,1xpm_1,1xpm_2,1xw5,1y7t,1ydv,1ygp,1ykc,1ypi,1ypp,1za5,1zgn,1zii,1zik,1zil,1zlz,1zop,1zpr,1zt9_1,1zt9_2,1zuc,1zvl,20gs,21gs,22gs,2a4f,2ab6_1,2ab6_2,2ae2,2ahb,2aid,2aj9,2ak7,2al4_1,2al4_2,2al4_3,2al5,2aps,2arb,2arx,2asv,2ati,2auy,2av0,2av3,2av6,2aw3,2ayl,2azd,2bb9,2bbb,2bbq,2bkb_1,2bkb_2,2bpv,2bpw,2bpx,2bpy,2bpz,2bqv,2bsl,2btm,2bx7,2byl_1,2byl_2,2bzs,2c0r,2c1j_1,2c3c,2c3d,2c4j_1,2c4j_2,2c80,2ccy,2cd0,2cej,2cem,2cen,2cfd,2cfg,2cga,2cmo,2cst,2cvq,2cwt,2cwu,2cwv,2d1w,2daa,2dfp,2dld,2dor,2ewp_3,2f1o_1,2f1o_2,2f1o_3,2f1o_4,2f3g,2f5e,2fde,2fjp,2g5p,2g5t,2g63_1,2g63_2,2gdu,2gdv,2ghy,2glr,2gnd,2gsa,2gss,2gst,2gtu,2hha,2hr5,2hyf_2,2i03_1,2i03_2,2i4j,2i4p,2iit,2iiv,2kce,2lig,2mjp,2nac,2nqn,2nqq_1,2nqq_2,2nqs,2nro,2nrs,2o4k,2o4p,2oa7,2oac,2oad,2ohx,2oph,2oqi_1,2oqi_2,2oqv,2oxi,2p9h,2phi,2pk5,2pk6,2pmt_1,2pmt_2,2pwz_2,2qr2,2snw,2sod_1,2sod_2,2tmk,2tpr,2tsc,2tsr_1,2tsr_2,2udp,2ypi,2zta,3aid,3bjl,3bto_1,3bto_2,3daa,3dap,3fyg,3gss,3gst,3lad,3nos,3p2p,3pfl,3pgh_1,3pgh_2,3psr,3sdh,3sdp,3tim,3tmk_1,3vhb,3ypi,4bjl,4daa,4gsa,4gss,4gst,4hbi,4mdh,4nos_1,4nos_2,4phv,4sdh,4tim,4upj,5bj3_1,5bj3_2,5bj4,5csc,5fwg,5gss,5gst,5hbi,5mdh,5p2p,5tim,5upj,6cox,6gss,6gst,6gsu,6gsv,6gsw,6gsx,6gsy,6hbi,6tim,6upj,7aat,7gss,7hbi,7tim,7upj,8aat,8gss_1,8gss_2,8prk,8tim,9aat,9gss,9hvp |
| --- |

Note: In the PDB codes, “_1” refers to 1^st^ biological unit entry and “_2” refers to 2^nd^ biological unit entry and so on and so forth.
