# Supplementary material for: Extent of Structural Asymmetry in Homodimeric Proteins: Prevalence and Relevance
Source: PLoS One. 2012 May 22;7(5):e36688. doi: 10.1371/journal.pone.0036688 (PMC3358323; doi:10.1371/journal.pone.0036688)
Supplement: Dataset S2 — List of PDB codes corresponding to non-redundant dataset of homodimers. The list of PDB codes corresponding to the non-redundant dataset of homodimers used in this study is listed. (DOC) [file pone.0036688.s006.doc]

**Dataset S2: List of PDB codes corresponding to non-r**edundant dataset of homodimers

| 1hvh,1gsf_1,1a71,1mqd_1,1m6j,2aps,1f28_1,1aat,1gv3,1n3o,1ivu,1j2e,1hbi,1d4a_1,1ygp,1fx9,1gd9,1tvd,1mft,1iri_1,1pqu_1,1dor,1ogx,1q4g,2nqn,4mdh,1dk4,1aj5_1,1zvl,1k8c_2,117e,1a78,1h8x,1h49,1xpm_1,1nvb_1,1keu,1u0m,1ekm_1,1mo9,2tpr,1ib6_1,1daa,1qr2,1lq9,1aoz,1dty,1adj_1,1n31,1ekf,1rfu_1,1awb,1mg5,1h8y,1c7z,1ucf,1on2,2zta,1r8w,1h18,1gqi,1uyt_1,1gq1,2gdv,1eyz,1gu7,2dld,1tlb_1,1prg,1ipe,1r5k_2,2c1j_1,1m0s,3tmk_1,1bye_1,1ofn,1a0f,1h91,1fro_1,1ko5,1q98,1wmy,1coz,1jb2,1l5b,2ghy,1g51,1lwh,2gsa,1chm,1oan,1dos,1d6s,1dap,1qy9_1,1nvt,1dbq,1jxi,1gq9,1dqn,1gtv_1,1jlw,1nzc_1,1b78,1pcz,1mka,1nnq,1su2,1qs4_2,1vhb,1bbh,1byf,1zt9_1,1psr,1cdc,1kso,1ha4,1aoj,1h7x_2,1nkt,1qhm,1gpe,1uyv_1,1qo8,1qi9,1llf,1dpg,1ad3,5csc,1bk5,1iax,1kl2,1chw,1fc4,1p3w,1u6r,2nac,1t47,2c0r,1b6s_2,1vhd,1iug,1r0e,1tw3,1qor,1o8c_1,1xkj,1gdh,1r4f,1qyc,1qp8,1p9e,1td2,1izy,1f89,2p9h,1jcz,1byk,1h65_1,1o0y,1zuc,1t8p,1sfn,1b49,1fjh,1dug,1r1d,1eix_1,1gfl,1hw1,1jys,1x1z,1h1y,1so6,1edh,1e2q,1i1c,1v5x,1v1o,1cdd,1dzt,1zop,1oki,1bjf,1vhg,1x77,1qq2,1d1g,2snw,1mk4,2lig,2f3g,1k66,1ecz,1h9r,1sei,1f1c,1dd3_1,2ccy,1lyn,1una,1n1a,1e8i,1msb,1o4t,1vj2,1vc1,1b8c,1nrv,1mr8,1c6o,1e7n,1h0x,2ak7,1gk4_1,1aar,1ht9,1r7h,1fe4,1b8z,1eqt,1siz,1fxr |
| --- |

Note: In the PDB codes, “_1” refers to 1^st^ biological unit entry and “_2” refers to 2^nd^ biological unit entry and so on and so forth.
