# Supplementary material for: Extent of Structural Asymmetry in Homodimeric Proteins: Prevalence and Relevance
Source: PLoS One. 2012 May 22;7(5):e36688. doi: 10.1371/journal.pone.0036688 (PMC3358323; doi:10.1371/journal.pone.0036688)
Supplement: Dataset S3 — List of PDB codes corresponding to non-redundant dataset of homodimers complexed without any ligands. The list of PDB codes corresponding to the non-redundant dataset of homodimers not complexed with any ligands used in this study is listed. (DOC) [file pone.0036688.s007.doc]

**Dataset S3: List of PDB codes corresponding to non-redundant dataset of homodimers** complexed without any ligands

| 1hsi,2gtu,1m6j,1ozt_1,1bko_1,1aat,1pp2,1tvd,1jiq_1,1t4d_1,1k41,2nro,1iz9,1lbv,1aj5_1,1h8x,1v02_1,2ahb,2pwz_2,1a4u,1ewz_1,1ucf,1zik,1r8w,1uyt_1,1prg,2c1j_1,1q98,1jb2,2ghy,1l0w,1kp0,1h6j,1jlw,1b78,1pcz,1mkb,1cdc,1kso,1ha4,1aoj,1qhm,1uyv_1,5csc,1xkj,1qyc,1izy,1f89,1o0y,1t8p,1sfn,1fjh,1gfl,1v5x,1v1o,2snw,1mk4,2f3g,1sei,1dd3_1,1lyn,1una,1n1a,1vc1,1nrv,1e7n,1h0x,1aar,1r7h,1b8z |
| --- |

Note: In the PDB codes, “_1” refers to 1^st^ biological unit entry and “_2” refers to 2^nd^ biological unit entry and so on and so forth.
