# Supplementary material for: Extent of Structural Asymmetry in Homodimeric Proteins: Prevalence and Relevance
Source: PLoS One. 2012 May 22;7(5):e36688. doi: 10.1371/journal.pone.0036688 (PMC3358323; doi:10.1371/journal.pone.0036688)
Supplement: Dataset S4 — List of PDB codes corresponding to non-redundant dataset of homodimers complexed with ligands bound at the interface. The list of PDB codes corresponding to the non-redundant dataset of homodimers complexed with ligands interacting with the dimer interface used in this study is listed. (DOC) [file pone.0036688.s008.doc]

**Dataset S4: List of PDB codes corresponding to non-redundant dataset of homodimers** complexed with ligands bound at the interface

| 1hvh,1k3y,1sux,1tsd,2cst,1fx9,1gde,1dqr,1g0i,1zvl,11bg,1qr2,1n31,1tip,1oi6,1qin,1m5j,2gsa,1chm,1su2,1fc4,1jys,1x1z,2lig |
| --- |
