# Supplementary material for: Extent of Structural Asymmetry in Homodimeric Proteins: Prevalence and Relevance
Source: PLoS One. 2012 May 22;7(5):e36688. doi: 10.1371/journal.pone.0036688 (PMC3358323; doi:10.1371/journal.pone.0036688)
Supplement: Dataset S5 — List of PDB codes corresponding to non-redundant dataset of homodimers complexed with ligands bound away from the interface. The list of PDB codes corresponding to the non-redundant dataset of homodimers complexed with ligands bound at regions away from the dimer interface used in this study is listed. (DOC) [file pone.0036688.s009.doc]

**Dataset S5: List of PDB codes corresponding to non-redundant dataset of homodimers** complexed with ligands bound away from the interface

| 1hsh_1, 1ags, 1ju9, 1mqd_1, 1trd_1, 1f28_1, 1n3p, 1d4a_1, 5p2p, 1dor, 1ohp_1, 1q4g, 1alw, 1m9m, 1a78, 1h49, 1nr5_1, 1keu, 1ib6_1, 2gdv, 1eyz, 2i4j, 1ipe, 1u3q_1, 1m0s, 1tmk, 1fro_1, 1ko5, 1wmz_1, 1n1d_1, 1g51, 1oke, 1nvt, 1dqn, 1gtv_1, 1nzc_1, 2mjp, 1qs4_2, 1tlg, 1zt9_1, 1qo8, 1llf, 1chw, 1u6r, 1vhd, 1r0e, 1r4f, 2p9h, 1byk, 1h65_1, 1zuc, 1eix_1, 1e2q, 1d1g, 1o4t |
| --- |

Note: In the PDB codes, “_1” refers to 1^st^ biological unit entry and “_2” refers to 2^nd^ biological unit entry and so on and so forth.
