# Supplementary material for: Extent of Structural Asymmetry in Homodimeric Proteins: Prevalence and Relevance
Source: PLoS One. 2012 May 22;7(5):e36688. doi: 10.1371/journal.pone.0036688 (PMC3358323; doi:10.1371/journal.pone.0036688)
Supplement: Dataset S6 — List of PDB codes corresponding to the pairs of identical homodimers solved in the same crystallographic space group. List of PDB codes corresponding to the pairs of identical homodimeric proteins solved in the same crystallographic space group is listed along with details of the space group and GloA_Sc. (DOC) [file pone.0036688.s010.doc]

Dataset S6: Details of pairs of homodimers solved in the same crystallographic space group.

| **PDB code1** | **PDB code2** | **Space Group** | **GloA_Sc1** | **GloA_Sc2** | **|∆GloA_Sc|** |
| --- | --- | --- | --- | --- | --- |
| 1ajv | 1ajx | P21212 | 0.37 | 0.29 | 0.08 |
| 1ajv | 1c70 | P21212 | 0.37 | 0.39 | 0.02 |
| 1ajx | 1c70 | P21212 | 0.29 | 0.39 | 0.1 |
| 1ajx | 1d4h | P21212 | 0.29 | 0.24 | 0.05 |
| 1c70 | 1d4h | P21212 | 0.39 | 0.24 | 0.15 |
| 1c70 | 1d4i | P21212 | 0.39 | 0.24 | 0.15 |
| 1d4h | 1d4i | P21212 | 0.24 | 0.24 | 0 |
| 1d4h | 1d4j | P21212 | 0.24 | 0.27 | 0.03 |
| 1d4i | 1d4j | P21212 | 0.24 | 0.27 | 0.03 |
| 1d4i | 1ebw | P21212 | 0.24 | 0.22 | 0.02 |
| 1d4j | 1ebw | P21212 | 0.27 | 0.22 | 0.05 |
| 1d4j | 1ebz | P21212 | 0.27 | 0.24 | 0.03 |
| 1dif | 1hih | P212121 | 0.27 | 0.42 | 0.15 |
| 1dif | 1hpx | P212121 | 0.27 | 0.34 | 0.07 |
| 1ebw | 1ebz | P21212 | 0.22 | 0.24 | 0.02 |
| 1ebw | 1ec0 | P21212 | 0.22 | 0.23 | 0.01 |
| 1ebz | 1ec0 | P21212 | 0.24 | 0.23 | 0.01 |
| 1ebz | 1ec1 | P21212 | 0.24 | 0.25 | 0.01 |
| 1ec0 | 1ec1 | P21212 | 0.23 | 0.25 | 0.02 |
| 1ec0 | 1ec2 | P21212 | 0.23 | 0.26 | 0.03 |
| 1ec1 | 1ec2 | P21212 | 0.25 | 0.26 | 0.01 |
| 1ec1 | 1ec3 | P21212 | 0.25 | 0.26 | 0.01 |
| 1ec2 | 1ec3 | P21212 | 0.26 | 0.26 | 0 |
| 1ec2 | 1g2k | P21212 | 0.26 | 0.34 | 0.08 |
| 1ec3 | 1g2k | P21212 | 0.26 | 0.34 | 0.08 |
| 1ec3 | 1g35 | P21212 | 0.26 | 0.33 | 0.07 |
| 1g2k | 1g35 | P21212 | 0.34 | 0.33 | 0.01 |
| 1g2k | 1hsg | P21212 | 0.34 | 0.3 | 0.04 |
| 1g35 | 1hsg | P21212 | 0.33 | 0.3 | 0.03 |
| 1g35 | 1htg | P21212 | 0.33 | 0.23 | 0.1 |
| 1gno | 1hbv | P61 | 0.19 | 0.17 | 0.02 |
| 1gno | 1hos | P61 | 0.19 | 0.16 | 0.03 |
| 1hbv | 1hos | P61 | 0.17 | 0.16 | 0.01 |
| 1hbv | 1hps | P61 | 0.17 | 0.26 | 0.09 |
| 1hih | 1hpx | P212121 | 0.42 | 0.34 | 0.08 |
| 1hih | 1hvi | P212121 | 0.42 | 0.29 | 0.13 |
| 1hos | 1hps | P61 | 0.16 | 0.26 | 0.1 |
| 1hos | 1hpv | P61 | 0.16 | 0.13 | 0.03 |
| 1hps | 1hpv | P61 | 0.26 | 0.13 | 0.13 |
| 1hps | 1htf | P61 | 0.26 | 0.12 | 0.14 |
| 1hpv | 1htf | P61 | 0.13 | 0.12 | 0.01 |
| 1hpv | 1mui | P61 | 0.13 | 0.2 | 0.07 |
| 1hpx | 1hvi | P212121 | 0.34 | 0.29 | 0.05 |
| 1hpx | 1hvj | P212121 | 0.34 | 0.32 | 0.02 |
| 1hsg | 1htg | P21212 | 0.3 | 0.23 | 0.07 |
| 1hsg | 1npv | P21212 | 0.3 | 0.45 | 0.15 |
| 1htf | 1mui | P61 | 0.12 | 0.2 | 0.08 |
| 1htf | 2fde | P61 | 0.12 | 0.19 | 0.07 |
| 1htg | 1npv | P21212 | 0.23 | 0.45 | 0.22 |
| 1htg | 1npw | P21212 | 0.23 | 0.29 | 0.06 |
| 1hvi | 1hvj | P212121 | 0.29 | 0.32 | 0.03 |
| 1hvi | 1hvk | P212121 | 0.29 | 0.28 | 0.01 |
| 1hvj | 1hvk | P212121 | 0.32 | 0.28 | 0.04 |
| 1hvj | 1hvl | P212121 | 0.32 | 0.28 | 0.04 |
| 1hvk | 1hvl | P212121 | 0.28 | 0.28 | 0 |
| 1hvk | 1npa | P212121 | 0.28 | 0.29 | 0.01 |
| 1hvl | 1npa | P212121 | 0.28 | 0.29 | 0.01 |
| 1hvl | 1ohr | P212121 | 0.28 | 0.31 | 0.03 |
| 1mui | 2fde | P61 | 0.2 | 0.19 | 0.01 |
| 1npa | 1ohr | P212121 | 0.29 | 0.31 | 0.02 |
| 1npa | 1xl2 | P212121 | 0.29 | 0.66 | 0.37 |
| 1npv | 1npw | P21212 | 0.45 | 0.29 | 0.16 |
| 1npv | 1t7k | P21212 | 0.45 | 0.37 | 0.08 |
| 1npw | 1t7k | P21212 | 0.29 | 0.37 | 0.08 |
| 1npw | 1w5v | P21212 | 0.29 | 0.23 | 0.06 |
| 1ohr | 1xl2 | P212121 | 0.31 | 0.66 | 0.35 |
| 1t7k | 1w5v | P21212 | 0.37 | 0.23 | 0.14 |
| 1t7k | 1w5w | P21212 | 0.37 | 0.24 | 0.13 |
| 1w5v | 1w5w | P21212 | 0.23 | 0.24 | 0.01 |
| 1w5v | 1w5y | P21212 | 0.23 | 0.24 | 0.01 |
| 1w5w | 1w5y | P21212 | 0.24 | 0.24 | 0 |
| 1w5w | 1wbk | P21212 | 0.24 | 0.24 | 0 |
| 1w5y | 1wbk | P21212 | 0.24 | 0.24 | 0 |
| 1w5y | 1wbm | P21212 | 0.24 | 0.23 | 0.01 |
| 1wbk | 1wbm | P21212 | 0.24 | 0.23 | 0.01 |
| 1wbk | 1xl5 | P21212 | 0.24 | 0.34 | 0.1 |
| 1wbm | 1xl5 | P21212 | 0.23 | 0.34 | 0.11 |
| 1wbm | 2a4f | P21212 | 0.23 | 0.32 | 0.09 |
| 1xl5 | 2a4f | P21212 | 0.34 | 0.32 | 0.02 |
| 1xl5 | 2bb9 | P21212 | 0.34 | 0.26 | 0.08 |
| 2a4f | 2bb9 | P21212 | 0.32 | 0.26 | 0.06 |
| 2a4f | 2bbb | P21212 | 0.32 | 0.37 | 0.05 |
| 2bb9 | 2bbb | P21212 | 0.26 | 0.37 | 0.11 |
| 2bb9 | 2bpv | P21212 | 0.26 | 0.26 | 0 |
| 2bbb | 2bpv | P21212 | 0.37 | 0.26 | 0.11 |
| 2bbb | 2bpw | P21212 | 0.37 | 0.28 | 0.09 |
| 2bpv | 2bpw | P21212 | 0.26 | 0.28 | 0.02 |
| 2bpv | 2bpx | P21212 | 0.26 | 0.36 | 0.1 |
| 2bpw | 2bpx | P21212 | 0.28 | 0.36 | 0.08 |
| 2bpw | 2bpy | P21212 | 0.28 | 0.28 | 0 |
| 2bpx | 2bpy | P21212 | 0.36 | 0.28 | 0.08 |
| 2bpx | 2bpz | P21212 | 0.36 | 0.29 | 0.07 |
| 2bpy | 2bpz | P21212 | 0.28 | 0.29 | 0.01 |
| 2bpy | 2bqv | P21212 | 0.28 | 0.25 | 0.03 |
| 2bpz | 2bqv | P21212 | 0.29 | 0.25 | 0.04 |
| 2bpz | 2cej | P21212 | 0.29 | 0.32 | 0.03 |
| 2bqv | 2cej | P21212 | 0.25 | 0.32 | 0.07 |
| 2bqv | 2cem | P21212 | 0.25 | 0.24 | 0.01 |
| 2cej | 2cem | P21212 | 0.32 | 0.24 | 0.08 |
| 2cej | 2cen | P21212 | 0.32 | 0.25 | 0.07 |
| 2cem | 2cen | P21212 | 0.24 | 0.25 | 0.01 |
| 2cem | 7upj | P21212 | 0.24 | 0.31 | 0.07 |
| 2cen | 7upj | P21212 | 0.25 | 0.31 | 0.06 |
| 1mm7_1 | 1ms7_1 | P21212 | 0.21 | 0.18 | 0.03 |
| 1mm7_1 | 1ms7_2 | P21212 | 0.21 | 0 | 0.21 |
| 1ms7_1 | 1ms7_2 | P21212 | 0.18 | 0 | 0.18 |
| 1ms7_1 | 1mxu_1 | P21212 | 0.18 | 0.31 | 0.13 |
| 1ms7_2 | 1mxu_1 | P21212 | 0 | 0.31 | 0.31 |
| 1ms7_2 | 1mxv_1 | P21212 | 0 | 0.17 | 0.17 |
| 1mxu_1 | 1mxv_1 | P21212 | 0.31 | 0.17 | 0.14 |
| 1mxu_1 | 1mxw_1 | P21212 | 0.31 | 0.17 | 0.14 |
| 1mxv_1 | 1mxw_1 | P21212 | 0.17 | 0.17 | 0 |
| 1mxv_1 | 1mxx_1 | P21212 | 0.17 | 0.18 | 0.01 |
| 1mxw_1 | 1mxx_1 | P21212 | 0.17 | 0.18 | 0.01 |
| 1mxw_1 | 1mxy_1 | P21212 | 0.17 | 0.16 | 0.01 |
| 1mxx_1 | 1mxy_1 | P21212 | 0.18 | 0.16 | 0.02 |
| 1mxx_1 | 1mxz_1 | P21212 | 0.18 | 0.14 | 0.04 |
| 1mxy_1 | 1mxz_1 | P21212 | 0.16 | 0.14 | 0.02 |
| 1mxy_1 | 1mxz_2 | P21212 | 0.16 | 0 | 0.16 |
| 1mxz_1 | 1mxz_2 | P21212 | 0.14 | 0 | 0.14 |
| 1mxz_1 | 1my0_1 | P21212 | 0.14 | 0.15 | 0.01 |
| 1mxz_2 | 1my0_1 | P21212 | 0 | 0.15 | 0.15 |
| 1mxz_2 | 1my0_2 | P21212 | 0 | 0 | 0 |
| 1my0_1 | 1my0_2 | P21212 | 0.15 | 0 | 0.15 |
| 1my0_1 | 1my1_1 | P21212 | 0.15 | 0.14 | 0.01 |
| 1my0_2 | 1my1_1 | P21212 | 0 | 0.14 | 0.14 |
| 1my0_2 | 1my1_2 | P21212 | 0 | 0 | 0 |
| 1my1_1 | 1my1_2 | P21212 | 0.14 | 0 | 0.14 |
| 1my1_1 | 1my2_1 | P21212 | 0.14 | 0.14 | 0 |
| 1my1_2 | 1my2_1 | P21212 | 0 | 0.14 | 0.14 |
| 1my1_2 | 1my2_2 | P21212 | 0 | 0 | 0 |
| 1my2_1 | 1my2_2 | P21212 | 0.14 | 0 | 0.14 |
| 1my2_1 | 1my3_1 | P21212 | 0.14 | 0.28 | 0.14 |
| 1my2_2 | 1my3_1 | P21212 | 0 | 0.28 | 0.28 |
| 1my2_2 | 1my4_1 | P21212 | 0 | 0.3 | 0.3 |
| 1my3_1 | 1my4_1 | P21212 | 0.28 | 0.3 | 0.02 |
| 2al4_1 | 2al4_3 | P1211 | 0.2 | 0.2 | 0 |
| 1adb | 1adc | P1 | 0.16 | 0.12 | 0.04 |
| 1adb | 1n92 | P1 | 0.16 | 0.08 | 0.08 |
| 1adc | 1n92 | P1 | 0.12 | 0.08 | 0.04 |
| 1adc | 2ohx | P1 | 0.12 | 0.18 | 0.06 |
| 1bto_1 | 1bto_2 | P1211 | 0.1 | 0.1 | 0 |
| 1bto_1 | 1hld | P1211 | 0.1 | 0.14 | 0.04 |
| 1bto_2 | 1hld | P1211 | 0.1 | 0.14 | 0.04 |
| 1bto_2 | 1ldy_1 | P1211 | 0.1 | 0.1 | 0 |
| 1hld | 1ldy_1 | P1211 | 0.14 | 0.1 | 0.04 |
| 1hld | 1ldy_2 | P1211 | 0.14 | 0.1 | 0.04 |
| 1ldy_1 | 1ldy_2 | P1211 | 0.1 | 0.1 | 0 |
| 1ldy_1 | 1mg0_1 | P1211 | 0.1 | 0.15 | 0.05 |
| 1ldy_2 | 1mg0_1 | P1211 | 0.1 | 0.15 | 0.05 |
| 1ldy_2 | 1mg0_2 | P1211 | 0.1 | 0.15 | 0.05 |
| 1mg0_1 | 1mg0_2 | P1211 | 0.15 | 0.15 | 0 |
| 1mg0_1 | 1p1r_1 | P1211 | 0.15 | 0.14 | 0.01 |
| 1mg0_2 | 1p1r_1 | P1211 | 0.15 | 0.14 | 0.01 |
| 1mg0_2 | 1p1r_2 | P1211 | 0.15 | 0.12 | 0.03 |
| 1n92 | 2ohx | P1 | 0.08 | 0.18 | 0.1 |
| 1p1r_1 | 1p1r_2 | P1211 | 0.14 | 0.12 | 0.02 |
| 1p1r_1 | 2oxi | P1211 | 0.14 | 0.14 | 0 |
| 1p1r_2 | 2oxi | P1211 | 0.12 | 0.14 | 0.02 |
| 1p1r_2 | 3bto_1 | P1211 | 0.12 | 0.14 | 0.02 |
| 2oxi | 3bto_1 | P1211 | 0.14 | 0.14 | 0 |
| 2oxi | 3bto_2 | P1211 | 0.14 | 0.14 | 0 |
| 3bto_1 | 3bto_2 | P1211 | 0.14 | 0.14 | 0 |
| 1d4a_1 | 1d4a_2 | P1 | 0.18 | 0.2 | 0.02 |
| 1d4a_1 | 1dxo_1 | P1 | 0.18 | 0.12 | 0.06 |
| 1d4a_2 | 1dxo_1 | P1 | 0.2 | 0.12 | 0.08 |
| 1d4a_2 | 1dxo_2 | P1 | 0.2 | 0.14 | 0.06 |
| 1dxo_1 | 1dxo_2 | P1 | 0.12 | 0.14 | 0.02 |
| 1dxo_1 | 1gg5_1 | P1 | 0.12 | 0.15 | 0.03 |
| 1dxo_2 | 1gg5_1 | P1 | 0.14 | 0.15 | 0.01 |
| 1dxo_2 | 1gg5_2 | P1 | 0.14 | 0.18 | 0.04 |
| 1gg5_1 | 1gg5_2 | P1 | 0.15 | 0.18 | 0.03 |
| 1gg5_1 | 1kbo_1 | P1 | 0.15 | 0.31 | 0.16 |
| 1gg5_2 | 1kbo_1 | P1 | 0.18 | 0.31 | 0.13 |
| 1gg5_2 | 1kbo_2 | P1 | 0.18 | 0.28 | 0.1 |
| 1kbo_1 | 1kbo_2 | P1 | 0.31 | 0.28 | 0.03 |
| 1kbo_1 | 2f1o_1 | P1 | 0.31 | 0.19 | 0.12 |
| 1kbo_2 | 2f1o_1 | P1 | 0.28 | 0.19 | 0.09 |
| 1kbo_2 | 2f1o_2 | P1 | 0.28 | 0.22 | 0.06 |
| 2f1o_1 | 2f1o_2 | P1 | 0.19 | 0.22 | 0.03 |
| 2f1o_1 | 2f1o_3 | P1 | 0.19 | 0.22 | 0.03 |
| 2f1o_2 | 2f1o_3 | P1 | 0.22 | 0.22 | 0 |
| 2f1o_2 | 2f1o_4 | P1 | 0.22 | 0.27 | 0.05 |
| 2f1o_3 | 2f1o_4 | P1 | 0.22 | 0.27 | 0.05 |
| 11gs | 16gs | C121 | 0.05 | 0.06 | 0.01 |
| 11gs | 18gs | C121 | 0.05 | 0.09 | 0.04 |
| 11gs | 16gs | C121 | 0.05 | 0.06 | 0.01 |
| 11gs | 18gs | C121 | 0.05 | 0.09 | 0.04 |
| 11gs | 19gs | C121 | 0.05 | 0.05 | 0 |
| 16gs | 18gs | C121 | 0.06 | 0.09 | 0.03 |
| 16gs | 19gs | C121 | 0.06 | 0.05 | 0.01 |
| 16gs | 18gs | C121 | 0.06 | 0.09 | 0.03 |
| 16gs | 19gs | C121 | 0.06 | 0.05 | 0.01 |
| 16gs | 1zgn | C121 | 0.06 | 0.16 | 0.1 |
| 18gs | 19gs | C121 | 0.09 | 0.05 | 0.04 |
| 18gs | 1zgn | C121 | 0.09 | 0.16 | 0.07 |
| 18gs | 19gs | C121 | 0.09 | 0.05 | 0.04 |
| 18gs | 1zgn | C121 | 0.09 | 0.16 | 0.07 |
| 18gs | 20gs | C121 | 0.09 | 0.05 | 0.04 |
| 19gs | 1zgn | C121 | 0.05 | 0.16 | 0.11 |
| 19gs | 20gs | C121 | 0.05 | 0.05 | 0 |
| 19gs | 1zgn | C121 | 0.05 | 0.16 | 0.11 |
| 19gs | 20gs | C121 | 0.05 | 0.05 | 0 |
| 1zgn | 20gs | C121 | 0.16 | 0.05 | 0.11 |
| 1zgn | 2gss | C121 | 0.16 | 0.05 | 0.11 |
| 1zgn | 20gs | C121 | 0.16 | 0.05 | 0.11 |
| 1zgn | 2gss | C121 | 0.16 | 0.05 | 0.11 |
| 20gs | 2gss | C121 | 0.05 | 0.05 | 0 |
| 20gs | 3gss | C121 | 0.05 | 0.07 | 0.02 |
| 20gs | 2gss | C121 | 0.05 | 0.05 | 0 |
| 20gs | 3gss | C121 | 0.05 | 0.07 | 0.02 |
| 2gss | 3gss | C121 | 0.05 | 0.07 | 0.02 |
| 2gss | 5gss | C121 | 0.05 | 0.02 | 0.03 |
| 3gss | 5gss | C121 | 0.07 | 0.02 | 0.05 |
| 3gss | 6gss | C121 | 0.07 | 0.06 | 0.01 |
| 5gss | 6gss | C121 | 0.02 | 0.06 | 0.04 |
| 5gss | 9gss | C121 | 0.02 | 0.08 | 0.06 |
| 6gss | 9gss | C121 | 0.06 | 0.08 | 0.02 |
| 1ag1 | 1iig | P212121 | 0.3 | 0.3 | 0 |
| 1ag1 | 1iih | P212121 | 0.3 | 0.31 | 0.01 |
| 1iig | 1iih | P212121 | 0.3 | 0.31 | 0.01 |
| 1iig | 4tim | P212121 | 0.3 | 0.29 | 0.01 |
| 1iih | 4tim | P212121 | 0.31 | 0.29 | 0.02 |
| 1iih | 5tim | P212121 | 0.31 | 0.27 | 0.04 |
| 1tpd_1 | 1tpd_2 | C121 | 0 | 0 | 0 |
| 1tpd_1 | 1trd_1 | C121 | 0 | 0 | 0 |
| 1tpd_2 | 1trd_1 | C121 | 0 | 0 | 0 |
| 1tpd_2 | 1trd_2 | C121 | 0 | 0 | 0 |
| 1trd_1 | 1trd_2 | C121 | 0 | 0 | 0 |
| 4tim | 5tim | P212121 | 0.29 | 0.27 | 0.02 |
| 4tim | 6tim | P212121 | 0.29 | 0.32 | 0.03 |
| 5tim | 6tim | P212121 | 0.27 | 0.32 | 0.05 |
| 1n18_1 | 1n18_2 | C2221 | 0.19 | 0.24 | 0.05 |
| 1n18_1 | 1n18_3 | C2221 | 0.19 | 0.12 | 0.07 |
| 1n18_2 | 1n18_3 | C2221 | 0.24 | 0.12 | 0.12 |
| 1n18_2 | 1n18_4 | C2221 | 0.24 | 0.19 | 0.05 |
| 1n18_3 | 1n18_4 | C2221 | 0.12 | 0.19 | 0.07 |
| 1n18_3 | 1n18_5 | C2221 | 0.12 | 0.14 | 0.02 |
| 1n18_4 | 1n18_5 | C2221 | 0.19 | 0.14 | 0.05 |
| 1n18_4 | 1sos_1 | C2221 | 0.19 | 0.23 | 0.04 |
| 1n18_5 | 1sos_1 | C2221 | 0.14 | 0.23 | 0.09 |
| 1n18_5 | 1sos_2 | C2221 | 0.14 | 0.2 | 0.06 |
| 1sos_1 | 1sos_2 | C2221 | 0.23 | 0.2 | 0.03 |
| 1sos_1 | 1sos_3 | C2221 | 0.23 | 0.16 | 0.07 |
| 1sos_2 | 1sos_3 | C2221 | 0.2 | 0.16 | 0.04 |
| 1sos_2 | 1sos_4 | C2221 | 0.2 | 0.27 | 0.07 |
| 1sos_3 | 1sos_4 | C2221 | 0.16 | 0.27 | 0.11 |
| 1sos_3 | 1sos_5 | C2221 | 0.16 | 0.23 | 0.07 |
| 1sos_4 | 1sos_5 | C2221 | 0.27 | 0.23 | 0.04 |
| 2g5p | 2g5t | P1211 | 0.1 | 0.3 | 0.2 |
| 2g5p | 2g63_1 | P1211 | 0.1 | 0.13 | 0.03 |
| 2g5t | 2g63_1 | P1211 | 0.3 | 0.13 | 0.17 |
| 2g5t | 2g63_2 | P1211 | 0.3 | 0.05 | 0.25 |
| 2g63_1 | 2g63_2 | P1211 | 0.13 | 0.05 | 0.08 |
| 2g63_1 | 2i03_1 | P1211 | 0.13 | 0.31 | 0.18 |
| 2g63_2 | 2i03_1 | P1211 | 0.05 | 0.31 | 0.26 |
| 2g63_2 | 2i03_2 | P1211 | 0.05 | 0.13 | 0.08 |
| 2i03_1 | 2i03_2 | P1211 | 0.31 | 0.13 | 0.18 |
| 2i03_1 | 2oqi_1 | P1211 | 0.31 | 0.14 | 0.17 |
| 2i03_2 | 2oqi_1 | P1211 | 0.13 | 0.14 | 0.01 |
| 2i03_2 | 2oqi_2 | P1211 | 0.13 | 0.05 | 0.08 |
| 2oqi_1 | 2oqi_2 | P1211 | 0.14 | 0.05 | 0.09 |
| 1an5 | 1axw | P63 | 0.21 | 0.23 | 0.02 |
| 1an5 | 1ddu | P63 | 0.21 | 0.27 | 0.06 |
| 1axw | 1ddu | P63 | 0.23 | 0.27 | 0.04 |
| 1axw | 1syn | P63 | 0.23 | 0.3 | 0.07 |
| 1ddu | 1syn | P63 | 0.27 | 0.3 | 0.03 |
| 1ddu | 1tdu | P63 | 0.27 | 0.26 | 0.01 |
| 1syn | 1tdu | P63 | 0.3 | 0.26 | 0.04 |
| 1syn | 1tlc | P63 | 0.3 | 0.27 | 0.03 |
| 1tdu | 1tlc | P63 | 0.26 | 0.27 | 0.01 |
| 1tdu | 1tsd | P63 | 0.26 | 0.23 | 0.03 |
| 1tlc | 1tsd | P63 | 0.27 | 0.23 | 0.04 |
| 1tlc | 2bbq | P63 | 0.27 | 0.27 | 0 |
| 1tsd | 2bbq | P63 | 0.23 | 0.27 | 0.04 |
| 1tsd | 2kce | P63 | 0.23 | 0.24 | 0.01 |
| 2bbq | 2kce | P63 | 0.27 | 0.24 | 0.03 |
| 1mm7_2 | 1mxu_2 | P21212 | 0 | 0 | 0 |
| 1mm7_2 | 1mxv_2 | P21212 | 0 | 0 | 0 |
| 1mxu_2 | 1mxv_2 | P21212 | 0 | 0 | 0 |
| 1mxu_2 | 1mxw_2 | P21212 | 0 | 0 | 0 |
| 1mxv_2 | 1mxw_2 | P21212 | 0 | 0 | 0 |
| 1mxv_2 | 1mxx_2 | P21212 | 0 | 0 | 0 |
| 1mxw_2 | 1mxx_2 | P21212 | 0 | 0 | 0 |
| 1mxw_2 | 1mxy_2 | P21212 | 0 | 0 | 0 |
| 1mxx_2 | 1mxy_2 | P21212 | 0 | 0 | 0 |
| 1mxx_2 | 1my3_2 | P21212 | 0 | 0 | 0 |
| 1mxy_2 | 1my3_2 | P21212 | 0 | 0 | 0 |
| 1mxy_2 | 1my4_2 | P21212 | 0 | 0 | 0 |
| 1my3_2 | 1my4_2 | P21212 | 0 | 0 | 0 |
| 1cbj | 1cob | P212121 | 0.16 | 0.17 | 0.01 |
| 1sda_1 | 1sda_2 | C121 | 0.25 | 0.19 | 0.06 |
| 1sda_1 | 2sod_1 | C121 | 0.25 | 0.44 | 0.19 |
| 1sda_2 | 2sod_1 | C121 | 0.19 | 0.44 | 0.25 |
| 1sda_2 | 2sod_2 | C121 | 0.19 | 0.45 | 0.26 |
| 1sxn | 1sxs | C2221 | 0.17 | 0.18 | 0.01 |
| 1sxn | 1sxz | C2221 | 0.17 | 0.17 | 0 |
| 1sxs | 1sxz | C2221 | 0.18 | 0.17 | 0.01 |
| 2sod_1 | 2sod_2 | C121 | 0.44 | 0.45 | 0.01 |
| 1eby | 1hxw | P21212 | 0.24 | 0.36 | 0.12 |
| 1eby | 1izh | P21212 | 0.24 | 0.25 | 0.01 |
| 1hxw | 1izh | P21212 | 0.36 | 0.25 | 0.11 |
| 1hxw | 1vik | P21212 | 0.36 | 0.37 | 0.01 |
| 1izh | 1vik | P21212 | 0.25 | 0.37 | 0.12 |
| 1izh | 4phv | P21212 | 0.25 | 0.25 | 0 |
| 1pro | 1sbg | P61 | 0.08 | 0.19 | 0.11 |
| 1pro | 1vij | P61 | 0.08 | 0.31 | 0.23 |
| 1sbg | 1vij | P61 | 0.19 | 0.31 | 0.12 |
| 1sbg | 9hvp | P61 | 0.19 | 0.27 | 0.08 |
| 1vij | 9hvp | P61 | 0.31 | 0.27 | 0.04 |
| 1vik | 4phv | P21212 | 0.37 | 0.25 | 0.12 |
| 1iqx | 1iqy | I121 | 0.17 | 0.17 | 0 |
| 1iqx | 1iu7 | I121 | 0.17 | 0.15 | 0.02 |
| 1iqy | 1iu7 | I121 | 0.17 | 0.15 | 0.02 |
| 1iqy | 1ivv | I121 | 0.17 | 0.19 | 0.02 |
| 1iu7 | 1ivv | I121 | 0.15 | 0.19 | 0.04 |
| 1iu7 | 1ivw | I121 | 0.15 | 0.18 | 0.03 |
| 1ivv | 1ivw | I121 | 0.19 | 0.18 | 0.01 |
| 1ivv | 1ivx | I121 | 0.19 | 0.17 | 0.02 |
| 1ivw | 1ivx | I121 | 0.18 | 0.17 | 0.01 |
| 1ivw | 1wmn | I121 | 0.18 | 0.17 | 0.01 |
| 1ivx | 1wmn | I121 | 0.17 | 0.17 | 0 |
| 1ivx | 1wmo | I121 | 0.17 | 0.16 | 0.01 |
| 1wmn | 1wmo | I121 | 0.17 | 0.16 | 0.01 |
| 1l5v | 1l5w | P212121 | 0.17 | 0.2 | 0.03 |
| 1l5v | 1l6i | P212121 | 0.17 | 0.2 | 0.03 |
| 1l5w | 1l6i | P212121 | 0.2 | 0.2 | 0 |
| 1l5w | 2asv | P212121 | 0.2 | 0.2 | 0 |
| 1l6i | 2asv | P212121 | 0.2 | 0.2 | 0 |
| 1l6i | 2av6 | P212121 | 0.2 | 0.2 | 0 |
| 2asv | 2av6 | P212121 | 0.2 | 0.2 | 0 |
| 2asv | 2aw3 | P212121 | 0.2 | 0.22 | 0.02 |
| 2av6 | 2aw3 | P212121 | 0.2 | 0.22 | 0.02 |
| 2av6 | 2azd | P212121 | 0.2 | 0.39 | 0.19 |
| 2aw3 | 2azd | P212121 | 0.22 | 0.39 | 0.17 |
| 1arg | 1asl | P1211 | 0.11 | 0.19 | 0.08 |
| 1arg | 1asm | P1211 | 0.11 | 0.14 | 0.03 |
| 1asl | 1asm | P1211 | 0.19 | 0.14 | 0.05 |
| 1asl | 1asn | P1211 | 0.19 | 0.14 | 0.05 |
| 1asm | 1asn | P1211 | 0.14 | 0.14 | 0 |
| 1x28 | 1x29 | P63 | 0.2 | 0.18 | 0.02 |
| 1x28 | 1x2a | P63 | 0.2 | 0.18 | 0.02 |
| 1x29 | 1x2a | P63 | 0.18 | 0.18 | 0 |
| 1m6w | 1ma0 | P43212 | 0.2 | 0.17 | 0.03 |
| 1m6w | 1mc5 | P43212 | 0.2 | 0.49 | 0.29 |
| 1ma0 | 1mc5 | P43212 | 0.17 | 0.49 | 0.32 |
| 1ma0 | 1mp0 | P43212 | 0.17 | 0.15 | 0.02 |
| 1mc5 | 1mp0 | P43212 | 0.49 | 0.15 | 0.34 |
| 1teh_1 | 1teh_2 | C2221 | 0 | 0 | 0 |
| 1hsh_1 | 1hsh_2 | P1211 | 0.36 | 0.23 | 0.13 |
| 1hsh_1 | 1hsi | P1211 | 0.36 | 0.33 | 0.03 |
| 1hsh_2 | 1hsi | P1211 | 0.23 | 0.33 | 0.1 |
| 1ida | 1idb | P43212 | 0.17 | 0.19 | 0.02 |
| 1ida | 1jld | P43212 | 0.17 | 0.22 | 0.05 |
| 1idb | 1jld | P43212 | 0.19 | 0.22 | 0.03 |
| 1oxo | 1tar | P1 | 0.23 | 0.16 | 0.07 |
| 1oxo | 7aat | P1 | 0.23 | 0.2 | 0.03 |
| 1tar | 7aat | P1 | 0.16 | 0.2 | 0.04 |
| 1tar | 8aat | P1 | 0.16 | 0.21 | 0.05 |
| 7aat | 8aat | P1 | 0.2 | 0.21 | 0.01 |
| 7aat | 9aat | P1 | 0.2 | 0.19 | 0.01 |
| 8aat | 9aat | P1 | 0.21 | 0.19 | 0.02 |
| 1jtq | 1jtu | P63 | 0.23 | 0.25 | 0.02 |
| 1jtq | 1jut | P63 | 0.23 | 0.24 | 0.01 |
| 1jtu | 1jut | P63 | 0.25 | 0.24 | 0.01 |
| 1jtu | 1kce | P63 | 0.25 | 0.24 | 0.01 |
| 1jut | 1kce | P63 | 0.24 | 0.24 | 0 |
| 1jut | 1kzi | P63 | 0.24 | 0.25 | 0.01 |
| 1kce | 1kzi | P63 | 0.24 | 0.25 | 0.01 |
| 1kce | 1tls | P63 | 0.24 | 0.28 | 0.04 |
| 1kzi | 1tls | P63 | 0.25 | 0.28 | 0.03 |
| 1vga_1 | 1vga_2 | P1211 | 0.15 | 0.18 | 0.03 |
| 1woa_1 | 1woa_2 | P212121 | 0.03 | 0.04 | 0.01 |
| 1woa_1 | 1wob_1 | P212121 | 0.03 | 0.1 | 0.07 |
| 1woa_2 | 1wob_1 | P212121 | 0.04 | 0.1 | 0.06 |
| 1woa_2 | 1wob_2 | P212121 | 0.04 | 0.09 | 0.05 |
| 1wob_1 | 1wob_2 | P212121 | 0.1 | 0.09 | 0.01 |
| 1pu0_1 | 1pu0_2 | C2221 | 0.2 | 0.26 | 0.06 |
| 1pu0_1 | 1pu0_3 | C2221 | 0.2 | 0.11 | 0.09 |
| 1pu0_2 | 1pu0_3 | C2221 | 0.26 | 0.11 | 0.15 |
| 1pu0_2 | 1pu0_4 | C2221 | 0.26 | 0.27 | 0.01 |
| 1pu0_3 | 1pu0_4 | C2221 | 0.11 | 0.27 | 0.16 |
| 1pu0_3 | 1pu0_5 | C2221 | 0.11 | 0.14 | 0.03 |
| 1pu0_4 | 1pu0_5 | C2221 | 0.27 | 0.14 | 0.13 |
| 1uxm_1 | 1uxm_2 | P1211 | 0.11 | 0.11 | 0 |
| 1uxm_1 | 1uxm_3 | P1211 | 0.11 | 0.14 | 0.03 |
| 1uxm_2 | 1uxm_3 | P1211 | 0.11 | 0.14 | 0.03 |
| 1uxm_2 | 1uxm_4 | P1211 | 0.11 | 0.28 | 0.17 |
| 1uxm_3 | 1uxm_4 | P1211 | 0.14 | 0.28 | 0.14 |
| 1uxm_3 | 1uxm_5 | P1211 | 0.14 | 0.18 | 0.04 |
| 1uxm_4 | 1uxm_5 | P1211 | 0.28 | 0.18 | 0.1 |
| 1uxm_4 | 1uxm_6 | P1211 | 0.28 | 0.19 | 0.09 |
| 1uxm_5 | 1uxm_6 | P1211 | 0.18 | 0.19 | 0.01 |
| 1hbi | 1nwn | C121 | 0.2 | 0.02 | 0.18 |
| 1hbi | 3sdh | C121 | 0.2 | 0.19 | 0.01 |
| 1nwi_1 | 1nwi_2 | P1211 | 0.2 | 0.12 | 0.08 |
| 1nwn | 3sdh | C121 | 0.02 | 0.19 | 0.17 |
| 1mo9 | 2c3c | P1211 | 0.23 | 0.18 | 0.05 |
| 1mo9 | 2c3d | P1211 | 0.23 | 0.09 | 0.14 |
| 1mok_1 | 1mok_2 | P1 | 0.17 | 0.17 | 0 |
| 2c3c | 2c3d | P1211 | 0.18 | 0.09 | 0.09 |
| 1deh | 1hdx | P1 | 0.15 | 0.17 | 0.02 |
| 1deh | 1hsz | P1 | 0.15 | 0.11 | 0.04 |
| 1hdx | 1hsz | P1 | 0.17 | 0.11 | 0.06 |
| 1hdx | 1u3u | P1 | 0.17 | 0.08 | 0.09 |
| 1hsz | 1u3u | P1 | 0.11 | 0.08 | 0.03 |
| 1hsz | 1u3v | P1 | 0.11 | 0.08 | 0.03 |
| 1u3u | 1u3v | P1 | 0.08 | 0.08 | 0 |
| 1dor | 1jue | P1211 | 0.06 | 0.03 | 0.03 |
| 1dor | 2bsl | P1211 | 0.06 | 0.05 | 0.01 |
| 1jue | 2bsl | P1211 | 0.03 | 0.05 | 0.02 |
| 1jue | 2bx7 | P1211 | 0.03 | 0.05 | 0.02 |
| 2bsl | 2bx7 | P1211 | 0.05 | 0.05 | 0 |
| 2bsl | 2dor | P1211 | 0.05 | 0.03 | 0.02 |
| 2bx7 | 2dor | P1211 | 0.05 | 0.03 | 0.02 |
| 1lbv | 1lbx | P1211 | 0.46 | 0.31 | 0.15 |
| 1lbw | 1lby | P32 | 0.27 | 0.27 | 0 |
| 1lbw | 1lbz | P32 | 0.27 | 0.34 | 0.07 |
| 1lby | 1lbz | P32 | 0.27 | 0.34 | 0.07 |
| 1lzo_1 | 1lzo_2 | P212121 | 0.27 | 0.23 | 0.04 |
| 1m7o | 1m7p | P1211 | 0.18 | 0.17 | 0.01 |
| 1q8o | 1q8p | P212121 | 0.13 | 0.11 | 0.02 |
| 1q8o | 1ukg | P212121 | 0.13 | 0.11 | 0.02 |
| 1q8p | 1ukg | P212121 | 0.11 | 0.11 | 0 |
| 1q8p | 2auy | P212121 | 0.11 | 0.12 | 0.01 |
| 1ukg | 2auy | P212121 | 0.11 | 0.12 | 0.01 |
| 1ukg | 2gnd | P212121 | 0.11 | 0.13 | 0.02 |
| 2auy | 2gnd | P212121 | 0.12 | 0.13 | 0.01 |
| 1qr2 | 1sg0 | P212121 | 0.17 | 0.15 | 0.02 |
| 1qr2 | 1xi2 | P212121 | 0.17 | 0.16 | 0.01 |
| 1sg0 | 1xi2 | P212121 | 0.15 | 0.16 | 0.01 |
| 1sg0 | 2bzs | P212121 | 0.15 | 0.16 | 0.01 |
| 1xi2 | 2bzs | P212121 | 0.16 | 0.16 | 0 |
| 1xi2 | 2qr2 | P212121 | 0.16 | 0.16 | 0 |
| 2bzs | 2qr2 | P212121 | 0.16 | 0.16 | 0 |
| 1xw5 | 2gtu | P1211 | 0.12 | 0.1 | 0.02 |
| 1ykc | 2ab6_1 | P212121 | 0.12 | 0.07 | 0.05 |
| 1ykc | 2ab6_2 | P212121 | 0.12 | 0.17 | 0.05 |
| 2ab6_1 | 2ab6_2 | P212121 | 0.07 | 0.17 | 0.1 |
| 2gst | 3gst | C121 | 0.16 | 0.15 | 0.01 |
| 2gst | 4gst | C121 | 0.16 | 0.2 | 0.04 |
| 3gst | 4gst | C121 | 0.15 | 0.2 | 0.05 |
| 3gst | 5gst | C121 | 0.15 | 0.22 | 0.07 |
| 4gst | 5gst | C121 | 0.2 | 0.22 | 0.02 |
| 4gst | 6gst | C121 | 0.2 | 0.19 | 0.01 |
| 5gst | 6gst | C121 | 0.22 | 0.19 | 0.03 |
| 1d5n_1 | 1d5n_2 | C2221 | 0.2 | 0.29 | 0.09 |
| 1d5n_1 | 1mmm | C2221 | 0.2 | 0.23 | 0.03 |
| 1d5n_2 | 1mmm | C2221 | 0.29 | 0.23 | 0.06 |
| 1d5n_2 | 1vew_1 | C2221 | 0.29 | 0.09 | 0.2 |
| 1mmm | 1vew_1 | C2221 | 0.23 | 0.09 | 0.14 |
| 1mmm | 1vew_2 | C2221 | 0.23 | 0.12 | 0.11 |
| 1vew_1 | 1vew_2 | C2221 | 0.09 | 0.12 | 0.03 |
| 1ohp_1 | 1ohp_2 | P1211 | 0.4 | 0.3 | 0.1 |
| 1ohp_1 | 1qjg_1 | P1211 | 0.4 | 0.31 | 0.09 |
| 1ohp_2 | 1qjg_1 | P1211 | 0.3 | 0.31 | 0.01 |
| 1ohp_2 | 1qjg_2 | P1211 | 0.3 | 0.23 | 0.07 |
| 1qjg_1 | 1qjg_2 | P1211 | 0.31 | 0.23 | 0.08 |
| 1qjg_1 | 1qjg_3 | P1211 | 0.31 | 0.39 | 0.08 |
| 1qjg_2 | 1qjg_3 | P1211 | 0.23 | 0.39 | 0.16 |
| 1lq9 | 1n5q | P212121 | 0.34 | 0.31 | 0.03 |
| 1lq9 | 1n5s | P212121 | 0.34 | 0.32 | 0.02 |
| 1n5q | 1n5s | P212121 | 0.31 | 0.32 | 0.01 |
| 1n5q | 1n5t | P212121 | 0.31 | 0.34 | 0.03 |
| 1n5s | 1n5t | P212121 | 0.32 | 0.34 | 0.02 |
| 1n5s | 1n5v | P212121 | 0.32 | 0.33 | 0.01 |
| 1n5t | 1n5v | P212121 | 0.34 | 0.33 | 0.01 |
| 1d4y | 1hpo | P21212 | 0.23 | 0.31 | 0.08 |
| 1d4y | 1mrw | P21212 | 0.23 | 0.36 | 0.13 |
| 1hpo | 1mrw | P21212 | 0.31 | 0.36 | 0.05 |
| 1hpo | 1msm | P21212 | 0.31 | 0.37 | 0.06 |
| 1mrw | 1msm | P21212 | 0.36 | 0.37 | 0.01 |
| 1mrw | 2pk6 | P21212 | 0.36 | 0.23 | 0.13 |
| 1msm | 2pk6 | P21212 | 0.37 | 0.23 | 0.14 |
| 1hvr | 1hwr | P61 | 0.15 | 0.21 | 0.06 |
| 1hvr | 1qbr | P61 | 0.15 | 0.19 | 0.04 |
| 1hwr | 1qbr | P61 | 0.21 | 0.19 | 0.02 |
| 1hwr | 1qbt | P61 | 0.21 | 0.17 | 0.04 |
| 1qbr | 1qbt | P61 | 0.19 | 0.17 | 0.02 |
| 1qbr | 1qbu | P61 | 0.19 | 0.3 | 0.11 |
| 1qbt | 1qbu | P61 | 0.17 | 0.3 | 0.13 |
| 1jm0_1 | 1jm0_2 | P212121 | 0.26 | 0.46 | 0.2 |
| 1jm0_1 | 1jm0_3 | P212121 | 0.26 | 0.42 | 0.16 |
| 1jm0_2 | 1jm0_3 | P212121 | 0.46 | 0.42 | 0.04 |
| 1jmb_1 | 1jmb_2 | C2221 | 0 | 0.23 | 0.23 |
| 2hha | 2iit | P212121 | 0.43 | 0.47 | 0.04 |
| 2hha | 2iiv | P212121 | 0.43 | 0.46 | 0.03 |
| 2iit | 2iiv | P212121 | 0.47 | 0.46 | 0.01 |
| 2iit | 2oph | P212121 | 0.47 | 0.41 | 0.06 |
| 2iiv | 2oph | P212121 | 0.46 | 0.41 | 0.05 |
| 2cwt | 2cwu | I121 | 0.18 | 0.14 | 0.04 |
| 2cwt | 2cwv | I121 | 0.18 | 0.15 | 0.03 |
| 2cwu | 2cwv | I121 | 0.14 | 0.15 | 0.01 |
| 2cwu | 2d1w | I121 | 0.14 | 0.15 | 0.01 |
| 2cwv | 2d1w | I121 | 0.15 | 0.15 | 0 |
| 1iri_1 | 1iri_2 | P212121 | 0.16 | 0.19 | 0.03 |
| 1iri_1 | 1jiq_1 | P212121 | 0.16 | 0.13 | 0.03 |
| 1iri_2 | 1jiq_1 | P212121 | 0.19 | 0.13 | 0.06 |
| 1iri_2 | 1jiq_2 | P212121 | 0.19 | 0.16 | 0.03 |
| 1jiq_1 | 1jiq_2 | P212121 | 0.13 | 0.16 | 0.03 |
| 1aoz | 1aso | P21212 | 0.15 | 0.14 | 0.01 |
| 1aoz | 1asp | P21212 | 0.15 | 0.15 | 0 |
| 1aso | 1asp | P21212 | 0.14 | 0.15 | 0.01 |
| 1aso | 1asq | P21212 | 0.14 | 0.13 | 0.01 |
| 1asp | 1asq | P21212 | 0.15 | 0.13 | 0.02 |
| 1eqg | 1eqh | I222 | 0.06 | 0.06 | 0 |
| 1eqg | 1ht5 | I222 | 0.06 | 0.15 | 0.09 |
| 1eqh | 1ht5 | I222 | 0.06 | 0.15 | 0.09 |
| 1eqh | 1ht8 | I222 | 0.06 | 0.14 | 0.08 |
| 1ht5 | 1ht8 | I222 | 0.15 | 0.14 | 0.01 |
| 1adj_1 | 1adj_2 | P21212 | 0.49 | 0.38 | 0.11 |
| 1adj_1 | 1ady_1 | P21212 | 0.49 | 0.5 | 0.01 |
| 1adj_2 | 1ady_1 | P21212 | 0.38 | 0.5 | 0.12 |
| 1adj_2 | 1ady_2 | P21212 | 0.38 | 0.35 | 0.03 |
| 1ady_1 | 1ady_2 | P21212 | 0.5 | 0.35 | 0.15 |
| 1ahe | 1ahf | P1211 | 0.11 | 0.12 | 0.01 |
| 1ahe | 1ahx | P1211 | 0.11 | 0.11 | 0 |
| 1ahf | 1ahx | P1211 | 0.12 | 0.11 | 0.01 |
| 1ahf | 1ahy | P1211 | 0.12 | 0.12 | 0 |
| 1ahx | 1ahy | P1211 | 0.11 | 0.12 | 0.01 |
| 1ekf | 1kta | P212121 | 0.32 | 0.34 | 0.02 |
| 1ib6_1 | 1ib6_2 | C121 | 0.24 | 0.18 | 0.06 |
| 1ib6_1 | 1ie3_1 | C121 | 0.24 | 0.28 | 0.04 |
| 1ib6_2 | 1ie3_1 | C121 | 0.18 | 0.28 | 0.1 |
| 1ib6_2 | 1ie3_2 | C121 | 0.18 | 0.23 | 0.05 |
| 1ie3_1 | 1ie3_2 | C121 | 0.28 | 0.23 | 0.05 |
| 1rfu_1 | 1rfu_2 | P43 | 0.16 | 0.37 | 0.21 |
| 1rfu_1 | 1rfu_3 | P43 | 0.16 | 0.32 | 0.16 |
| 1rfu_2 | 1rfu_3 | P43 | 0.37 | 0.32 | 0.05 |
| 1rfu_2 | 1rfu_4 | P43 | 0.37 | 0.2 | 0.17 |
| 1rfu_3 | 1rfu_4 | P43 | 0.32 | 0.2 | 0.12 |
| 2daa | 3daa | P212121 | 0.22 | 0.23 | 0.01 |
| 1h66_1 | 1h66_2 | P1 | 0.21 | 0.15 | 0.06 |
| 1h66_1 | 1h69_1 | P1 | 0.21 | 0.14 | 0.07 |
| 1h66_2 | 1h69_1 | P1 | 0.15 | 0.14 | 0.01 |
| 1h66_2 | 1h69_2 | P1 | 0.15 | 0.16 | 0.01 |
| 1h69_1 | 1h69_2 | P1 | 0.14 | 0.16 | 0.02 |
| 1awb | 1ima | P3221 | 0.16 | 0.13 | 0.03 |
| 1awb | 1imb | P3221 | 0.16 | 0.14 | 0.02 |
| 1ima | 1imb | P3221 | 0.13 | 0.14 | 0.01 |
| 1ima | 1ime | P3221 | 0.13 | 0.17 | 0.04 |
| 1imb | 1ime | P3221 | 0.14 | 0.17 | 0.03 |
| 1sfy_1 | 1sfy_2 | C121 | 0 | 0 | 0 |
| 1sfy_1 | 1sfy_3 | C121 | 0 | 0.13 | 0.13 |
| 1sfy_2 | 1sfy_3 | C121 | 0 | 0.13 | 0.13 |
| 1sfy_2 | 1sfy_4 | C121 | 0 | 0.1 | 0.1 |
| 1sfy_3 | 1sfy_4 | C121 | 0.13 | 0.1 | 0.03 |
| 1eoh_1 | 1eoh_2 | P212121 | 0.19 | 0.44 | 0.25 |
| 1eoh_1 | 1eoh_3 | P212121 | 0.19 | 0.35 | 0.16 |
| 1eoh_2 | 1eoh_3 | P212121 | 0.44 | 0.35 | 0.09 |
| 1eoh_2 | 1eoh_4 | P212121 | 0.44 | 0.37 | 0.07 |
| 1eoh_3 | 1eoh_4 | P212121 | 0.35 | 0.37 | 0.02 |
| 1glp | 1glq | P212121 | 0.13 | 0.1 | 0.03 |
| 1glp | 2glr | P212121 | 0.13 | 0.13 | 0 |
| 1glq | 2glr | P212121 | 0.1 | 0.13 | 0.03 |
| 1jwn_1 | 1jwn_2 | P1211 | 0.2 | 0.14 | 0.06 |
| 1jwn_1 | 1jzk_1 | P1211 | 0.2 | 0.19 | 0.01 |
| 1jwn_2 | 1jzk_1 | P1211 | 0.14 | 0.19 | 0.05 |
| 1jwn_2 | 1jzk_2 | P1211 | 0.14 | 0.11 | 0.03 |
| 1jzk_1 | 1jzk_2 | P1211 | 0.19 | 0.11 | 0.08 |
| 1slb_1 | 1slb_2 | C121 | 0.41 | 0.4 | 0.01 |
| 11bg | 1bsr | P22121 | 0.47 | 0.54 | 0.07 |
| 1r5c | 1r5d | P212121 | 0.32 | 0.34 | 0.02 |
| 1lt1_1 | 1lt1_2 | P212121 | 0.09 | 0.39 | 0.3 |
| 1lt1_1 | 1lt1_3 | P212121 | 0.09 | 0.29 | 0.2 |
| 1lt1_2 | 1lt1_3 | P212121 | 0.39 | 0.29 | 0.1 |
| 1lt1_2 | 1lt1_4 | P212121 | 0.39 | 0.07 | 0.32 |
| 1lt1_3 | 1lt1_4 | P212121 | 0.29 | 0.07 | 0.22 |
| 1gqi | 1gqj | P1 | 0.07 | 0.07 | 0 |
| 1gqi | 1gqk | P1 | 0.07 | 0.07 | 0 |
| 1gqj | 1gqk | P1 | 0.07 | 0.07 | 0 |
| 1w2z_1 | 1w2z_2 | P1211 | 0.07 | 0.09 | 0.02 |
| 2cfd | 2cfg | C121 | 0.12 | 0.05 | 0.07 |
| 1dqr | 1g98 | C2221 | 0.17 | 0.13 | 0.04 |
| 1dqr | 1hox | C2221 | 0.17 | 0.14 | 0.03 |
| 1g98 | 1hox | C2221 | 0.13 | 0.14 | 0.01 |
| 3pgh_1 | 3pgh_2 | P21212 | 0.03 | 0.03 | 0 |
| 1m9m | 1m9q | P212121 | 0.14 | 0.18 | 0.04 |
| 1m9m | 1m9r | P212121 | 0.14 | 0.23 | 0.09 |
| 1m9q | 1m9r | P212121 | 0.18 | 0.23 | 0.05 |
| 1aia | 1aib | P1211 | 0.13 | 0.07 | 0.06 |
| 1aia | 1aic | P1211 | 0.13 | 0.16 | 0.03 |
| 1aib | 1aic | P1211 | 0.07 | 0.16 | 0.09 |
| 1eyz | 1ez1 | P21212 | 0.24 | 0.44 | 0.2 |
| 1eyz | 1kji | P21212 | 0.24 | 0.33 | 0.09 |
| 1ez1 | 1kji | P21212 | 0.44 | 0.33 | 0.11 |
| 1t4d_1 | 1t4d_2 | P21212 | 0.15 | 0 | 0.15 |
| 1iz9 | 1y7t | P212121 | 0.09 | 0.16 | 0.07 |
| 1iz9 | 2cvq | P212121 | 0.09 | 0.16 | 0.07 |
| 1y7t | 2cvq | P212121 | 0.16 | 0.16 | 0 |
| 1tlb_1 | 1tlb_2 | C121 | 0.41 | 0.48 | 0.07 |
| 1tlb_1 | 1tlb_3 | C121 | 0.41 | 0.48 | 0.07 |
| 1tlb_2 | 1tlb_3 | C121 | 0.48 | 0.48 | 0 |
| 1k8c_2 | 1mi3_1 | C121 | 0.35 | 0.2 | 0.15 |
| 1k8c_2 | 1mi3_2 | C121 | 0.35 | 0.12 | 0.23 |
| 1mi3_1 | 1mi3_2 | C121 | 0.2 | 0.12 | 0.08 |
| 1bko_1 | 1bko_2 | P1211 | 0.27 | 0.23 | 0.04 |
| 1prg | 2i4j | C121 | 1.62 | 1.45 | 0.17 |
| 1prg | 2i4p | C121 | 1.62 | 1.48 | 0.14 |
| 2i4j | 2i4p | C121 | 1.45 | 1.48 | 0.03 |
| 1ipe | 1ipf | P6122 | 0.16 | 0.16 | 0 |
| 1ipe | 2ae2 | P6122 | 0.16 | 0.09 | 0.07 |
| 1ipf | 2ae2 | P6122 | 0.16 | 0.09 | 0.07 |
| 1a4u | 1b14 | P1211 | 0.07 | 0.1 | 0.03 |
| 1a4u | 1b15 | P1211 | 0.07 | 0.07 | 0 |
| 1b14 | 1b15 | P1211 | 0.1 | 0.07 | 0.03 |
| 1dk4 | 1g0h | P212121 | 0.42 | 0.32 | 0.1 |
| 1dk4 | 1g0i | P212121 | 0.42 | 0.41 | 0.01 |
| 1g0h | 1g0i | P212121 | 0.32 | 0.41 | 0.09 |
| 1ypi | 2ypi | P1211 | 0.2 | 0.45 | 0.25 |
| 1ypi | 7tim | P1211 | 0.2 | 0.17 | 0.03 |
| 2ypi | 7tim | P1211 | 0.45 | 0.17 | 0.28 |
| 1n3o | 1n3p | P212121 | 0.12 | 0.14 | 0.02 |
| 1n3o | 1n3q | P212121 | 0.12 | 0.14 | 0.02 |
| 1n3p | 1n3q | P212121 | 0.14 | 0.14 | 0 |
| 1q8q | 1q8s | P212121 | 0.1 | 0.11 | 0.01 |
| 1q8q | 1q8v | P212121 | 0.1 | 0.1 | 0 |
| 1q8s | 1q8v | P212121 | 0.11 | 0.1 | 0.01 |
| 1s1a | 2arb | P212121 | 0.12 | 0.1 | 0.02 |
| 1s1a | 2arx | P212121 | 0.12 | 0.12 | 0 |
| 2arb | 2arx | P212121 | 0.1 | 0.12 | 0.02 |
| 1op8_1 | 1op8_2 | P1 | 0.42 | 0.43 | 0.01 |
| 1op8_1 | 1op8_3 | P1 | 0.42 | 0.49 | 0.07 |
| 1op8_2 | 1op8_3 | P1 | 0.43 | 0.49 | 0.06 |
| 1guh_1 | 1guh_2 | C121 | 0.06 | 0.06 | 0 |
| 1guh_1 | 1k3y | C121 | 0.06 | 0.13 | 0.07 |
| 1guh_2 | 1k3y | C121 | 0.06 | 0.13 | 0.07 |
| 1bjm | 3bjl | P212121 | 4.82 | 5.12 | 0.3 |
| 1bjm | 4bjl | P212121 | 4.82 | 3.55 | 1.27 |
| 3bjl | 4bjl | P212121 | 5.12 | 3.55 | 1.57 |
| 8gss_1 | 8gss_2 | C121 | 0.19 | 0 | 0.19 |
| 1fro_1 | 1fro_2 | P43 | 0.02 | 0.02 | 0 |
| 1f1g_1 | 1f1g_2 | P1 | 0.04 | 0.03 | 0.01 |
| 1f1g_1 | 1f1g_3 | P1 | 0.04 | 0.02 | 0.02 |
| 1f1g_2 | 1f1g_3 | P1 | 0.03 | 0.02 | 0.01 |
| 1wmz_1 | 1wmz_2 | P1211 | 0.54 | 0.75 | 0.21 |
| 1n1d_1 | 1n1d_2 | P1 | 0.23 | 0.4 | 0.17 |
| 1fx9 | 1fxf | P31 | 0.24 | 0.18 | 0.06 |
| 1fx9 | 1l8s | P31 | 0.24 | 0.21 | 0.03 |
| 1fxf | 1l8s | P31 | 0.18 | 0.21 | 0.03 |
| 1b0w_1 | 1b0w_2 | C2221 | 0 | 0 | 0 |
| 1b0w_1 | 1b0w_3 | C2221 | 0 | 0 | 0 |
| 1b0w_2 | 1b0w_3 | C2221 | 0 | 0 | 0 |
| 1l5b | 1m5j | P41212 | 0.28 | 0.28 | 0 |
| 1b6l | 1b6m | P212121 | 0.27 | 0.26 | 0.01 |
| 1b6l | 1b6p | P212121 | 0.27 | 0.25 | 0.02 |
| 1b6m | 1b6p | P212121 | 0.26 | 0.25 | 0.01 |
| 1bv9 | 1mer | P61 | 0.17 | 0.15 | 0.02 |
| 1bv9 | 1mes | P61 | 0.17 | 0.11 | 0.06 |
| 1mer | 1mes | P61 | 0.15 | 0.11 | 0.04 |
| 1bwa | 1bwb | P61 | 0.13 | 0.09 | 0.04 |
| 1bwa | 1meu | P61 | 0.13 | 0.16 | 0.03 |
| 1bwb | 1meu | P61 | 0.09 | 0.16 | 0.07 |
| 1c6x | 1c6z | P212121 | 0.14 | 0.24 | 0.1 |
| 4upj | 5upj | P212121 | 0.34 | 0.43 | 0.09 |
| 4upj | 6upj | P212121 | 0.34 | 0.4 | 0.06 |
| 5upj | 6upj | P212121 | 0.43 | 0.4 | 0.03 |
| 1r9d | 1r9e | C2221 | 0.04 | 0.06 | 0.02 |
| 1nu6 | 1rwq | P212121 | 0.2 | 0.21 | 0.01 |
| 1uyt_1 | 1w2x_1 | C121 | 0 | 0 | 0 |
| 1ekm_1 | 1ekm_2 | C2221 | 0.08 | 0 | 0.08 |
| 1ivu | 1wmp | I121 | 0.19 | 0.2 | 0.01 |
| 1g51 | 1l0w | P212121 | 0.25 | 0.14 | 0.11 |
| 1jlh_1 | 1jlh_2 | P212121 | 0.13 | 0.15 | 0.02 |
| 1q4g | 2ayl | I222 | 0.14 | 0.1 | 0.04 |
| 1e4n | 1e56_1 | P212121 | 0.16 | 0.23 | 0.07 |
| 1v02_1 | 1v02_2 | P31 | 0.17 | 0.12 | 0.05 |
| 1jeh | 1v59 | P212121 | 0.32 | 0.22 | 0.1 |
| 1xpm_1 | 1xpm_2 | P1211 | 0.11 | 0.12 | 0.01 |
| 1lwh | 1lwj | I222 | 0.32 | 0.25 | 0.07 |
| 2gsa | 4gsa | P212121 | 0.23 | 0.21 | 0.02 |
| 4nos_1 | 4nos_2 | P212121 | 0.24 | 0.19 | 0.05 |
| 2byl_1 | 2byl_2 | P3221 | 0.14 | 0 | 0.14 |
| 1g8l | 1g8r | P212121 | 0.69 | 1.31 | 0.62 |
| 2nqq_1 | 2nqq_2 | P1211 | 0.4 | 2.82 | 2.42 |
| 1oan | 1oke | P3121 | 0.21 | 0.17 | 0.04 |
| 1nr5_1 | 1nvb_1 | P21212 | 0 | 0 | 0 |
| 1gd9 | 1gde | P212121 | 0.11 | 0.12 | 0.01 |
| 1nvf_1 | 1nvf_2 | P21212 | 0.19 | 0 | 0.19 |
| 1txt_1 | 1txt_2 | P1211 | 0.12 | 0.12 | 0 |
| 1b5p | 1gck | P212121 | 0.13 | 0.49 | 0.36 |
| 1bkg_1 | 1bkg_2 | P212121 | 0.11 | 0.13 | 0.02 |
| 1elq | 1elu | P212121 | 0.13 | 0.15 | 0.02 |
| 1axg_1 | 1axg_2 | P1 | 0.2 | 0.21 | 0.01 |
| 1hso | 1u3t | P1211 | 0.08 | 0.08 | 0 |
| 1ht0 | 1u3w | P1211 | 0.24 | 0.28 | 0.04 |
| 1qv6 | 1qv7 | P1 | 0.09 | 0.1 | 0.01 |
| 1p0c | 1p0f | C121 | 0.14 | 0.13 | 0.01 |
| 5bj3_1 | 5bj3_2 | P1211 | 0.04 | 0.02 | 0.02 |
| 1keu | 1kew | P61 | 0.12 | 0.15 | 0.03 |
| 1g1a_1 | 1g1a_2 | P1211 | 0.2 | 0.14 | 0.06 |
| 4mdh | 5mdh | P21212 | 1.64 | 1.2 | 0.44 |
| 1wze | 1wzi | P212121 | 0.19 | 0.22 | 0.03 |
| 1dap | 3dap | P1211 | 1.3 | 1.31 | 0.01 |
| 1r38_1 | 1r38_2 | C121 | 0.2 | 0.17 | 0.03 |
| 1sm9_1 | 1sm9_2 | C121 | 0.17 | 0.14 | 0.03 |
| 1f28_1 | 1f28_2 | P1211 | 0.17 | 0.16 | 0.01 |
| 1hvy_1 | 1hvy_2 | P1 | 0.22 | 0.21 | 0.01 |
| 2tsr_1 | 2tsr_2 | C121 | 0.32 | 0.24 | 0.08 |
| 1kzj_1 | 1kzj_2 | P1211 | 0.22 | 0.28 | 0.06 |
| 1ftl | 2cmo | P212121 | 0.33 | 1.15 | 0.82 |
| 1i45 | 1ney | P1211 | 0.09 | 0.11 | 0.02 |
| 1gq9 | 1h6j | P1211 | 0.41 | 0.09 | 0.32 |
| 1dqn | 1dqp | P212121 | 0.26 | 0.24 | 0.02 |
| 1gsf_1 | 1gsf_2 | C121 | 0.07 | 0.07 | 0 |
| 1gtv_1 | 1gtv_2 | P6522 | 0 | 0 | 0 |
| 1pkw | 1pl1 | C121 | 0.28 | 0.22 | 0.06 |
| 1c72_1 | 1c72_2 | C2221 | 0.1 | 0.05 | 0.05 |
| 2c4j_1 | 2c4j_2 | P1211 | 0.25 | 0.24 | 0.01 |
| 6gsx | 6gsy | C121 | 0.15 | 0.3 | 0.15 |
| 1bye_1 | 1bye_2 | C121 | 0.56 | 0.41 | 0.15 |
| 1lgv | 1lhz | P212121 | 3.29 | 3.17 | 0.12 |
| 1gsd_1 | 1gsd_2 | C121 | 0.07 | 0.1 | 0.03 |
| 1q6h | 1q6i | P212121 | 3.65 | 3.68 | 0.03 |
| 1en5_1 | 1en5_2 | C2221 | 0.12 | 0.17 | 0.05 |
| 1i08_1 | 1i08_2 | C2221 | 0.12 | 0.2 | 0.08 |
| 1i0h | 1zlz | P1211 | 0.29 | 0.27 | 0.02 |
| 1tmk | 2tmk | P1211 | 0.15 | 0.19 | 0.04 |
| 1ofn | 1oi6 | P21212 | 0.26 | 0.23 | 0.03 |
| 2pmt_1 | 2pmt_2 | P43 | 0.11 | 0.14 | 0.03 |
| 1a4o_1 | 1a4o_2 | P65 | 0.23 | 0.2 | 0.03 |
| 1isa | 1isb | P212121 | 0.12 | 0.12 | 0 |
| 2bkb_1 | 2bkb_2 | P1211 | 0.43 | 0.51 | 0.08 |
| 1uer_1 | 1uer_2 | P212121 | 0.18 | 0.41 | 0.23 |
| 1ues_1 | 1ues_2 | P212121 | 0.47 | 0.49 | 0.02 |
| 1b78 | 2mjp | P212121 | 0.29 | 0.32 | 0.03 |
| 1pdw_1 | 1pdw_2 | P1211 | 0.2 | 0.18 | 0.02 |
| 1h91 | 1s44 | P212121 | 0.25 | 0.24 | 0.01 |
| 1aj5_1 | 1aj5_2 | C2221 | 0 | 0 | 0 |
| 1alv | 1alw | P1211 | 0.39 | 0.35 | 0.04 |
| 1mka | 1mkb | P212121 | 0.12 | 0.14 | 0.02 |
| 1nnq | 2hr5 | P42212 | 0.15 | 0.14 | 0.01 |
| 1vhb | 3vhb | P1211 | 0.15 | 0.18 | 0.03 |
| 1ohs_1 | 1ohs_2 | P1211 | 0.31 | 0.34 | 0.03 |
| 1byf | 1tlg | P212121 | 0.12 | 0.13 | 0.01 |
| 1c1j_1 | 1c1j_2 | C121 | 0.17 | 0.73 | 0.56 |
| 1g0z | 1u4j | H3 | 0.07 | 0.07 | 0 |
| 1zt9_1 | 1zt9_2 | P43 | 0.24 | 0.15 | 0.09 |
| 1bv7 | 1met | P61 | 0.17 | 0.14 | 0.03 |
| 1mrx | 1msn | P21212 | 0.37 | 0.42 | 0.05 |
| 2aid | 3aid | P212121 | 0.37 | 0.35 | 0.02 |
| 1dmp | 1qbs | P61 | 0.22 | 0.14 | 0.08 |
| 1ec5_1 | 1ec5_2 | C2221 | 0 | 0.29 | 0.29 |

In the PDB codes, “_1” and “_2” refers to the biological unit under consideration, for cases where multiple homodimeric biological units are available for a PDB.
