# Supplementary material for: Extent of Structural Asymmetry in Homodimeric Proteins: Prevalence and Relevance
Source: PLoS One. 2012 May 22;7(5):e36688. doi: 10.1371/journal.pone.0036688 (PMC3358323; doi:10.1371/journal.pone.0036688)
Supplement: Dataset S7 — List of PDB codes corresponding to the pairs of identical homodimers solved in different crystallographic space groups. List of PDB codes corresponding to the pairs of identical homodimeric proteins solved in different crystallographic space group is listed along with details of the space groups and GloA_Sc. (DOC) [file pone.0036688.s011.doc]

Dataset S7: Details of pairs of homodimers solved in different crystallographic space groups.

| **PDB code1** | **PDB code2** | **Space Group1** | **Space Group2** | **GloA_Sc1** | **GloA_Sc2** | | **|∆GloA_Sc|** | |
| --- | --- | --- | --- | --- | --- | --- | --- | --- |
| 1ajv | 1dif | P21212 | P212121 | 0.37 | 0.27 | 0.1 | |  |
| 1ajv | 1hih | P21212 | P212121 | 0.37 | 0.42 | 0.05 | |  |
| 1ajv | 1hpx | P21212 | P212121 | 0.37 | 0.34 | 0.03 | |  |
| 1ajv | 1gno | P21212 | P61 | 0.37 | 0.19 | 0.18 | |  |
| 1ajv | 1hbv | P21212 | P61 | 0.37 | 0.17 | 0.2 | |  |
| 1ajv | 1hos | P21212 | P61 | 0.37 | 0.16 | 0.21 | |  |
| 1ajx | 1dif | P21212 | P212121 | 0.29 | 0.27 | 0.02 | |  |
| 1ajx | 1hih | P21212 | P212121 | 0.29 | 0.42 | 0.13 | |  |
| 1ajx | 1hpx | P21212 | P212121 | 0.29 | 0.34 | 0.05 | |  |
| 1ajx | 1gno | P21212 | P61 | 0.29 | 0.19 | 0.1 | |  |
| 1ajx | 1hbv | P21212 | P61 | 0.29 | 0.17 | 0.12 | |  |
| 1ajx | 1hos | P21212 | P61 | 0.29 | 0.16 | 0.13 | |  |
| 1c70 | 1dif | P21212 | P212121 | 0.39 | 0.27 | 0.12 | |  |
| 1c70 | 1hih | P21212 | P212121 | 0.39 | 0.42 | 0.03 | |  |
| 1c70 | 1hpx | P21212 | P212121 | 0.39 | 0.34 | 0.05 | |  |
| 1c70 | 1gno | P21212 | P61 | 0.39 | 0.19 | 0.2 | |  |
| 1c70 | 1hbv | P21212 | P61 | 0.39 | 0.17 | 0.22 | |  |
| 1c70 | 1hos | P21212 | P61 | 0.39 | 0.16 | 0.23 | |  |
| 1d4h | 1dif | P21212 | P212121 | 0.24 | 0.27 | 0.03 | |  |
| 1d4h | 1hih | P21212 | P212121 | 0.24 | 0.42 | 0.18 | |  |
| 1d4h | 1hpx | P21212 | P212121 | 0.24 | 0.34 | 0.1 | |  |
| 1d4h | 1gno | P21212 | P61 | 0.24 | 0.19 | 0.05 | |  |
| 1d4h | 1hbv | P21212 | P61 | 0.24 | 0.17 | 0.07 | |  |
| 1d4h | 1hos | P21212 | P61 | 0.24 | 0.16 | 0.08 | |  |
| 1d4i | 1dif | P21212 | P212121 | 0.24 | 0.27 | 0.03 | |  |
| 1d4i | 1hih | P21212 | P212121 | 0.24 | 0.42 | 0.18 | |  |
| 1d4i | 1hpx | P21212 | P212121 | 0.24 | 0.34 | 0.1 | |  |
| 1d4i | 1gno | P21212 | P61 | 0.24 | 0.19 | 0.05 | |  |
| 1d4i | 1hbv | P21212 | P61 | 0.24 | 0.17 | 0.07 | |  |
| 1d4i | 1hos | P21212 | P61 | 0.24 | 0.16 | 0.08 | |  |
| 1d4j | 1dif | P21212 | P212121 | 0.27 | 0.27 | 0 | |  |
| 1d4j | 1hih | P21212 | P212121 | 0.27 | 0.42 | 0.15 | |  |
| 1d4j | 1hpx | P21212 | P212121 | 0.27 | 0.34 | 0.07 | |  |
| 1d4j | 1gno | P21212 | P61 | 0.27 | 0.19 | 0.08 | |  |
| 1d4j | 1hbv | P21212 | P61 | 0.27 | 0.17 | 0.1 | |  |
| 1d4j | 1hos | P21212 | P61 | 0.27 | 0.16 | 0.11 | |  |
| 1dif | 1ebw | P212121 | P21212 | 0.27 | 0.22 | 0.05 | |  |
| 1dif | 1ebz | P212121 | P21212 | 0.27 | 0.24 | 0.03 | |  |
| 1dif | 1ec0 | P212121 | P21212 | 0.27 | 0.23 | 0.04 | |  |
| 1dif | 1gno | P212121 | P61 | 0.27 | 0.19 | 0.08 | |  |
| 1dif | 1hbv | P212121 | P61 | 0.27 | 0.17 | 0.1 | |  |
| 1dif | 1hos | P212121 | P61 | 0.27 | 0.16 | 0.11 | |  |
| 1ebw | 1hih | P21212 | P212121 | 0.22 | 0.42 | 0.2 | |  |
| 1ebw | 1hpx | P21212 | P212121 | 0.22 | 0.34 | 0.12 | |  |
| 1ebw | 1hvi | P21212 | P212121 | 0.22 | 0.29 | 0.07 | |  |
| 1ebw | 1gno | P21212 | P61 | 0.22 | 0.19 | 0.03 | |  |
| 1ebw | 1hbv | P21212 | P61 | 0.22 | 0.17 | 0.05 | |  |
| 1ebw | 1hos | P21212 | P61 | 0.22 | 0.16 | 0.06 | |  |
| 1ebz | 1hih | P21212 | P212121 | 0.24 | 0.42 | 0.18 | |  |
| 1ebz | 1hpx | P21212 | P212121 | 0.24 | 0.34 | 0.1 | |  |
| 1ebz | 1hvi | P21212 | P212121 | 0.24 | 0.29 | 0.05 | |  |
| 1ebz | 1gno | P21212 | P61 | 0.24 | 0.19 | 0.05 | |  |
| 1ebz | 1hbv | P21212 | P61 | 0.24 | 0.17 | 0.07 | |  |
| 1ebz | 1hos | P21212 | P61 | 0.24 | 0.16 | 0.08 | |  |
| 1ec0 | 1hih | P21212 | P212121 | 0.23 | 0.42 | 0.19 | |  |
| 1ec0 | 1hpx | P21212 | P212121 | 0.23 | 0.34 | 0.11 | |  |
| 1ec0 | 1hvi | P21212 | P212121 | 0.23 | 0.29 | 0.06 | |  |
| 1ec0 | 1gno | P21212 | P61 | 0.23 | 0.19 | 0.04 | |  |
| 1ec0 | 1hbv | P21212 | P61 | 0.23 | 0.17 | 0.06 | |  |
| 1ec0 | 1hos | P21212 | P61 | 0.23 | 0.16 | 0.07 | |  |
| 1ec1 | 1hih | P21212 | P212121 | 0.25 | 0.42 | 0.17 | |  |
| 1ec1 | 1hpx | P21212 | P212121 | 0.25 | 0.34 | 0.09 | |  |
| 1ec1 | 1hvi | P21212 | P212121 | 0.25 | 0.29 | 0.04 | |  |
| 1ec1 | 1gno | P21212 | P61 | 0.25 | 0.19 | 0.06 | |  |
| 1ec1 | 1hbv | P21212 | P61 | 0.25 | 0.17 | 0.08 | |  |
| 1ec1 | 1hos | P21212 | P61 | 0.25 | 0.16 | 0.09 | |  |
| 1ec2 | 1hih | P21212 | P212121 | 0.26 | 0.42 | 0.16 | |  |
| 1ec2 | 1hpx | P21212 | P212121 | 0.26 | 0.34 | 0.08 | |  |
| 1ec2 | 1hvi | P21212 | P212121 | 0.26 | 0.29 | 0.03 | |  |
| 1ec2 | 1gno | P21212 | P61 | 0.26 | 0.19 | 0.07 | |  |
| 1ec2 | 1hbv | P21212 | P61 | 0.26 | 0.17 | 0.09 | |  |
| 1ec2 | 1hos | P21212 | P61 | 0.26 | 0.16 | 0.1 | |  |
| 1ec3 | 1hih | P21212 | P212121 | 0.26 | 0.42 | 0.16 | |  |
| 1ec3 | 1hpx | P21212 | P212121 | 0.26 | 0.34 | 0.08 | |  |
| 1ec3 | 1hvi | P21212 | P212121 | 0.26 | 0.29 | 0.03 | |  |
| 1ec3 | 1gno | P21212 | P61 | 0.26 | 0.19 | 0.07 | |  |
| 1ec3 | 1hbv | P21212 | P61 | 0.26 | 0.17 | 0.09 | |  |
| 1ec3 | 1hos | P21212 | P61 | 0.26 | 0.16 | 0.1 | |  |
| 1g2k | 1hih | P21212 | P212121 | 0.34 | 0.42 | 0.08 | |  |
| 1g2k | 1hpx | P21212 | P212121 | 0.34 | 0.34 | 0 | |  |
| 1g2k | 1hvi | P21212 | P212121 | 0.34 | 0.29 | 0.05 | |  |
| 1g2k | 1gno | P21212 | P61 | 0.34 | 0.19 | 0.15 | |  |
| 1g2k | 1hbv | P21212 | P61 | 0.34 | 0.17 | 0.17 | |  |
| 1g2k | 1hos | P21212 | P61 | 0.34 | 0.16 | 0.18 | |  |
| 1g35 | 1hih | P21212 | P212121 | 0.33 | 0.42 | 0.09 | |  |
| 1g35 | 1hpx | P21212 | P212121 | 0.33 | 0.34 | 0.01 | |  |
| 1g35 | 1hvi | P21212 | P212121 | 0.33 | 0.29 | 0.04 | |  |
| 1g35 | 1gno | P21212 | P61 | 0.33 | 0.19 | 0.14 | |  |
| 1g35 | 1hbv | P21212 | P61 | 0.33 | 0.17 | 0.16 | |  |
| 1g35 | 1hos | P21212 | P61 | 0.33 | 0.16 | 0.17 | |  |
| 1gno | 1hsg | P61 | P21212 | 0.19 | 0.3 | 0.11 | |  |
| 1gno | 1htg | P61 | P21212 | 0.19 | 0.23 | 0.04 | |  |
| 1gno | 1npv | P61 | P21212 | 0.19 | 0.45 | 0.26 | |  |
| 1gno | 1hih | P61 | P212121 | 0.19 | 0.42 | 0.23 | |  |
| 1gno | 1hpx | P61 | P212121 | 0.19 | 0.34 | 0.15 | |  |
| 1gno | 1hvi | P61 | P212121 | 0.19 | 0.29 | 0.1 | |  |
| 1hbv | 1hsg | P61 | P21212 | 0.17 | 0.3 | 0.13 | |  |
| 1hbv | 1htg | P61 | P21212 | 0.17 | 0.23 | 0.06 | |  |
| 1hbv | 1npv | P61 | P21212 | 0.17 | 0.45 | 0.28 | |  |
| 1hbv | 1hih | P61 | P212121 | 0.17 | 0.42 | 0.25 | |  |
| 1hbv | 1hpx | P61 | P212121 | 0.17 | 0.34 | 0.17 | |  |
| 1hbv | 1hvi | P61 | P212121 | 0.17 | 0.29 | 0.12 | |  |
| 1hih | 1hsg | P212121 | P21212 | 0.42 | 0.3 | 0.12 | |  |
| 1hih | 1htg | P212121 | P21212 | 0.42 | 0.23 | 0.19 | |  |
| 1hih | 1npv | P212121 | P21212 | 0.42 | 0.45 | 0.03 | |  |
| 1hih | 1hos | P212121 | P61 | 0.42 | 0.16 | 0.26 | |  |
| 1hih | 1hps | P212121 | P61 | 0.42 | 0.26 | 0.16 | |  |
| 1hih | 1hpv | P212121 | P61 | 0.42 | 0.13 | 0.29 | |  |
| 1hos | 1hsg | P61 | P21212 | 0.16 | 0.3 | 0.14 | |  |
| 1hos | 1htg | P61 | P21212 | 0.16 | 0.23 | 0.07 | |  |
| 1hos | 1npv | P61 | P21212 | 0.16 | 0.45 | 0.29 | |  |
| 1hos | 1hpx | P61 | P212121 | 0.16 | 0.34 | 0.18 | |  |
| 1hos | 1hvi | P61 | P212121 | 0.16 | 0.29 | 0.13 | |  |
| 1hos | 1hvj | P61 | P212121 | 0.16 | 0.32 | 0.16 | |  |
| 1hps | 1hsg | P61 | P21212 | 0.26 | 0.3 | 0.04 | |  |
| 1hps | 1htg | P61 | P21212 | 0.26 | 0.23 | 0.03 | |  |
| 1hps | 1npv | P61 | P21212 | 0.26 | 0.45 | 0.19 | |  |
| 1hps | 1hpx | P61 | P212121 | 0.26 | 0.34 | 0.08 | |  |
| 1hps | 1hvi | P61 | P212121 | 0.26 | 0.29 | 0.03 | |  |
| 1hps | 1hvj | P61 | P212121 | 0.26 | 0.32 | 0.06 | |  |
| 1hpv | 1hsg | P61 | P21212 | 0.13 | 0.3 | 0.17 | |  |
| 1hpv | 1htg | P61 | P21212 | 0.13 | 0.23 | 0.1 | |  |
| 1hpv | 1npv | P61 | P21212 | 0.13 | 0.45 | 0.32 | |  |
| 1hpv | 1hpx | P61 | P212121 | 0.13 | 0.34 | 0.21 | |  |
| 1hpv | 1hvi | P61 | P212121 | 0.13 | 0.29 | 0.16 | |  |
| 1hpv | 1hvj | P61 | P212121 | 0.13 | 0.32 | 0.19 | |  |
| 1hpx | 1hsg | P212121 | P21212 | 0.34 | 0.3 | 0.04 | |  |
| 1hpx | 1htg | P212121 | P21212 | 0.34 | 0.23 | 0.11 | |  |
| 1hpx | 1npv | P212121 | P21212 | 0.34 | 0.45 | 0.11 | |  |
| 1hpx | 1htf | P212121 | P61 | 0.34 | 0.12 | 0.22 | |  |
| 1hpx | 1mui | P212121 | P61 | 0.34 | 0.2 | 0.14 | |  |
| 1hpx | 2fde | P212121 | P61 | 0.34 | 0.19 | 0.15 | |  |
| 1hsg | 1hvi | P21212 | P212121 | 0.3 | 0.29 | 0.01 | |  |
| 1hsg | 1hvj | P21212 | P212121 | 0.3 | 0.32 | 0.02 | |  |
| 1hsg | 1hvk | P21212 | P212121 | 0.3 | 0.28 | 0.02 | |  |
| 1hsg | 1htf | P21212 | P61 | 0.3 | 0.12 | 0.18 | |  |
| 1hsg | 1mui | P21212 | P61 | 0.3 | 0.2 | 0.1 | |  |
| 1hsg | 2fde | P21212 | P61 | 0.3 | 0.19 | 0.11 | |  |
| 1htf | 1htg | P61 | P21212 | 0.12 | 0.23 | 0.11 | |  |
| 1htf | 1npv | P61 | P21212 | 0.12 | 0.45 | 0.33 | |  |
| 1htf | 1npw | P61 | P21212 | 0.12 | 0.29 | 0.17 | |  |
| 1htf | 1hvi | P61 | P212121 | 0.12 | 0.29 | 0.17 | |  |
| 1htf | 1hvj | P61 | P212121 | 0.12 | 0.32 | 0.2 | |  |
| 1htf | 1hvk | P61 | P212121 | 0.12 | 0.28 | 0.16 | |  |
| 1htg | 1hvi | P21212 | P212121 | 0.23 | 0.29 | 0.06 | |  |
| 1htg | 1hvj | P21212 | P212121 | 0.23 | 0.32 | 0.09 | |  |
| 1htg | 1hvk | P21212 | P212121 | 0.23 | 0.28 | 0.05 | |  |
| 1htg | 1mui | P21212 | P61 | 0.23 | 0.2 | 0.03 | |  |
| 1htg | 2fde | P21212 | P61 | 0.23 | 0.19 | 0.04 | |  |
| 1hvi | 1npv | P212121 | P21212 | 0.29 | 0.45 | 0.16 | |  |
| 1hvi | 1npw | P212121 | P21212 | 0.29 | 0.29 | 0 | |  |
| 1hvi | 1t7k | P212121 | P21212 | 0.29 | 0.37 | 0.08 | |  |
| 1hvi | 1mui | P212121 | P61 | 0.29 | 0.2 | 0.09 | |  |
| 1hvi | 2fde | P212121 | P61 | 0.29 | 0.19 | 0.1 | |  |
| 1hvj | 1npv | P212121 | P21212 | 0.32 | 0.45 | 0.13 | |  |
| 1hvj | 1npw | P212121 | P21212 | 0.32 | 0.29 | 0.03 | |  |
| 1hvj | 1t7k | P212121 | P21212 | 0.32 | 0.37 | 0.05 | |  |
| 1hvj | 1mui | P212121 | P61 | 0.32 | 0.2 | 0.12 | |  |
| 1hvj | 2fde | P212121 | P61 | 0.32 | 0.19 | 0.13 | |  |
| 1hvk | 1npv | P212121 | P21212 | 0.28 | 0.45 | 0.17 | |  |
| 1hvk | 1npw | P212121 | P21212 | 0.28 | 0.29 | 0.01 | |  |
| 1hvk | 1t7k | P212121 | P21212 | 0.28 | 0.37 | 0.09 | |  |
| 1hvk | 1mui | P212121 | P61 | 0.28 | 0.2 | 0.08 | |  |
| 1hvk | 2fde | P212121 | P61 | 0.28 | 0.19 | 0.09 | |  |
| 1hvl | 1npv | P212121 | P21212 | 0.28 | 0.45 | 0.17 | |  |
| 1hvl | 1npw | P212121 | P21212 | 0.28 | 0.29 | 0.01 | |  |
| 1hvl | 1t7k | P212121 | P21212 | 0.28 | 0.37 | 0.09 | |  |
| 1hvl | 1mui | P212121 | P61 | 0.28 | 0.2 | 0.08 | |  |
| 1hvl | 2fde | P212121 | P61 | 0.28 | 0.19 | 0.09 | |  |
| 1mui | 1npv | P61 | P21212 | 0.2 | 0.45 | 0.25 | |  |
| 1mui | 1npw | P61 | P21212 | 0.2 | 0.29 | 0.09 | |  |
| 1mui | 1t7k | P61 | P21212 | 0.2 | 0.37 | 0.17 | |  |
| 1mui | 1npa | P61 | P212121 | 0.2 | 0.29 | 0.09 | |  |
| 1mui | 1ohr | P61 | P212121 | 0.2 | 0.31 | 0.11 | |  |
| 1mui | 1xl2 | P61 | P212121 | 0.2 | 0.66 | 0.46 | |  |
| 1npa | 1npv | P212121 | P21212 | 0.29 | 0.45 | 0.16 | |  |
| 1npa | 1npw | P212121 | P21212 | 0.29 | 0.29 | 0 | |  |
| 1npa | 1t7k | P212121 | P21212 | 0.29 | 0.37 | 0.08 | |  |
| 1npa | 2fde | P212121 | P61 | 0.29 | 0.19 | 0.1 | |  |
| 1npv | 1ohr | P21212 | P212121 | 0.45 | 0.31 | 0.14 | |  |
| 1npv | 1xl2 | P21212 | P212121 | 0.45 | 0.66 | 0.21 | |  |
| 1npv | 2fde | P21212 | P61 | 0.45 | 0.19 | 0.26 | |  |
| 1npw | 1ohr | P21212 | P212121 | 0.29 | 0.31 | 0.02 | |  |
| 1npw | 1xl2 | P21212 | P212121 | 0.29 | 0.66 | 0.37 | |  |
| 1npw | 2fde | P21212 | P61 | 0.29 | 0.19 | 0.1 | |  |
| 1ohr | 1t7k | P212121 | P21212 | 0.31 | 0.37 | 0.06 | |  |
| 1ohr | 1w5v | P212121 | P21212 | 0.31 | 0.23 | 0.08 | |  |
| 1ohr | 1w5w | P212121 | P21212 | 0.31 | 0.24 | 0.07 | |  |
| 1ohr | 2fde | P212121 | P61 | 0.31 | 0.19 | 0.12 | |  |
| 1t7k | 1xl2 | P21212 | P212121 | 0.37 | 0.66 | 0.29 | |  |
| 1t7k | 2fde | P21212 | P61 | 0.37 | 0.19 | 0.18 | |  |
| 1w5v | 1xl2 | P21212 | P212121 | 0.23 | 0.66 | 0.43 | |  |
| 1w5v | 2fde | P21212 | P61 | 0.23 | 0.19 | 0.04 | |  |
| 1w5w | 1xl2 | P21212 | P212121 | 0.24 | 0.66 | 0.42 | |  |
| 1w5w | 2fde | P21212 | P61 | 0.24 | 0.19 | 0.05 | |  |
| 1w5y | 1xl2 | P21212 | P212121 | 0.24 | 0.66 | 0.42 | |  |
| 1w5y | 2fde | P21212 | P61 | 0.24 | 0.19 | 0.05 | |  |
| 1wbk | 1xl2 | P21212 | P212121 | 0.24 | 0.66 | 0.42 | |  |
| 1wbk | 2fde | P21212 | P61 | 0.24 | 0.19 | 0.05 | |  |
| 1wbm | 1xl2 | P21212 | P212121 | 0.23 | 0.66 | 0.43 | |  |
| 1wbm | 2fde | P21212 | P61 | 0.23 | 0.19 | 0.04 | |  |
| 1xl2 | 1xl5 | P212121 | P21212 | 0.66 | 0.34 | 0.32 | |  |
| 1xl2 | 2a4f | P212121 | P21212 | 0.66 | 0.32 | 0.34 | |  |
| 1xl2 | 2bb9 | P212121 | P21212 | 0.66 | 0.26 | 0.4 | |  |
| 1xl2 | 2fde | P212121 | P61 | 0.66 | 0.19 | 0.47 | |  |
| 1xl5 | 2fde | P21212 | P61 | 0.34 | 0.19 | 0.15 | |  |
| 2a4f | 2fde | P21212 | P61 | 0.32 | 0.19 | 0.13 | |  |
| 2bb9 | 2fde | P21212 | P61 | 0.26 | 0.19 | 0.07 | |  |
| 2bbb | 2fde | P21212 | P61 | 0.37 | 0.19 | 0.18 | |  |
| 2bpv | 2fde | P21212 | P61 | 0.26 | 0.19 | 0.07 | |  |
| 2bpw | 2fde | P21212 | P61 | 0.28 | 0.19 | 0.09 | |  |
| 2bpx | 2fde | P21212 | P61 | 0.36 | 0.19 | 0.17 | |  |
| 2bpy | 2fde | P21212 | P61 | 0.28 | 0.19 | 0.09 | |  |
| 2bpz | 2fde | P21212 | P61 | 0.29 | 0.19 | 0.1 | |  |
| 2bqv | 2fde | P21212 | P61 | 0.25 | 0.19 | 0.06 | |  |
| 2cej | 2fde | P21212 | P61 | 0.32 | 0.19 | 0.13 | |  |
| 2cem | 2fde | P21212 | P61 | 0.24 | 0.19 | 0.05 | |  |
| 2cen | 2fde | P21212 | P61 | 0.25 | 0.19 | 0.06 | |  |
| 2fde | 7upj | P61 | P21212 | 0.19 | 0.31 | 0.12 | |  |
| 1mm7_1 | 2al4_1 | P21212 | P1211 | 0.21 | 0.2 | 0.01 | |  |
| 1mm7_1 | 2al4_3 | P21212 | P1211 | 0.21 | 0.2 | 0.01 | |  |
| 1ms7_1 | 2al4_1 | P21212 | P1211 | 0.18 | 0.2 | 0.02 | |  |
| 1ms7_1 | 2al4_3 | P21212 | P1211 | 0.18 | 0.2 | 0.02 | |  |
| 1ms7_2 | 2al4_1 | P21212 | P1211 | 0 | 0.2 | 0.2 | |  |
| 1ms7_2 | 2al4_3 | P21212 | P1211 | 0 | 0.2 | 0.2 | |  |
| 1mxu_1 | 2al4_1 | P21212 | P1211 | 0.31 | 0.2 | 0.11 | |  |
| 1mxu_1 | 2al4_3 | P21212 | P1211 | 0.31 | 0.2 | 0.11 | |  |
| 1mxv_1 | 2al4_1 | P21212 | P1211 | 0.17 | 0.2 | 0.03 | |  |
| 1mxv_1 | 2al4_3 | P21212 | P1211 | 0.17 | 0.2 | 0.03 | |  |
| 1mxw_1 | 2al4_1 | P21212 | P1211 | 0.17 | 0.2 | 0.03 | |  |
| 1mxw_1 | 2al4_3 | P21212 | P1211 | 0.17 | 0.2 | 0.03 | |  |
| 1mxx_1 | 2al4_1 | P21212 | P1211 | 0.18 | 0.2 | 0.02 | |  |
| 1mxx_1 | 2al4_3 | P21212 | P1211 | 0.18 | 0.2 | 0.02 | |  |
| 1mxy_1 | 2al4_1 | P21212 | P1211 | 0.16 | 0.2 | 0.04 | |  |
| 1mxy_1 | 2al4_3 | P21212 | P1211 | 0.16 | 0.2 | 0.04 | |  |
| 1mxz_1 | 2al4_1 | P21212 | P1211 | 0.14 | 0.2 | 0.06 | |  |
| 1mxz_1 | 2al4_3 | P21212 | P1211 | 0.14 | 0.2 | 0.06 | |  |
| 1mxz_2 | 2al4_1 | P21212 | P1211 | 0 | 0.2 | 0.2 | |  |
| 1mxz_2 | 2al4_3 | P21212 | P1211 | 0 | 0.2 | 0.2 | |  |
| 1my0_1 | 2al4_1 | P21212 | P1211 | 0.15 | 0.2 | 0.05 | |  |
| 1my0_1 | 2al4_3 | P21212 | P1211 | 0.15 | 0.2 | 0.05 | |  |
| 1my0_2 | 2al4_1 | P21212 | P1211 | 0 | 0.2 | 0.2 | |  |
| 1my0_2 | 2al4_3 | P21212 | P1211 | 0 | 0.2 | 0.2 | |  |
| 1my1_1 | 2al4_1 | P21212 | P1211 | 0.14 | 0.2 | 0.06 | |  |
| 1my1_1 | 2al4_3 | P21212 | P1211 | 0.14 | 0.2 | 0.06 | |  |
| 1my1_2 | 2al4_1 | P21212 | P1211 | 0 | 0.2 | 0.2 | |  |
| 1my1_2 | 2al4_3 | P21212 | P1211 | 0 | 0.2 | 0.2 | |  |
| 1my2_1 | 2al4_1 | P21212 | P1211 | 0.14 | 0.2 | 0.06 | |  |
| 1my2_1 | 2al4_3 | P21212 | P1211 | 0.14 | 0.2 | 0.06 | |  |
| 1my2_2 | 2al4_1 | P21212 | P1211 | 0 | 0.2 | 0.2 | |  |
| 1my2_2 | 2al4_3 | P21212 | P1211 | 0 | 0.2 | 0.2 | |  |
| 1my3_1 | 2al4_1 | P21212 | P1211 | 0.28 | 0.2 | 0.08 | |  |
| 1my3_1 | 2al4_3 | P21212 | P1211 | 0.28 | 0.2 | 0.08 | |  |
| 1my4_1 | 2al4_1 | P21212 | P1211 | 0.3 | 0.2 | 0.1 | |  |
| 1my4_1 | 2al4_3 | P21212 | P1211 | 0.3 | 0.2 | 0.1 | |  |
| 1adb | 1bto_1 | P1 | P1211 | 0.16 | 0.1 | 0.06 | |  |
| 1adb | 1bto_2 | P1 | P1211 | 0.16 | 0.1 | 0.06 | |  |
| 1adb | 1hld | P1 | P1211 | 0.16 | 0.14 | 0.02 | |  |
| 1adc | 1bto_1 | P1 | P1211 | 0.12 | 0.1 | 0.02 | |  |
| 1adc | 1bto_2 | P1 | P1211 | 0.12 | 0.1 | 0.02 | |  |
| 1adc | 1hld | P1 | P1211 | 0.12 | 0.14 | 0.02 | |  |
| 1bto_1 | 1n92 | P1211 | P1 | 0.1 | 0.08 | 0.02 | |  |
| 1bto_1 | 2ohx | P1211 | P1 | 0.1 | 0.18 | 0.08 | |  |
| 1bto_2 | 1n92 | P1211 | P1 | 0.1 | 0.08 | 0.02 | |  |
| 1bto_2 | 2ohx | P1211 | P1 | 0.1 | 0.18 | 0.08 | |  |
| 1hld | 1n92 | P1211 | P1 | 0.14 | 0.08 | 0.06 | |  |
| 1hld | 2ohx | P1211 | P1 | 0.14 | 0.18 | 0.04 | |  |
| 1ldy_1 | 1n92 | P1211 | P1 | 0.1 | 0.08 | 0.02 | |  |
| 1ldy_1 | 2ohx | P1211 | P1 | 0.1 | 0.18 | 0.08 | |  |
| 1ldy_2 | 1n92 | P1211 | P1 | 0.1 | 0.08 | 0.02 | |  |
| 1ldy_2 | 2ohx | P1211 | P1 | 0.1 | 0.18 | 0.08 | |  |
| 1mg0_1 | 1n92 | P1211 | P1 | 0.15 | 0.08 | 0.07 | |  |
| 1mg0_1 | 2ohx | P1211 | P1 | 0.15 | 0.18 | 0.03 | |  |
| 1mg0_2 | 1n92 | P1211 | P1 | 0.15 | 0.08 | 0.07 | |  |
| 1mg0_2 | 2ohx | P1211 | P1 | 0.15 | 0.18 | 0.03 | |  |
| 1n92 | 1p1r_1 | P1 | P1211 | 0.08 | 0.14 | 0.06 | |  |
| 1n92 | 1p1r_2 | P1 | P1211 | 0.08 | 0.12 | 0.04 | |  |
| 1n92 | 2oxi | P1 | P1211 | 0.08 | 0.14 | 0.06 | |  |
| 1p1r_1 | 2ohx | P1211 | P1 | 0.14 | 0.18 | 0.04 | |  |
| 1p1r_2 | 2ohx | P1211 | P1 | 0.12 | 0.18 | 0.06 | |  |
| 2ohx | 2oxi | P1 | P1211 | 0.18 | 0.14 | 0.04 | |  |
| 2ohx | 3bto_1 | P1 | P1211 | 0.18 | 0.14 | 0.04 | |  |
| 2ohx | 3bto_2 | P1 | P1211 | 0.18 | 0.14 | 0.04 | |  |
| 19gs | 21gs | C121 |  | 0.05 | 0.05 | 0 | |  |
| 1zgn | 21gs | C121 |  | 0.16 | 0.05 | 0.11 | |  |
| 20gs | 21gs | C121 |  | 0.05 | 0.05 | 0 | |  |
| 21gs | 2gss |  | C121 | 0.05 | 0.05 | 0 | |  |
| 21gs | 3gss |  | C121 | 0.05 | 0.07 | 0.02 | |  |
| 21gs | 5gss |  | C121 | 0.05 | 0.02 | 0.03 | |  |
| 1ag1 | 1tpd_1 | P212121 | C121 | 0.3 | 0 | 0.3 | |  |
| 1ag1 | 1tpd_2 | P212121 | C121 | 0.3 | 0 | 0.3 | |  |
| 1ag1 | 1trd_1 | P212121 | C121 | 0.3 | 0 | 0.3 | |  |
| 1iig | 1tpd_1 | P212121 | C121 | 0.3 | 0 | 0.3 | |  |
| 1iig | 1tpd_2 | P212121 | C121 | 0.3 | 0 | 0.3 | |  |
| 1iig | 1trd_1 | P212121 | C121 | 0.3 | 0 | 0.3 | |  |
| 1iih | 1tpd_1 | P212121 | C121 | 0.31 | 0 | 0.31 | |  |
| 1iih | 1tpd_2 | P212121 | C121 | 0.31 | 0 | 0.31 | |  |
| 1iih | 1trd_1 | P212121 | C121 | 0.31 | 0 | 0.31 | |  |
| 1tpd_1 | 4tim | C121 | P212121 | 0 | 0.29 | 0.29 | |  |
| 1tpd_1 | 5tim | C121 | P212121 | 0 | 0.27 | 0.27 | |  |
| 1tpd_1 | 6tim | C121 | P212121 | 0 | 0.32 | 0.32 | |  |
| 1tpd_2 | 4tim | C121 | P212121 | 0 | 0.29 | 0.29 | |  |
| 1tpd_2 | 5tim | C121 | P212121 | 0 | 0.27 | 0.27 | |  |
| 1tpd_2 | 6tim | C121 | P212121 | 0 | 0.32 | 0.32 | |  |
| 1trd_1 | 4tim | C121 | P212121 | 0 | 0.29 | 0.29 | |  |
| 1trd_1 | 5tim | C121 | P212121 | 0 | 0.27 | 0.27 | |  |
| 1trd_1 | 6tim | C121 | P212121 | 0 | 0.32 | 0.32 | |  |
| 1trd_2 | 4tim | C121 | P212121 | 0 | 0.29 | 0.29 | |  |
| 1trd_2 | 5tim | C121 | P212121 | 0 | 0.27 | 0.27 | |  |
| 1trd_2 | 6tim | C121 | P212121 | 0 | 0.32 | 0.32 | |  |
| 2g5p | 2oqv | P1211 | P212121 | 0.1 | 0.34 | 0.24 | |  |
| 2g5t | 2oqv | P1211 | P212121 | 0.3 | 0.34 | 0.04 | |  |
| 2g63_1 | 2oqv | P1211 | P212121 | 0.13 | 0.34 | 0.21 | |  |
| 2g63_2 | 2oqv | P1211 | P212121 | 0.05 | 0.34 | 0.29 | |  |
| 2i03_1 | 2oqv | P1211 | P212121 | 0.31 | 0.34 | 0.03 | |  |
| 2i03_2 | 2oqv | P1211 | P212121 | 0.13 | 0.34 | 0.21 | |  |
| 2oqi_1 | 2oqv | P1211 | P212121 | 0.14 | 0.34 | 0.2 | |  |
| 2oqi_2 | 2oqv | P1211 | P212121 | 0.05 | 0.34 | 0.29 | |  |
| 1mm7_2 | 2al4_2 | P21212 | P1211 | 0 | 0.01 | 0.01 | |  |
| 1mxu_2 | 2al4_2 | P21212 | P1211 | 0 | 0.01 | 0.01 | |  |
| 1mxv_2 | 2al4_2 | P21212 | P1211 | 0 | 0.01 | 0.01 | |  |
| 1mxw_2 | 2al4_2 | P21212 | P1211 | 0 | 0.01 | 0.01 | |  |
| 1mxx_2 | 2al4_2 | P21212 | P1211 | 0 | 0.01 | 0.01 | |  |
| 1mxy_2 | 2al4_2 | P21212 | P1211 | 0 | 0.01 | 0.01 | |  |
| 1my3_2 | 2al4_2 | P21212 | P1211 | 0 | 0.01 | 0.01 | |  |
| 1my4_2 | 2al4_2 | P21212 | P1211 | 0 | 0.01 | 0.01 | |  |
| 1cbj | 1sda_1 | P212121 | C121 | 0.16 | 0.25 | 0.09 | |  |
| 1cbj | 1sda_2 | P212121 | C121 | 0.16 | 0.19 | 0.03 | |  |
| 1cbj | 2sod_1 | P212121 | C121 | 0.16 | 0.44 | 0.28 | |  |
| 1cbj | 1sxn | P212121 | C2221 | 0.16 | 0.17 | 0.01 | |  |
| 1cbj | 1sxs | P212121 | C2221 | 0.16 | 0.18 | 0.02 | |  |
| 1cbj | 1sxz | P212121 | C2221 | 0.16 | 0.17 | 0.01 | |  |
| 1cob | 1sda_1 | P212121 | C121 | 0.17 | 0.25 | 0.08 | |  |
| 1cob | 1sda_2 | P212121 | C121 | 0.17 | 0.19 | 0.02 | |  |
| 1cob | 2sod_1 | P212121 | C121 | 0.17 | 0.44 | 0.27 | |  |
| 1cob | 1sxn | P212121 | C2221 | 0.17 | 0.17 | 0 | |  |
| 1cob | 1sxs | P212121 | C2221 | 0.17 | 0.18 | 0.01 | |  |
| 1cob | 1sxz | P212121 | C2221 | 0.17 | 0.17 | 0 | |  |
| 1sda_1 | 1sxn | C121 | C2221 | 0.25 | 0.17 | 0.08 | |  |
| 1sda_1 | 1sxs | C121 | C2221 | 0.25 | 0.18 | 0.07 | |  |
| 1sda_1 | 1sxz | C121 | C2221 | 0.25 | 0.17 | 0.08 | |  |
| 1sda_2 | 1sxn | C121 | C2221 | 0.19 | 0.17 | 0.02 | |  |
| 1sda_2 | 1sxs | C121 | C2221 | 0.19 | 0.18 | 0.01 | |  |
| 1sda_2 | 1sxz | C121 | C2221 | 0.19 | 0.17 | 0.02 | |  |
| 1sxn | 2sod_1 | C2221 | C121 | 0.17 | 0.44 | 0.27 | |  |
| 1sxn | 2sod_2 | C2221 | C121 | 0.17 | 0.45 | 0.28 | |  |
| 1sxs | 2sod_1 | C2221 | C121 | 0.18 | 0.44 | 0.26 | |  |
| 1sxs | 2sod_2 | C2221 | C121 | 0.18 | 0.45 | 0.27 | |  |
| 1sxz | 2sod_1 | C2221 | C121 | 0.17 | 0.44 | 0.27 | |  |
| 1sxz | 2sod_2 | C2221 | C121 | 0.17 | 0.45 | 0.28 | |  |
| 1eby | 1pro | P21212 | P61 | 0.24 | 0.08 | 0.16 | |  |
| 1eby | 1sbg | P21212 | P61 | 0.24 | 0.19 | 0.05 | |  |
| 1eby | 1vij | P21212 | P61 | 0.24 | 0.31 | 0.07 | |  |
| 1hxw | 1pro | P21212 | P61 | 0.36 | 0.08 | 0.28 | |  |
| 1hxw | 1sbg | P21212 | P61 | 0.36 | 0.19 | 0.17 | |  |
| 1hxw | 1vij | P21212 | P61 | 0.36 | 0.31 | 0.05 | |  |
| 1izh | 1pro | P21212 | P61 | 0.25 | 0.08 | 0.17 | |  |
| 1izh | 1sbg | P21212 | P61 | 0.25 | 0.19 | 0.06 | |  |
| 1izh | 1vij | P21212 | P61 | 0.25 | 0.31 | 0.06 | |  |
| 1pro | 1vik | P61 | P21212 | 0.08 | 0.37 | 0.29 | |  |
| 1pro | 4phv | P61 | P21212 | 0.08 | 0.25 | 0.17 | |  |
| 1sbg | 1vik | P61 | P21212 | 0.19 | 0.37 | 0.18 | |  |
| 1sbg | 4phv | P61 | P21212 | 0.19 | 0.25 | 0.06 | |  |
| 1vij | 1vik | P61 | P21212 | 0.31 | 0.37 | 0.06 | |  |
| 1vij | 4phv | P61 | P21212 | 0.31 | 0.25 | 0.06 | |  |
| 1vik | 9hvp | P21212 | P61 | 0.37 | 0.27 | 0.1 | |  |
| 4phv | 9hvp | P21212 | P61 | 0.25 | 0.27 | 0.02 | |  |
| 1arg | 1x28 | P1211 | P63 | 0.11 | 0.2 | 0.09 | |  |
| 1arg | 1x29 | P1211 | P63 | 0.11 | 0.18 | 0.07 | |  |
| 1arg | 1x2a | P1211 | P63 | 0.11 | 0.18 | 0.07 | |  |
| 1asl | 1x28 | P1211 | P63 | 0.19 | 0.2 | 0.01 | |  |
| 1asl | 1x29 | P1211 | P63 | 0.19 | 0.18 | 0.01 | |  |
| 1asl | 1x2a | P1211 | P63 | 0.19 | 0.18 | 0.01 | |  |
| 1asm | 1x28 | P1211 | P63 | 0.14 | 0.2 | 0.06 | |  |
| 1asm | 1x29 | P1211 | P63 | 0.14 | 0.18 | 0.04 | |  |
| 1asm | 1x2a | P1211 | P63 | 0.14 | 0.18 | 0.04 | |  |
| 1asn | 1x28 | P1211 | P63 | 0.14 | 0.2 | 0.06 | |  |
| 1asn | 1x29 | P1211 | P63 | 0.14 | 0.18 | 0.04 | |  |
| 1asn | 1x2a | P1211 | P63 | 0.14 | 0.18 | 0.04 | |  |
| 1m6h | 1teh_1 | P43212 | C2221 | 0.18 | 0 | 0.18 | |  |
| 1m6h | 1teh_2 | P43212 | C2221 | 0.18 | 0 | 0.18 | |  |
| 1m6w | 1teh_1 | P43212 | C2221 | 0.2 | 0 | 0.2 | |  |
| 1m6w | 1teh_2 | P43212 | C2221 | 0.2 | 0 | 0.2 | |  |
| 1ma0 | 1teh_1 | P43212 | C2221 | 0.17 | 0 | 0.17 | |  |
| 1ma0 | 1teh_2 | P43212 | C2221 | 0.17 | 0 | 0.17 | |  |
| 1mc5 | 1teh_1 | P43212 | C2221 | 0.49 | 0 | 0.49 | |  |
| 1mc5 | 1teh_2 | P43212 | C2221 | 0.49 | 0 | 0.49 | |  |
| 1mp0 | 1teh_1 | P43212 | C2221 | 0.15 | 0 | 0.15 | |  |
| 1mp0 | 1teh_2 | P43212 | C2221 | 0.15 | 0 | 0.15 | |  |
| 1hii | 1hsh_1 | P212121 | P1211 | 0.25 | 0.36 | 0.11 | |  |
| 1hii | 1hsh_2 | P212121 | P1211 | 0.25 | 0.23 | 0.02 | |  |
| 1hii | 1hsi | P212121 | P1211 | 0.25 | 0.33 | 0.08 | |  |
| 1hii | 1ida | P212121 | P43212 | 0.25 | 0.17 | 0.08 | |  |
| 1hii | 1idb | P212121 | P43212 | 0.25 | 0.19 | 0.06 | |  |
| 1hii | 1jld | P212121 | P43212 | 0.25 | 0.22 | 0.03 | |  |
| 1hsh_1 | 1ida | P1211 | P43212 | 0.36 | 0.17 | 0.19 | |  |
| 1hsh_1 | 1idb | P1211 | P43212 | 0.36 | 0.19 | 0.17 | |  |
| 1hsh_1 | 1jld | P1211 | P43212 | 0.36 | 0.22 | 0.14 | |  |
| 1hsh_2 | 1ida | P1211 | P43212 | 0.23 | 0.17 | 0.06 | |  |
| 1hsh_2 | 1idb | P1211 | P43212 | 0.23 | 0.19 | 0.04 | |  |
| 1hsh_2 | 1jld | P1211 | P43212 | 0.23 | 0.22 | 0.01 | |  |
| 1hsi | 1ida | P1211 | P43212 | 0.33 | 0.17 | 0.16 | |  |
| 1hsi | 1idb | P1211 | P43212 | 0.33 | 0.19 | 0.14 | |  |
| 1hsi | 1jld | P1211 | P43212 | 0.33 | 0.22 | 0.11 | |  |
| 1oxo | 1tas | P1 | P1211 | 0.23 | 0.15 | 0.08 | |  |
| 1tar | 1tas | P1 | P1211 | 0.16 | 0.15 | 0.01 | |  |
| 1tas | 7aat | P1211 | P1 | 0.15 | 0.2 | 0.05 | |  |
| 1tas | 8aat | P1211 | P1 | 0.15 | 0.21 | 0.06 | |  |
| 1tas | 9aat | P1211 | P1 | 0.15 | 0.19 | 0.04 | |  |
| 1vga_1 | 1woa_1 | P1211 | P212121 | 0.15 | 0.03 | 0.12 | |  |
| 1vga_1 | 1woa_2 | P1211 | P212121 | 0.15 | 0.04 | 0.11 | |  |
| 1vga_1 | 1wob_1 | P1211 | P212121 | 0.15 | 0.1 | 0.05 | |  |
| 1vga_2 | 1woa_1 | P1211 | P212121 | 0.18 | 0.03 | 0.15 | |  |
| 1vga_2 | 1woa_2 | P1211 | P212121 | 0.18 | 0.04 | 0.14 | |  |
| 1vga_2 | 1wob_1 | P1211 | P212121 | 0.18 | 0.1 | 0.08 | |  |
| 1pu0_1 | 1spd | C2221 | P63 | 0.2 | 0.59 | 0.39 | |  |
| 1pu0_2 | 1spd | C2221 | P63 | 0.26 | 0.59 | 0.33 | |  |
| 1pu0_3 | 1spd | C2221 | P63 | 0.11 | 0.59 | 0.48 | |  |
| 1pu0_4 | 1spd | C2221 | P63 | 0.27 | 0.59 | 0.32 | |  |
| 1pu0_5 | 1spd | C2221 | P63 | 0.14 | 0.59 | 0.45 | |  |
| 1hbi | 4sdh | C121 | C2221 | 0.2 | 0.15 | 0.05 | |  |
| 1hbi | 1nwi_1 | C121 | P1211 | 0.2 | 0.2 | 0 | |  |
| 1hbi | 1nwi_2 | C121 | P1211 | 0.2 | 0.12 | 0.08 | |  |
| 1nwi_1 | 1nwn | P1211 | C121 | 0.2 | 0.02 | 0.18 | |  |
| 1nwi_1 | 3sdh | P1211 | C121 | 0.2 | 0.19 | 0.01 | |  |
| 1nwi_1 | 4sdh | P1211 | C2221 | 0.2 | 0.15 | 0.05 | |  |
| 1nwi_2 | 1nwn | P1211 | C121 | 0.12 | 0.02 | 0.1 | |  |
| 1nwi_2 | 3sdh | P1211 | C121 | 0.12 | 0.19 | 0.07 | |  |
| 1nwi_2 | 4sdh | P1211 | C2221 | 0.12 | 0.15 | 0.03 | |  |
| 1nwn | 4sdh | C121 | C2221 | 0.02 | 0.15 | 0.13 | |  |
| 3sdh | 4sdh | C121 | C2221 | 0.19 | 0.15 | 0.04 | |  |
| 1mo9 | 1mok_1 | P1211 | P1 | 0.23 | 0.17 | 0.06 | |  |
| 1mo9 | 1mok_2 | P1211 | P1 | 0.23 | 0.17 | 0.06 | |  |
| 1mok_1 | 2c3c | P1 | P1211 | 0.17 | 0.18 | 0.01 | |  |
| 1mok_1 | 2c3d | P1 | P1211 | 0.17 | 0.09 | 0.08 | |  |
| 1mok_2 | 2c3c | P1 | P1211 | 0.17 | 0.18 | 0.01 | |  |
| 1mok_2 | 2c3d | P1 | P1211 | 0.17 | 0.09 | 0.08 | |  |
| 1lbv | 1lbw | P1211 | P32 | 0.46 | 0.27 | 0.19 | |  |
| 1lbv | 1lby | P1211 | P32 | 0.46 | 0.27 | 0.19 | |  |
| 1lbv | 1lbz | P1211 | P32 | 0.46 | 0.34 | 0.12 | |  |
| 1lbw | 1lbx | P32 | P1211 | 0.27 | 0.31 | 0.04 | |  |
| 1lbx | 1lby | P1211 | P32 | 0.31 | 0.27 | 0.04 | |  |
| 1lbx | 1lbz | P1211 | P32 | 0.31 | 0.34 | 0.03 | |  |
| 1lzo_1 | 1ydv | P212121 | C121 | 0.27 | 0.27 | 0 | |  |
| 1lzo_1 | 1m7o | P212121 | P1211 | 0.27 | 0.18 | 0.09 | |  |
| 1lzo_1 | 1m7p | P212121 | P1211 | 0.27 | 0.17 | 0.1 | |  |
| 1lzo_2 | 1ydv | P212121 | C121 | 0.23 | 0.27 | 0.04 | |  |
| 1lzo_2 | 1m7o | P212121 | P1211 | 0.23 | 0.18 | 0.05 | |  |
| 1lzo_2 | 1m7p | P212121 | P1211 | 0.23 | 0.17 | 0.06 | |  |
| 1m7o | 1ydv | P1211 | C121 | 0.18 | 0.27 | 0.09 | |  |
| 1m7p | 1ydv | P1211 | C121 | 0.17 | 0.27 | 0.1 | |  |
| 1xw5 | 1ykc | P1211 | P212121 | 0.12 | 0.12 | 0 | |  |
| 1xw5 | 2ab6_1 | P1211 | P212121 | 0.12 | 0.07 | 0.05 | |  |
| 1xw5 | 2ab6_2 | P1211 | P212121 | 0.12 | 0.17 | 0.05 | |  |
| 1ykc | 2gtu | P212121 | P1211 | 0.12 | 0.1 | 0.02 | |  |
| 2ab6_1 | 2gtu | P212121 | P1211 | 0.07 | 0.1 | 0.03 | |  |
| 2ab6_2 | 2gtu | P212121 | P1211 | 0.17 | 0.1 | 0.07 | |  |
| 1jm0_1 | 1jmb_1 | P212121 | C2221 | 0.26 | 0 | 0.26 | |  |
| 1jm0_1 | 1jmb_2 | P212121 | C2221 | 0.26 | 0.23 | 0.03 | |  |
| 1jm0_2 | 1jmb_1 | P212121 | C2221 | 0.46 | 0 | 0.46 | |  |
| 1jm0_2 | 1jmb_2 | P212121 | C2221 | 0.46 | 0.23 | 0.23 | |  |
| 1jm0_3 | 1jmb_1 | P212121 | C2221 | 0.42 | 0 | 0.42 | |  |
| 1jm0_3 | 1jmb_2 | P212121 | C2221 | 0.42 | 0.23 | 0.19 | |  |
| 1ekf | 1ekp | P212121 | P1211 | 0.32 | 0.24 | 0.08 | |  |
| 1ekf | 1ekv | P212121 | P32 | 0.32 | 0.14 | 0.18 | |  |
| 1ekp | 1kta | P1211 | P212121 | 0.24 | 0.34 | 0.1 | |  |
| 1ekp | 1ekv | P1211 | P32 | 0.24 | 0.14 | 0.1 | |  |
| 1ekv | 1kta | P32 | P212121 | 0.14 | 0.34 | 0.2 | |  |
| 1daa | 4daa | P1211 | C121 | 0.3 | 0.13 | 0.17 | |  |
| 1daa | 2daa | P1211 | P212121 | 0.3 | 0.22 | 0.08 | |  |
| 1daa | 3daa | P1211 | P212121 | 0.3 | 0.23 | 0.07 | |  |
| 2daa | 4daa | P212121 | C121 | 0.22 | 0.13 | 0.09 | |  |
| 3daa | 4daa | P212121 | C121 | 0.23 | 0.13 | 0.1 | |  |
| 1glp | 1gsy | P212121 | C2221 | 0.13 | 0.24 | 0.11 | |  |
| 1glq | 1gsy | P212121 | C2221 | 0.1 | 0.24 | 0.14 | |  |
| 1gsy | 2glr | C2221 | P212121 | 0.24 | 0.13 | 0.11 | |  |
| 1sla | 1slb_1 | P6322 | C121 | 0.33 | 0.41 | 0.08 | |  |
| 1sla | 1slb_2 | P6322 | C121 | 0.33 | 0.4 | 0.07 | |  |
| 1sla | 1slc_1 | P6322 | P31 | 0.33 | 0.25 | 0.08 | |  |
| 1slb_1 | 1slc_1 | C121 | P31 | 0.41 | 0.25 | 0.16 | |  |
| 1slb_2 | 1slc_1 | C121 | P31 | 0.4 | 0.25 | 0.15 | |  |
| 11bg | 1r5c | P22121 | P212121 | 0.47 | 0.32 | 0.15 | |  |
| 11bg | 1r5d | P22121 | P212121 | 0.47 | 0.34 | 0.13 | |  |
| 1bsr | 1r5c | P22121 | P212121 | 0.54 | 0.32 | 0.22 | |  |
| 1bsr | 1r5d | P22121 | P212121 | 0.54 | 0.34 | 0.2 | |  |
| 1ksi | 1w2z_1 | P212121 | P1211 | 0.1 | 0.07 | 0.03 | |  |
| 1ksi | 1w2z_2 | P212121 | P1211 | 0.1 | 0.09 | 0.01 | |  |
| 1w4n | 2cfd | I121 | C121 | 0.1 | 0.12 | 0.02 | |  |
| 1w4n | 2cfg | I121 | C121 | 0.1 | 0.05 | 0.05 | |  |
| 3pgh_1 | 6cox | P21212 | I222 | 0.03 | 0.03 | 0 | |  |
| 3pgh_2 | 6cox | P21212 | I222 | 0.03 | 0.03 | 0 | |  |
| 1t4b | 1t4d_1 | P212121 | P21212 | 0.35 | 0.15 | 0.2 | |  |
| 1t4b | 1t4d_2 | P212121 | P21212 | 0.35 | 0 | 0.35 | |  |
| 1bko_1 | 1bsf | P1211 | P212121 | 0.27 | 0.12 | 0.15 | |  |
| 1bko_2 | 1bsf | P1211 | P212121 | 0.23 | 0.12 | 0.11 | |  |
| 7gss | 8gss_1 | P212121 | C121 | 0.04 | 0.19 | 0.15 | |  |
| 7gss | 8gss_2 | P212121 | C121 | 0.04 | 0 | 0.04 | |  |
| 1fro_1 | 1qin | P43 | P1211 | 0.02 | 0.03 | 0.01 | |  |
| 1fro_2 | 1qin | P43 | P1211 | 0.02 | 0.03 | 0.01 | |  |
| 1wmy | 1wmz_1 | C121 | P1211 | 0.4 | 0.54 | 0.14 | |  |
| 1wmy | 1wmz_2 | C121 | P1211 | 0.4 | 0.75 | 0.35 | |  |
| 1coz | 1n1d_1 | P1211 | P1 | 0.08 | 0.23 | 0.15 | |  |
| 1coz | 1n1d_2 | P1211 | P1 | 0.08 | 0.4 | 0.32 | |  |
| 1c6x | 1c6y | P212121 | P21212 | 0.14 | 0.39 | 0.25 | |  |
| 1c6y | 1c6z | P21212 | P212121 | 0.39 | 0.24 | 0.15 | |  |
| 1gu7 | 1guf | P1211 | P3121 | 0.53 | 0.4 | 0.13 | |  |
| 1p1q_1 | 1p1u | P21212 | P212121 | 0.16 | 0.92 | 0.76 | |  |
| 1ks2 | 1lkz | P1 | C2221 | 0.74 | 0.09 | 0.65 | |  |
| 2oa7 | 2oac | C121 | P212121 | 0.17 | 0.16 | 0.01 | |  |
| 1c7z | 1c80 | P1211 | C121 | 0.26 | 0.29 | 0.03 | |  |
| 1knq | 1kof | P212121 | P1211 | 0.66 | 0.23 | 0.43 | |  |
| 2av0 | 2av3 | C121 | C2221 | 0.19 | 0.15 | 0.04 | |  |
| 4hbi | 5hbi | C2221 | C121 | 0.17 | 0.2 | 0.03 | |  |
| 6hbi | 7hbi | C2221 | C121 | 0.18 | 0.2 | 0.02 | |  |
| 1hxb | 1odw | P61 | P212121 | 0.12 | 0.33 | 0.21 | |  |
| 1siv | 1tcw | I222 | P1211 | 0.34 | 0.42 | 0.08 | |  |

In the PDB codes, “_1” and “_2” refers to the biological unit under consideration, for cases where multiple homodimeric biological units are available for a PDB.
